
JOURNAL INFORMATION
==============================
NLM Title Abbreviation: Eur Psychiatry
Journal ID: Eur Psychiatry
NLM Journal ID: 9111820
Journal ID: EPA

European Psychiatry

ISSN: 0924-9338
EISSN: 1778-3585
Publisher: Cambridge University Press

ARTICLE INFORMATION
==============================
PMCID: PMC9412725
DOI: 10.1192/j.eurpsy.2020.52
Article ID: S0924933820000528
Article version: 1
Subjects: Abstracts

Oral Communication

Publication date: 2020 Jul
Electronic publication date: 2020 Sep 4
Volume: 63
Issue: Suppl 1
Issue title: Abstracts of the 28th European Congress of Psychiatry
First page: S3
Last page: S44
Copyright: © The Author(s) 2020
Copyright year: 2020
Copyright holder: The Author(s)
License: This is an Open Access article, distributed under the terms of the Creative Commons Attribution licence (http://creativecommons.org/licenses/by/4.0/), which permits unrestricted re-use, distribution, and reproduction in any medium, provided the original work is properly cited.
License URL: https://creativecommons.org/licenses/by/4.0/

Figure count: 35
Table count: 3
Page count: 42

==============================

### ORAL COMMUNICATION SESSION 01: ANXIETY DISORDERS AND SOMATOFORM DISORDERS / BIPOLAR DISORDERS / CLASSIFICATION OF MENTAL DISORDERS

Abstracts

EUROPEAN PSYCHIATRIC ASSOCIATION

#### O0001

#### Further validation of the portuguese version of the modified dental anxiety scale

A.T. Pereira, A. Araújo, C. Cabaços*, M.J. Soares, S. Xavier, C. Marques, P. Lopes and A. Macedo

Portugal

**Introduction:** Dental anxiety is a public health problem, affecting at least 10% of the population. The Modified Dental Anxiety Scale (MDAS; Humphris et al. 1995) is the most frequently used dental anxiety questionnaire, due to its briefness (5 items) and good psychometric properties. The Portuguese version of MDAS (Lopes 2009) presented adequate validity and reliability in a sample of university students. It is important to continue to study its psychometric features, namely with a more representative sample of the Portuguese population.
**Objectives:** To analyze the psychometric properties of the MDAS Portuguese version in a general population sample: construct validity (factor structure using Confirmatory Factor Analysis/CFA), internal consistency and concurrent validity.
**Methods:** A community sample of 437 adults (68.2% women; mean age= 35.15±15.790; range:18-88 years) completed the Portuguese versions of: MDAS, Dental Fear Survey (Lopes 2009) and State-Trait Anxiety Inventory (Silva et al. 2006).
**Results:** CFA indicated a good fit for the unidimensional model (X2/df=3.239; CFI=.994; GFI=.988; TLI=.985; p[RMSEA≤.01]=.072), as well as for the second-order model with two factors (X2/df=3.239; CFI=.991; GFI=.987; TLI=.988; p[RMSEA≤.01]=.070). MDAS Cronbach's alpha was of $\alpha$=.903; for F1 Anticipatory Anxiety and F2 Treatment Related Anxiety were $\alpha$=.885 and $\alpha$=.866, respectively. MDAS total and dimensional scores significantly and highly correlated (r=.70) with all DFS measures and moderately with trait-anxiety (r=.30).
**Conclusions:** This additional validation study emphasizes that MDAS is a valid and reliable measure of dental anxiety. In the near future we will determine the MDAS cut-off to screen for dental anxiety disorders.

#### O0002

#### Impact of childhood trauma and attachment styles on resilience in euthymic patients with bipolar disorder

C. Citak* and E. Erten

Turkey

**Introduction:** Adverse experiences in childhood negatively affect the development of resilience and of secure attachment, and these experiences have been associated with worse course of illness in patients with bipolar disorder.
**Objectives:** We aimed to examine impact of childhood trauma and attachment styles on clinical features and mediating role of resilience on this impact in patients who have been diagnosed with bipolar disorder according to DSM-5.Possible relations between childhood trauma,attachment styles and resilience were also examined.
**Methods:** The study group comprised of 110 euthymic patients with bipolar disorder.Hamilton Depression Rating Scale(HAM-D) and Young Mani Rating Scale(YMRS) are administered to verify remission.Childhood trauma questionnaire(CTQ), Experiences in Close Relationships-revised and Resilience for Adults scales administered to all patients.
**Results:** More than half of patients(%58.2) in our bipolar disorder sample reported childhood trauma.Total childhood trauma scores were associated with lower scores of resilience(r=0.412 p<0.01), higher scores of attachment-related anxiety(r=0.324 p<0.01) and avoidance(r=0.236 p<0.05). Resilience scores were negatively associated with attachment-related anxiety (r=-0.514

p<0.01) and avoidance(r=-0.372 p<0.01).Impact of childhood trauma scores on resilience scores partly mediated by attachment-related anxiety(Sobel Z=-2.463 p=0.014) and avoidance(Sobel Z=-2.295,p=0.036), respectively.
**Conclusions:** History of childhood trauma in patients with bipolar disorder is frequent.Childhood traumas are associated with lower resilience and higher attachment-related anxiety and avoidance.Impact of childhood trauma on resilience was partly mediated by attachment-related anxiety and avoidance.Since resilience is associated with increased quality of life and better clinical course even in stable patients with bipolar disorder, it might be helpful to develop attachment informed psychosocial interventions to ameliorate detrimental effects of childhood trauma on resilience.

### O0007

### Evaluating maintenance electroconvulsive therapy for relapse prevention in bipolar disorder

S. Madero*, G. Anmella, M.T. Pons, M. Sagué, A. Giménez Palomo, A. Murru, N. Arbelo, C. Llach, L. Ilzarbe, M. Bioque and A. Benabarre

Spain

**Introduction:** Electroconvulsive therapy is well established as an effective tool in psychiatry. In most cases ECT is gradually discontinued after clinical improvement, with a good remission rate if pharmacological treatment is continued. Nonetheless its estimated around 40% of patients relapse after 6 months and approximately 60% by one year.
**Objectives:** To study the impact of Maintenance Electroconvulsive therapy in the course of Type I Bipolar disorder
**Methods:** We conducted an observational mirror-image study in patients who started maintenance ECT between 2000-2016 at our center. We did a chart review of 43 clinical records that fulfilled inclusion criteria. Descriptive analyses were performed using SPSS Statistics 23 IBM, considering statistical significance as p<0,05
**Results:** The mean number of hospitalizations before maintenance ECT was 2 (±2.53), mean number of hospitalization was 35 (±56.68). While the mean number of hospitalizations during maintenance ECT was 0 ($\bar{x}$=0.74; M=0±1.48;Range=0-6), and number of hospitalization days during maintenance ECT was 0 ($\bar{x}$=11.91; M=0±28.5; Rang=0-168). Kolmogorov-Smirnov normality test showed a non Gaussian distribution of the "response" variables (p<0.0001). We performed the Wilcoxon test comparing "response" variables: number of hospitalizations "before" vs "after" (Z=-3.739; p<0.0001); and hospitalization days "before" and "after" (Z=-4.648; p<0.0001).
**Conclusions:** This study shows a significant reduction in the number of hospitalization and number of days in patients diagnosed with Bipolar Disorder undergoing maintenance ECT compared to pharmacological treatment alone. The use of this modality of treatment can help taper the socioeconomic, psychic and somatic repercussions of inpatient psychiatric care, for this reasons when indicated maintenance ECT should be taken into consideration

### O0008

### Sexual dysfunction in bipolar disorder: A systematic review

Y. Cañada Pérez[1]*, P. Navalón Rodríguez[1], R. Zazula[2], M. Berk[3], S. Dodd[3], A. García-Blanco[1] and P. Sierra San Miguel[1]

[1]Spain,; [2]Brazil, and [3]Australia

**Introduction:** Bipolar disorder (BD) implies sexual disturbances during affective phases but also during euthymia. Sexual dysfunctions (SD) in BD patients are specially pernicious, as they relate to an impact in quality of life, and a lower treatment compliance, making patients prone to new affective episodes. In spite of this, SD are typically misdiagnosed and their characteristics remain yet unclear.
**Objectives:** To review the existent literature about SD in euthymic BD patients.
**Methods:** We performed a systematic review following PRISMA guidelines (PROSPERO ID-CRD42019130095). We selected studies including BD patients over18 year-old. Sexual functioning was evaluated with a questionnaire/semistructured interview, that measured the following sexual domains: desire, arousal, orgasm, satisfaction; or was expressed with a validated diagnosis (i.e erectile dysfunction).
**Results:** The search yielded to 132 eligible full text articles, 26 of which were included in this review. See Figure 1 (flow chart) A total of 7928 patients with BD with were evaluated in the review. Prevalence of SD was heterogenous among studies (14%-81.7%) and it was influenced by the type of clinical sample, the context of the study and the treatment. The main affected sexual domain was desire and the most frequent tool used was Arizona Sexual Experiences Scale (ASEX). When compared to healthy subjects BD patients had poorer sexual functioning and sexual satisfaction.
**Conclusions:** Our review highlights the scarcity and heterogeneity of the existent literature about SD in BD. There is a need for new studies so as to reach a better comprehension of SD and implement prevention and treatment strategies.
**Disclosure:** Yolanda Cañada has had support for conference from Janssen and Lundbeck. Pablo Navalon has received support from Lundbeck and Janssen. Seetal Dodd has received grant support from the Stanley Medical Research Institute, NHMRC, Beyond Blue, ARHRF, Simons Fo

### O0009

### Depressive polarity at illness onset is associated with lifetime suicide attempts in euthymic bipolar outpatients

G. Serafini[1]*, A. Aguglia[1], F. Santi[1], G. Canepa[1], X. Gonda[2], Z. Rihmer[2], M. Pompili[1] and M. Amore[1]

[1]Italy, and [2]Hungary

**Introduction:** Differential characteristics related to first illness episode in bipolar disorder (BD) have been identified based on the current literature; however, evidence are currently inconsistent across studies.
**Objectives:** Thus, this study aimed to identify whether first depressive episode (FDE) was associated with specific clinical correlates in a large sample of BD outpatients.
**Methods:** The sample included 364 euthymic bipolar outpatients (mean age= 53.9±16.05) of which 229 (62.9%) with FDE and 135 (37.1%) with first non-depressive episode (FNDE). A detailed data collection was performed and illness histories were retraced through clinical files and lifetime computerized medical records.
**Results:** Compared to patients with FNDE, those with FDE significantly differ regarding age ($p \leq .001$), educational level ($p \leq .05$), working status ($p \leq .01$), and body mass index ($p \leq .05$). They were also more likely to have a older age at illness onset ($p \leq .005$) and first treatment ($p=.001$), recent melancholic characteristics ($p \leq .05$) and more lifetime suicide attempts ($p \leq .05$), use of non psychiatric medications in the past ($p \leq .05$), and have experienced a recent manic episode ($p \leq .05$). After logistic regression analysis adjusting for age, educational level, and working status, FDE was associated with recent melancholic features (OR=.065, $p \leq .01$), and lifetime suicide attempts (OR=8.334, $p \leq .05$).
**Conclusions:** The identification of early targets for treatment may be very useful in the stratification of different BD phenotypes in the clinical practice. The presence of FDE seems to identify a specific subgroup of BD outpatients exhibiting differential clinical characteristics.

### O0010

#### Predicting patient outcomes in psychiatric hospitals with routine data: A machine learning approach

J. Wolff*, A. Gary, D. Jung, C. Normann, K. Kaier, H. Binder, K. Domschke, A. Klimke and M. Franz
Germany

**Introduction:** Advances in machine learning could change the way health care delivery is organised. A common problem in machine learning applications is availability of data at the point of decision making.
**Objectives:** The aim of the present study was to use routine data readily available at admission to predict aspects relevant to the organization of psychiatric hospital care.
**Methods:** The study included consecutively discharged patients between 1st of January 2017 and 31st of December 2018 from nine psychiatric hospitals in Hesse, Germany. We compared the predictive performance achieved by stochastic gradient boosting (GBM) with multiple logistic regression and a naive baseline classifier. We tested the performance of our final models on unseen patients from another calendar year and from different hospitals.
**Results:** The study included 45,388 inpatient episodes. The models' performance, as measured by the area under the Receiver Operating Characteristic curve, varied strongly between the predicted outcomes, with relatively high performance in the prediction of coercive treatment (area under the curve: 0.83) and 1:1 observations (0.80) and relatively poor performance in the prediction of short length of stay (0.69) and non-response to treatment (0.65). The GBM performed slightly better than logistic regression. Both approaches were substantially better than a naive prediction based solely on basic diagnostic grouping.
**Conclusions:** The present study has shown that administrative routine data can be used to predict aspects relevant to the organisation of psychiatric hospital care. Future research should investigate the predictive performance that is necessary to provide effective assistance in clinical practice for the benefit of both staff and patients.
**Disclosure:** Professor Franz (MF) received funding from Janssen Cilag in 2014 for a project with children of parents with mental and behavioral disorders.

## ORAL COMMUNICATION SESSION 02: PSYCHOPATHOLOGY / SCHIZOPHRENIA AND OTHER PSYCHOTIC DISORDERS - PART I

### O0014

#### Validity of the spanish adult ADHD self-report scale (ASRS) expanded scale and its relationship to whole-life self-reported psychopathology

R. Artuch-Garde, M. Aznárez-Sanado, J. Bernacer*, L. Gambra, S. Magallón, I. Garcia-Arbizu, M. Vallejo-Valdivielso, S. Carrica-Ochoa and G. Arrondo
Spain

**Introduction:** An expanded 31-item version of the Adult ADHD Self-Report Scale (ASRS-31) was recently published (Ustun et al., 2017). It is composed of four subscales: inattentive (IA); hyperactive–impulsive (HI); executive function deficits (EFD); emotional dyscontrol (ED).
**Objectives:** To validate the Spanish ASRS-31 and assess its predictive capacity for self-reported psychopathology.
**Methods:** 285 adult participants under 25 answered the ASRS-31 and reported on their whole-life psychopathology
**Results:** Confirmatory factor analysis provided satisfactory results in three goodness of fit indexes (RMSEA=0.064; CFI=0.861; SRMR=0.058; $\chi 2/df=2.12$). The scale also showed good internal consistency (Cronbach's alpha ($\alpha$): $\alpha_{Total}=0.9363$, $\alpha_{IA}=0.8592$, $\alpha_{HI}=0.8431$, $\alpha_{EFD}=0.8472$, $\alpha_{ED}=0.7870$). Logistic regressions (Tables 1 and 2) confirmed that it discriminated between subjects with and without a previous diagnosis of ADHD. When including all the subscales' scores in the model, only IA and EFD showed significant predictive value. ED showed the greatest predictive value to identify subjects with any mental disorder. When including all the subscales in the model, ED predictive value remained significant for most non-developmental disorders.
**Conclusions:** The Spanish ASRS-31 version showed moderate psychometric properties. The ED subscale scores might not discriminate between ADHD and other mental disorders.

**Table 1.** Coefficients and standard errors (shown in parenthesis) obtained in different logistic regression analysis. Each regression analysis was carried out including one dependent variable (whole-life self-reported disorder=1, non-disorder=0) and one independent variable (ASRS total scale score (Total), inattentive scores (IA), hyperactive–impulsive scores (HI); executive function deficits scores (EFD) or emotional dyscontrol scores (ED)). Dependent variables included in each of the regression analyses are presented in the first column. All the analyses are corrected by gender. * indicates significant results.

| | **Total** | **IA** | **HI** | **ED** | **EFD** |
|---|---|---|---|---|---|
| **ADHD** | 0,03(0,01)* | 0,09(0,03)* | 0,09(0,03)* | 0,11(0,05)* | 0,05(0,03) |
| **Dev dis** | 0,03(0,01)* | 0,08(0,03)* | 0,1(0,03)* | 0,13(0,06)* | 0,05(0,03) |
| Dyscalculia | 0,02(0,01) | 0,07(0,04) | 0,08(0,04)* | 0,07(0,08) | 0,01(0,04) |
| Language dis | 0,03(0,01)* | 0,08(0,04) | 0,11(0,04)* | 0,23(0,09)* | 0,03(0,05) |
| **Adult dis** | 0,02(0,01)* | 0,03(0,02) | 0,05(0,02)* | 0,16(0,04)* | 0,03(0,02) |
| Depression | 0,02(0,01)* | 0,06(0,03)* | 0,05(0,03) | 0,15(0,05)* | 0,05(0,03) |
| Anxiety | 0,01(0,01) | 0,01(0,02) | 0,07(0,02)* | 0,16(0,05)* | 0,02(0,02) |
| OCD | 0,01(0,02) | 0,02(0,05) | 0,01(0,05) | 0,24(0,09)* | -0,03(0,05) |
| Subst. Abuse | 0,31(0,02)* | 0,07(0,05) | 0,85(0,05) | 0,24(0,09)* | 0,08(0,05) |

**Table 2.** Coefficients and standard errors (shown in parenthesis) obtained in different logistic regression analysis. Each regression analysis was carried out including one dependent variable (whole-life self-reported disorder=1, non-disorder=0) and four independent variables (inattentive scores (IA); hyperactive–impulsive scores (HI); executive function deficits scores (EFD); emotional dyscontrol scores (ED)). Dependent variables included in each of the regression analyses are presented in the first column. All the analyses are corrected by gender. * indicates significant results.

| | **IA** | **HI** | **ED** | **EFD** |
|---|---|---|---|---|
| **ADHD** | 0,13(0,05)* | 0,07(0,04) | 0(0,07) | -0,1(0,05)* |
| **Dev dis** | 0,08(0,05) | 0,08(0,04) | 0,02(0,07) | -0,07(0,05) |
| Dyscalculia | 0,14(0,07) | 0,1(0,06) | -0,06(0,11) | -0,15(0,07)* |
| Language dis | 0,08(0,08) | 0,09(0,06) | 0,17(0,12) | -0,15(0,08) |
| **Adult dis** | 0,01(0,04) | 0(0,03) | 0,19(0,05)* | -0,04(0,04) |
| Depression | 0,02(0,05) | -0,01(0,04) | 0,14(0,07)* | 0(0,05) |
| Anxiety | -0,06(0,05) | 0,06(0,04) | 0,2(0,06)* | -0,02(0,04) |
| OCD | 0,01(0,08) | -0,07(0,08) | 0,39(0,13)* | -0,1(0,08) |
| Subst. Abuse | -0,04(0,09) | 0,02(0,07) | 0,24(0,13) | 0,03(0,1) |

### O0015

### Neurocognition and social cognition in delusional disorder: A systematic review

A. González-Rodríguez*, A. Álvarez Pedrero, A. Guàrdia Delgado, N. Sanz, S. Acebillo, J.A. Monreal, D. Palao Vidal and J. Labad Arias

Spain

**Introduction:** Recent research has revealed that neurocognitive and social cognitive deficits are core features in patients with schizophrenia. However, the link between both branches of cognition remains still far from definitive in delusional disorder.
**Objectives:** Thus, the main goal of this systematic review was to explore non-social and social cognitive deficits in patients with delusional disorder.
**Methods:** In accordance with the PRISMA statement, a systematic computerized search was performed using Pubmed and Scopus databases from inception until 4 June 2019. Search terms: ("Social cogn*" OR "cognition" OR "cognitive" OR "neurocognition" OR "neurocognitive" OR "neuropsychological" OR "metacognitive") AND ("delusional disorder" OR "paranoia"). Inclusion criteria: a) delusional disorder (ICD or DSM) and b) studies assessing neurocognition or social cognition in delusional disorder.
**Results:** A total of 2955 records were retrieved (Pubmed: 575; Scopus: 2380). After the screening and selection processes, 21 studies were eligible for inclusion: neurocognition (n=13), social cognition (n=6) and both (n=2). Studies reporting neurocognitive deficits: speed of processing (n=2), attention/vigilance (n=2), working memory (n=5), verbal learning (n=5), visual learning (n=2) and reasoning/problem solving (n=4). Studies reporting social cognitive deficits: emotion recognition (n=2), theory of mind (n=3), attributional style (n=5) and cognitive biases (n=3). Delusional disorder patients made more perseverative errors than healthy controls and showed lower deficits compared with schizophrenia.
**Conclusions:** This systematic review supports the notion that patients with delusional disorder are not cognitively unimpaired. Non-social and social cognitive deficits may be core features of delusional disorder. Dysfunctions in prefrontal and temporal brain regions may be implicated.
**Disclosure:** Alexandre González-Rodríguez has received honoraria and/or travel costs from Janssen and Lundbeck-Otsuka.

### O0019

### A network model of the relationship between loneliness, paranoia, and emotional disturbances

A.K.C. Chau* and S.H.-W. So

Hong Kong PRC

**Introduction:** Chronic loneliness has been associated with emotional disorders such as major depressive disorder and social anxiety disorder. More recently, loneliness is proposed to drive and maintain paranoia. Since emotional disturbances like depression and social anxiety often co-occur with paranoia, it is questionable whether loneliness is uniquely associated with paranoia after controlling for these emotional experiences. Besides, studying loneliness before onset of clinical psychosis will shed light on the possible role of loneliness as a contributor to paranoia rather than a consequence of the debilitating symptom.
**Objectives:** Using a stepwise network analytic approach, we modeled the unique associations between loneliness, paranoia, depression and social anxiety in a representative non-clinical sample. The moderating role of schizotypy in these associations was also explored.

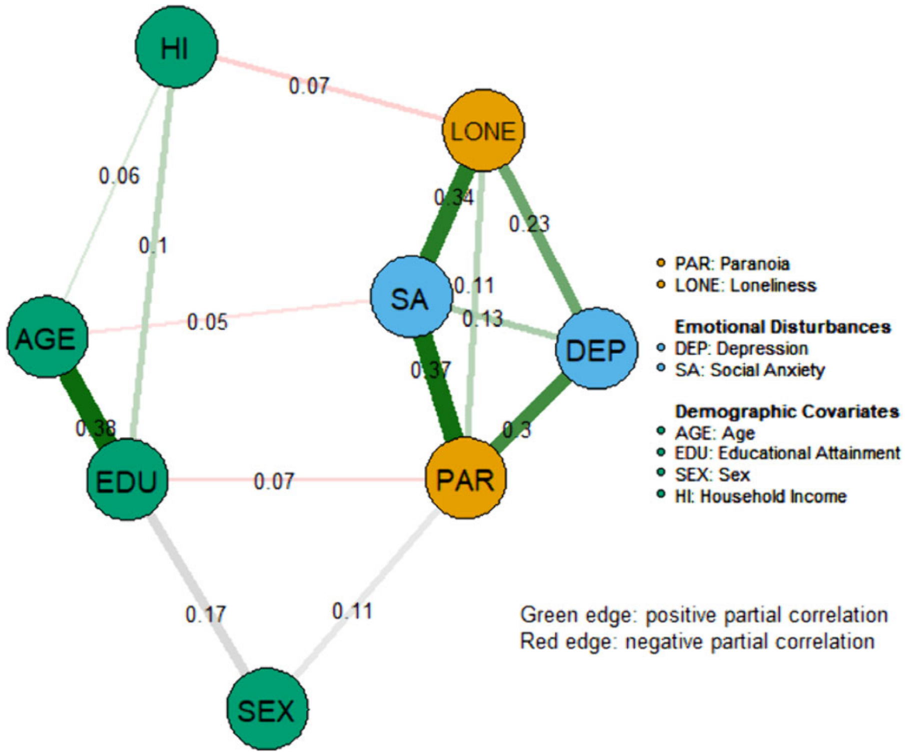

**Methods:** Young adults (age 18-30) were recruited via multi-site visits, advertising in public places, and social media, etc. Mixed graphical models with loneliness, paranoia, depression and social anxiety as nodes were estimated without, and then with demographic covariates. Edges were estimated as unique associations between nodes. The effect of schizotypy on these associations was tested with a moderated network model.

**Results:** The validated sample consisted of 1,935 participants. Loneliness was linked to paranoia directly and via depression and social anxiety indirectly. These edges remained robust after controlling for demographic covariates (Fig. 1). The association between loneliness and paranoia became weaker as level of schizotypy increased.

**Conclusions:** Loneliness and paranoia are associated directly and indirectly via emotional disturbances. There is a potential moderating role of schizotypy in the association between loneliness and paranoia.

**Conflict of interest:** No

### O0020

### Self- and caregiver-reported disability after one year of LAI antipsychotic treatment in schizophrenia: Preliminary results

G. D'Anna*, L. Tatini, V. Ricca and A. Ballerini

Italy

**Introduction:** Disability associated with schizophrenia constitutes a substantial obstacle to recovery. Over the years, SGA-LAI (Second Generation Antipsychotics, Long-Acting Injectable) have proved effective with regard to various clinical and patient-reported outcomes.

**Table 1** Sociodemographic and clinical data

| | |
|---|---|
| **Age (years)** | 38.63±13.97 |
| **Gender (M)** | 17 (56.7%) |
| **Education (years)** | 13.07±3.32 |
| **Illness duration (years)** | 13.86±10.34 |
| **Relapses** | 2.82±1.91 |
| **Hospitalisations** | 2.00±2.11 |

**Objectives:** To evaluate self- and caregiver-reported disability in 30 patients with schizophrenia before (T0) and one year after switching from an oral SGA to the corresponding LAI (T1).

**Methods:** Sample characteristics at baseline are summarised in Table 1. At T0 and T1, patients were assessed with Clinical Global Impression – Severity (CGI-S) and Positive And Negative Symptoms Scale (PANSS), and both the patient and a caregiver answered the 12-item WHO Disability Assessment Schedule 2.0 (WHODAS 2.0). Student's t test for longitudinal comparison was performed. Pearson's correlation was used to evaluate the overlap of proxy- and self-administered WHODAS 2.0 at each assessment.

**Results:** CGI-S and each subscale of PANSS (excluding negative symptoms) improved, and a significant reduction in disability was seen in the overall score of proxy- and self- administered WHODAS 2.0 (Figure 1), as well as in the number of days with functional limitation during the previous month (data not shown). Pearson's correlation between proxy- and self-administered WHODAS 2.0 confirmed a substantial overlap between the reports at each time (data not shown).

**Conclusions:** We observed a significant reduction in patients' disability, together with a psychopathological improvement. The overlap between caregiver and patient report underlines the consistency of this finding.

**Conflict of interest:** No

### O0022

### Variation of negative symptoms in people with schizophrenia after adding aripiprazole to the treatment with risperidone or paliperidone monotherapy

A. Arnaiz, O. Olivas, L. Erkoreka*, A. Arrue, A. Barreiro, N. Basterreche, M.I. Zamalloa and M. Zumarraga

Spain

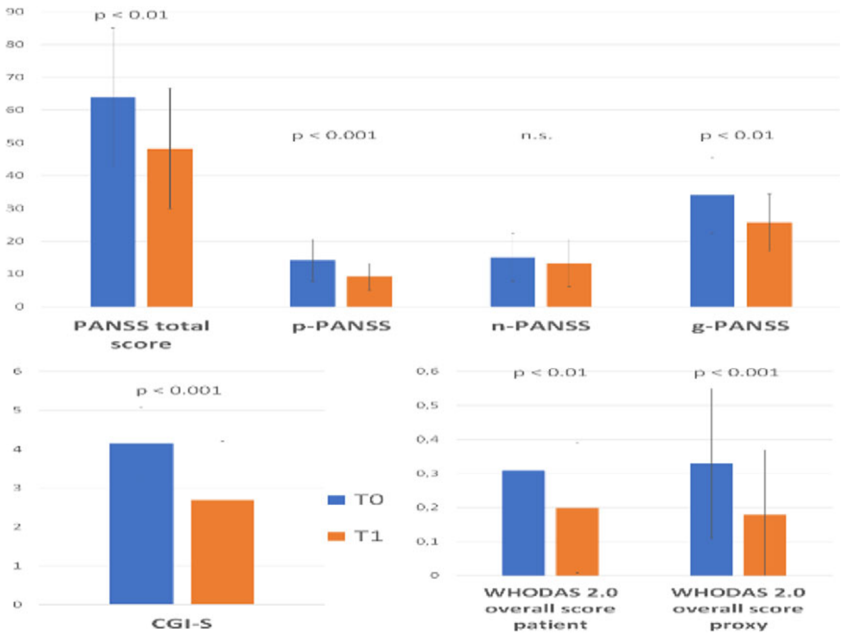

Table 1

| | Day0 | Day28 | % Day0 to day 28 | Improve n | No change n | Worsen n | Factors |
|---|---|---|---|---|---|---|---|
| Affective flatening | 3.1±1.3 | 2.2±1.4 | 30.7±32.2 | 39 | 24 | 1 | |
| Alogia | 2.5±1.6 | 2.0±1.4 | 17.9±29.3 | 25 | 38 | 1 | R>P Months on R/P |
| Avolition | 3.0±1.3 | 2.3±1.4 | 22.8±35.3 | 31 | 32 | 1 | ♀>♂ R>P |
| Anhedonia | 3.3±1.4 | 2.7±1.4 | 16.5±24.0 | 30 | 33 | 1 | ♀>♂ |
| Attentional impairment | 2.7±1.4 | 2.1±1.3 | 20.3±29.0 | 27 | 37 | 1 | Months on R/P |
| SANS total | 14.5±5.5 | 11.2±5.5 | 24.1±25.9 | 49 | 14 | 2 | ♀>♂ Months on R/P |

**Introduction:** The available medical literature reports the difficulty of treating primary negative symptoms (NS) as opposed to the rest of symptomatology. Aripiprazole (ARI) has shown to be effective in the treatment of NS

**Objectives:** Asses NS by means of SANS in patients treated with risperidone/paliperidone (R/P) monotherapy, before and after adjunctive ARI administration

**Methods:** Seventy four schizophrenic patients, 20 women and 54 men, mean age (DS) 44.47 (11.47) were selected. The SANS scale was applied before and after 28 days of adjunctive treatment with 5mg/day of ARI

**Results:** Table 1 shows the scoring of SANS symptoms, the percentage change (mean and SD) after 28 days, the factors influencing this variation and the number (n) of patients for which the scoring decreases, doesn't change or increases. All NS improve in a statistically meaningful way

**Conclusions:** Although literature indicates that core NS do not improve with antipsychotic therapy, they significantly do in our sample. This might be due to the action of ARI as a partial D2-agonist that might balance the hypothetic dopaminergic hypofunction at a mesocortical level, related to this symptomatology. The following limitations may affect the results: the investigator bias and the different timing in the previous treatment with R/P The current scales do not seem to reflect the conceptualization of NS, reason why it is necessary to redefine them and to create new scales

### ORAL COMMUNICATION SESSION 03: GENETICS & MOLECULAR NEUROBIOLOGY - PART I / NEUROIMAGING / NEUROSCIENCE IN PSYCHIATRY

#### O0023

#### Pharmacodynamic analysis of polymorphisms of DRD2 and HTR1A genes in antischizophrenic drugs

Y. Zhang* and X. Cui

China

**Introduction:** Gene detection is of great significance in guiding clinical medication. To investigate the effect of different genotypes of dopamine D2 receptor (DRD2) and 5-hydroxytryptamine receptor (HTR1A) on the actual therapeutic effect of schizophrenia patients, and the distribution characteristics of receptor genotypes.

**Objectives:** investigate the effect of different genotypes of DRD2 and HTR1A.

**Methods:** 239 schizophrenic patients were collected. The therapeutic effect of the drugs was evaluated by PANSS score reduction rate of 30 (+10) days. The results were compared with those reported in the gene report. The aggregated data were analyzed by Excel, and different response of effective genotype receptors to drugs in clinical practice were obtained.

**Results:** There was no statistical difference between the sexes, age and the number of effective cases in different drugs treat. DRD2 and HTR1A genetic polymorphismsm have nine combinations.The effective rate of antischizophrenic drugs in various genotype combinations was about 60%. When the DRD2 genotype was the same, the efficiency of aripiprazole and amisulpride increased with the increase of HTR1A G in the genotype, while olanzapine and risperidone decreased with the increase of G, quetiapine was consistent. Olanzapine, aripiprazole and risperidone may have better efficacy in HTR1A genotype CC, and amisulfapride and quetiapine may have better efficacy in GG or GC.

**Conclusions:** Antischizophrenic drugs have different therapeutic effects on DRD2 and HTR1A, and are also no affected by sex, age and drug use. The drug efficacy of DRD2 genotype Del/Del, Int/Del is higher than Ins/Ins. The drug efficacy of HTR1A is affected to the effect of DRD2 gene.

#### O0024

#### An in-depth analysis of DNA methylation in three genetic LOCI shared between schizophrenia and intelligence

M. Alfimova, N. Kondratyev, A. Golov, T. Lezheiko* and V. Golimbet

Russian Federation

**Introduction:** The molecular mechanisms of cognitive deficit in schizophrenia are poorly understood. They can be investigated by studying the functional consequences of genetic variants associated with both schizophrenia and cognition in whole-genome association studies (GWAS). Of particular interest is DNA methylation within such shared loci, as it can mediate the influence of genetic factors on schizophrenia risk and symptoms through the regulation of gene expression.

**Objectives:** To perform a detailed analysis of methylation of three DNA fragments of about 1000 bp in length within the MIR137HG, BAG5 and SLC39A8 loci, which are common for the largest schizophrenia and intelligence GWASs, using peripheral blood of 74 schizophrenia patients and 68 healthy controls.

**Methods:** The methylation level of each cytosine in both the CpG and non-CpG contexts was assessed by single molecule real-time bisulfite sequencing and analyzed for the relationship with haplotypes, schizophrenia, and cognitive functioning.
**Results:** The majority of cytosines were hypo- or hypermethylated. The intermediately methylated sites were rare and mostly represented by CpG-SNPs. These sites showing allele-specific methylation were not associated with schizophrenia or cognition. At the same time, in the MIR137HG locus, we found an intermediately methylated CpG, which was not related to the local common polymorphism. Its methylation was higher in schizophrenia men compared to controls and correlated negatively with cognitive functioning. The adjacent CpH was also variably methylated.
**Conclusions:** Thus, this CpG might represent a biomarker of cognitive deficits in schizophrenia as well as an epigenetic mark of environmental influences. This work was supported by the Russian Scientific Foundation, grant 16-15-00056.

### O0025

#### The study of effects of oxytocin pathway genes and early-life stressors on social functioning in schizophrenia

T. Lezheiko*, S. Smirnova, V. Mikhailova, E. Krikova, N. Kolesina, A. Asanov and V. Golimbet
Russian Federation

**Introduction:** The deterioration of social functioning is considered not only a concomitant symptom of schizophrenia, but is one of the central characteristics of the disease. The neurohormone oxytocin plays a key role in social cognition and social behaviors, which are important in disorders with impaired social functioning. It has also been shown to modulate reactivity to social stressors. There is some evidence that genetic polymorphisms in oxytocin pathway genes are associated with social dysfunction in schizophrenia.
**Objectives:** To search for the association between CD38 (rs3796863), OXTR (rs53576) , OXTR (rs4686302) and social functioning in patients with schizophrenia and to assess if the association is moderated by early-life stressors.
**Methods:** Social functioning was assessed with the Personal and Social Performance (PSP) scale in 713 patients (women 47%), aged from 16 to 70 years, with ICD-10 diagnosis of schizophrenia or schizoaffective psychosis. Patients were stratified by the level of social functioning into two groups: “moderate and good” and “marked and poor”. Allele and genotype frequencies were calculated in each group. Alcoholism in the family (n=120) and growing up in a single parent family (n=209) were studied as stressors of early-life.
**Results:** The early-life stressors exert no significant effects on social functioning. The frequency of the GG (rs53576) genotype was significantly higher (p=0.013; 95%CI 1.6 (1.1-2.3)) in the “marked and poor” group compared to the “moderate and good” group.
**Conclusions:** The OXTR (rs53576) gene, reported earlier to be associated with social behavior, is associated with social functioning in schizophrenia. This work was supported by RFBR grant N 17-29-02088.

### O0031

#### Intracranial volume and subcortical structures in adolescents with early-onset psychosis: A mega-analysis from the enigma early-onset psychosis working group

I. Agartz[1]*, T. Gurholt[1], S. Nerland[1], P. Thompson[2] and O. Andreassen[1]
[1]Norway and [2]United States of America

**Introduction:** Adolescent early-onset psychosis disorders (EOP) are rare and heterogeneous mental disorders with unknown causes and suboptimal treatment. The EOP phenotype can provide novel insight to the biological brain mechanisms of psychosis and be a predictive marker of future brain development and clinical outcome.
**Objectives:** To determine volumetric case-control differences in subcortical brain structures and intracranial volume and clarify subgroup differences and medication effects using the hitherto largest neuroimaging sample of EOP patients and typically developing controls from the ENIGMA Early Onset Psychosis (EOP) Working Group.
**Methods:** T1-weighted MRI scans from 296 EOP patients (age 16.5±1.4 years; illness duration 1.6±1.5 years; 40.5% girls; N EOS=183, Affective psychosis=72, Other psychosis=41) and 360 healthy controls (age 15.9±1.7 years; 45.6% girls) were pooled from 11 cohorts worldwide and processed in FreeSurfer following the standardized ENIGMA pipeline. Subcortical volumes (both hemispheres combined) and ICV were extracted. Group differences were analyzed by linear mixed-effects model adjusting for age, sex and ICV, with scanner as random variable. False discovery rate was applied for multiple comparisons.
**Results:** EOP patients showed significantly smaller intracranial (Cohen’s d=-0.36) and hippocampal (d=-0.25) volumes, and larger caudate (d=0.24) and lateral ventricular (d=0.23) volumes than controls. Early-onset schizophrenia patients had smaller intracranial (d=-0.31) and larger pallidum (d=0.29) volumes. Medicated patients had smaller intracranial volume (d=-0.36).
**Conclusions:** The pattern of brain differences in EOP is similar as demonstrated in adult psychosis but show smaller intracranial volumes. EOP, particularly early-onset schizophrenia, may represent a distinct and more severe phenotype closer to the neurodevelopmental origins of psychosis pathology.

### O0032

#### Resting-state FMRI correlates of clinical response to stimulant treatments in children and adolescents with ADHD

V. Pereira-Sanchez[1]*, A. Franco[1], P. De Castro-Manglano[2], M. Vallejo-Valdivielso[2], A. Diez-Suarez[1,2], M. Fernández-Seara[2], M. Fernandez-Martinez[2], M. Milham[1] and F. Castellanos[1]
[1]United States of America and [2]Spain

**Introduction:** Attention-deficit/hyperactivity disorder (ADHD) is a common neurodevelopmental condition in which symptoms respond to several pharmacological treatments. However, little is known about the brain mechanism of action of those treatments and the heterogeneity of response across different patients.
**Objectives:** To determine the resting-state fMRI correlates of treatment response to stimulants in ADHD children.
**Methods:** We recruited 68 boys and girls (ages 7-17) with ADHD in an outpatient setting. Sociodemographic, neuropsychological, and clinical data and blood samples were collected from participants, who also underwent neuroimaging (resting-state fMRI scan). Neuroimaging data were preprocessed (including motion correction) and (fractional) amplitude of low-frequency fluctuations ((f)ALFF) metrics were extracted with CPAC v1.4.3 software. All data passed quality control assessments. Initial comparison between 38 patients treated with stimulants and 23 treatment-naïve patients was done with AFNI 3dttest++. (f)ALFF were also compared in a subsample (n=8) of naïve subjects pre- vs. post-acute treatment with methylphenidate, all of whom had good clinical responses.
**Results:** Initial cross-sectional between-group comparisons of (f) ALFF yielded no statistically significant results. In the pre-post within-subject comparison, methylphenidate induced a significant increase of fALFF in a cluster in the right primary visual cortex (MNI coordinates 12,-78,12, corrected p<0.05).
**Conclusions:** Our findings suggest a modulatory effect of intrinsic BOLD signal fluctuations in the primary visual cortex by methylphenidate treatment in a small sample of clinically-responsive ADHD children and adolescents. Our preliminary results illustrate the improved statistical power of within-subject analyses, and highlight the primary visual cortex as a potentially relevant locus in the pharmacodynamics of ADHD treatment.

### O0033

#### Dysfunctional brain activity and the inability to delay gratification in obesity with or without binge eating disorder

R. Miranda-Olivos[1*], T. Steward[2], I. Martínez-Zalacaín[1], G. Testa[1], S. Jimenez-Murcia[1], C. Soriano-Mas[1] and F. Fernandez-Aranda[1]
[1]Spain and [2]Australia

**Introduction:** The inability to delay gratification, in some individuals with obesity (OB) and the eating disorder, could be associated with greater impulsivity to immediate rewards. Dysfunctional brain activity involves corticostriatal circuits related to motivation and impulse control. In obesity with or without binge eating disorder (BED) immediate rewards choices can impede to self-regulate food intake and a diminished success with weight loss in obesity treatment.
**Objectives:** We examined differences of delay gratification between individuals with obesity and healthy-weight and we explore these differences associated with neural activity.
**Methods:** Seventy adult women were recruited and classified by Body Mass Index (BMI) between healthy weight (HW-group) (N=33; BMI= 18-24.99) and OB-group (N=37; BMI= 30-40) with or without BED. All participants were underwent to functional magnetic resonance session, where a well-established monetary delay discounting task was implemented (5). Overall and three sizes of delay and reward (small, medium and large) scores were obtained and will be used in neuroimaging analyses at the single-subject level and subsequently between-group comparisons.
**Results:** Preliminary behavioral results OB-group (included BED) prefers a smaller-sooner reward comparison to HW women in scores: overall (T=-2.283; p-value=0.026), small (T=-2.067; p=0.043) medium (T=-2.158;p=0.033), but not in delayed and reward of large size (T=-1.999; p=0.050). Neural correlates results could support differences detected in delay discounting scores providing a better understanding of dysfunctional brain activity in obesity.
**Conclusions:** The preference for smaller-sooner rewards may explain dysregulated eating in obese condition. Behavioral interventions based on the identified functional activity could optimize weight-loss strategies and improve eating behavior in this condition.

## ORAL COMMUNICATION SESSION 04: CHILD AND ADOLESCENT PSYCHIATRY - PART I

### O0035

#### Tunisian validation of a cyberbullying assessment instrument: The « second revision of the revised cyberbullying inventory »

L. Sahli, S. Bourgou*, S. Haj Amor, R. Fakhfakh and A. Belhadj
Tunisia

**Introduction:** Cyberbullying is a form of peer violence that became a widespread problem. The prevalence of cyberbullying is unknown in Tunisia due to the absence of validated assessment instruments.
**Objectives:** The aim of this study was to validate the questionnaire « Second Revision of the Revised Cyberbullying Inventory (RCBI-II) ».
**Methods:** We translated the RCBI-II into dialectal Tunisian Arabic based on the translation back-translation method. To study construct validity and reliability, we conducted a cross-sectional study involving 962 adolescents. Confirmatory factor analysis was performed to study construct validity for the two dimensions of the scale: cyber-victimization and cyber-aggression. To test reliability, the global internal consistency was computed for the two dimensions of the scale.
**Results:** The translated version was considered satisfactory. The adjustment indices of the confirmatory factor analysis were satisfactory for both sections. The values of cyber-aggression section were as follows: Comparative Fit Index (CFI)= 0.92; Tucker-Lewis Index (TLI)= 0.9; Root Mean Square Error Of Approximation (RMSEA) = 0.04; Standardized Root Mean Square Residual (SRMSR)= 0.01. As for cyber-victimization section, fit indices were as follows: CFI= 0.92; TLI= 0.9; RMSEA= 0.01; SRMSR= 0.07. The two sections showed good reliability. The internal consistency of each section was optimal. Cronbach alpha was respectively 0.79 for cyber-aggression and 0.73 for cyber-victimization.
**Conclusions:** The Tunisian version of the RCBI-II is a psychometrically valid measure. It could be useful to conduct researches in Tunisia aimed to prevent cyberbullying.

### O0036

#### A controlled pharmaco-imaging trial of memantine for the treatment of social deficits in adolescents with high-functioning autism spectrum disorder

G. Joshi*

United States of America

**Introduction:** Multiple studies suggest glutamate dysregulation in Autism Spectrum Disorder (ASD). Proton magnetic resonance spectroscopic ($^{1}$HMRS) studies in ASD demonstrate abnormally high concentrations of glutamate (Glu) in the Dorsal Anterior Cingulate Cortex (dACC). Memantine, a glutamate antagonist, may improve behavioral and cognitive functioning in ASD.
**Objectives:** We sought to determine the response of social impairment to memantine treatment in adolescents with ASD and to explore spectroscopic dACC Glu activity as a biomarker of response to memantine therapy.
**Methods:** 40 adolescents (ages 13-17 years) with ASD participated in a 12-week, double-blind, placebo-controlled trial of memantine. Treatment Responders were defined as those with ≥ 25% reduction in informant-rated SRS-2 score and clinician-rated improvement subscale of the CGI score of ≤ 2. Neuroimaging data was acquired from ASD participants before and after the trial and from age-, sex-, and IQ-matched healthy controls.
**Results:** Forty adolescents with ASD were exposed in the study (memantine=19/placebo=21). There were significantly higher rates of treatment responders with memantine treatment vs. placebo. High Glu activity was observed in 61% of the participating ASD population. Memantine response was significantly greater than placebo in ASD participants with High-Glu activity. Furthermore, the response to memantine in ASD was significantly higher in the presence of High versus Normal Glu activity.
**Conclusions:** The glutamate modulating agent memantine was well tolerated and significantly effective in treating social deficits in adolescents with HF-ASD. The atypically high dACC Glu activity observed in more than half of the ASD study population with ASD served as a predictor of response to memantine therapy.
**Disclosure:** Dr. Gagan Joshi is supported by the National Institute of Mental Health (NIMH) of the National Institutes of Health (NIH) under Award Number K23MH100450. He receives research support from Pfizer, Demarest Lloyd, Jr. Foundation, and Simons Center for the S

**Keywords:** Memantine; Autism; Pharmaco-Imaging

### O0037

#### Externalising psychopathology and suicidal behavior during adolescence: A 17 years population based study

A. Forte[1]*, M. Orri[2], M. Pompili[1], C. Galera[3], R. Trambley[2] and S. Côté[2]

[1]Italy; [2]Canada and [3]France

**Introduction:** Externalising symptoms in childhood, such as Hyperactive/inattentive symptoms (ADHD symptoms) or Irritability, are associated with adolescent suicidal behavior in clinical studies, but there is still a lack of population-based longitudinal investigations on the developmental aspects of this association. Additionally, it is unclear whether the association is similar for boys and girls.
**Objectives:** The objectives of the study were to test the association between ADHD during childhood and suicidal ideation and attempt during adolescence, and to investigate sex differences.
**Methods:** 1407 children from the Québec Longitudinal Study of Child Development were followed up from 5 months to 17 years of age. We used teacher-reports of ADHD symptoms from 6 to 12 years, and self-report of suicidal ideation and attempt at 13, 15, and 17 years. We identified three ADHD symptoms trajectories: low (boys: 32.2%, girls: 48.7%), moderate (boys: 44.6%; girls: 42.2%) and high (boys: 23.2%; girls: 9.1%).
**Results:** Compared to boys on a low trajectory, boys on a moderate trajectory were at higher risk for suicidal ideation (OR 4.2, 95% CI 1.2–14.8), and boys on a high trajectory were at higher risk for suicide attempts (OR 4.5, 95% CI 1.1–17.9). Girls on moderate or high ADHD symptoms trajectories were not at higher risk for suicidal ideation or attempts than girls on low trajectories.
**Conclusions:** For boys, but not for girls, moderate-to-high ADHD symptoms increased the suicidal risk in adolescence. Interventions with boys showing ADHD symptoms should include a suicide prevention component.

### O0040

#### Influence of 3-omega fatty acids (FA) on clinical status and inflammatory markers in depressive disorder in children

J. Trebaticka[1]*, A. Surovcova[1], Z. Matzova[1], Z. Hradečna[1], M. Vavakova[1], I. Garaiova[2], J. Šuba[1] and Z. Ďuračkova[1]

[1]Slovak Republic and [2]United Kingdom

**Introduction:** The relationship between the inflammatory agents found in depressive disorder (DD) and physical health indicate that omega-3 FA intake may be a promising therapeutic modality.
**Objectives:** The aim of this pilot, single-centre, randomized, double-blind controlled study was to investigate the levels of inflammatory markers, thromboxanes in plasma and urine in children with DD and in controls before fish oil emulsion administration and influence omega 3-FA intake on clinical status of depression.
**Methods:** We randomised 41 children (11 to 17 years) to the Omega-3 FA or Omega-6 FA groups. Patients were clinically investigated with CDI score (Children's Depression Inventory). The level of thromboxane B2 in plasma was determined by ELISA kit and in urine as 11-dehydrothromboxane B2 by ELISA Kit-Monoclonal, 8-isoprostanes by kit Cayman.
**Results:** CDI scores decreased after the omega-3 FA supplementation by 27% compared to omega-6 FA (13%). In patients with DD, we found increased basal plasma TXB2 level compared to the control group ($p = 0.001$). Omega-3 FA decreased level of thromboxanes in plasma after 6 and 12 weeks about 27% ($p= 0.044$, $p=0.014$, resp.) in the contrary to omega-6 FA, which had no effect on TXB2 levels ($p = 0.693$, $p = 0.150$, resp.).
**Conclusions:** The omega-3 FA could be a suitable adjuvant therapy for reducing the severity of depressive symptoms and inflammation in depressive children and adolescent. This study was supported by the APVV grant 15-0063 and Mind and Health, civil association.

### O0041

### Radical thinking predicts socially desirable appraisals of self-regulation in adolescents with delinquent behavior

A. Tkhostov*, E. Rasskazova, L. Falkovskaia, A. Kiseleva and V. Emelin

Russian Federation

**Introduction:** Previous study revealed that adolescents with drug addictions tend to give extreme answers to some general items (Rasskazova et al., 2019) that allowed to create "black-and-white" thinking scale measuring tendency to radical thinking.
**Objectives:** The aim was to study "black-and-white" thinking in adolescents in delinquent behavior.
**Methods:** 760 adolescents 13-17 years old from three general schools and 288 adolescents from eight special education institutions for adolescents with delinquent behavior (most with F91) filled Psychological Risk Factors of Deviant Behavior in Adolescents Inventory (Rasskazova et al., 2019) and Hospital Anxiety and Depression Scale (Zigmond, Snaith, 1983).
**Results:** There were no differences in "black-and-white" thinking scale in adolescents with and without delinquent behavior. In both control and clinical groups "black-and-white" thinking was related to social desirability scale (r=.26 and r=.36, p<.01) and, after adjusting for social desirability, predicted ($\Delta R^2$=2.3-3.4% and $\Delta R^2$=3.1-8.7%, p<.01) more favorable appraisals of lower antisocial tendencies (β=-.18 and β=-.30), technological addiction tendencies (β=-.19 and β=-.24), higher self-regulation (β=-.16 and β=-.19), lower risky behavior (β=-.16 and β=-.18). In clinical group but not in controls "black-and-white" thinking also was related to higher depression (β=.14, p<.05, $\Delta R^2$=1.9%).
**Conclusions:** Although severe Internet misuse is obviously related to wide range of psychopathology, normative perception of danger in the Internet could affect specific anxiety in youth but suppress anger and hostility in youth and adults. Study is supported by the Russian Science Foundation, project 18-18-00365.
**Disclosure:** Research is supported by the Russian Foundation for Basic Research, project No. 18-013-01222.

### O0043

### Online multitasking in adolescents: Important skill or sign of internet addiction?

G. Soldatova, E. Rasskazova, D. Vinitskiy* and S. Chigarkova

Russian Federation

**Introduction:** Internet opens opportunities and demands from children and adolescents involvement and switching between different activities. While multitasking is typically defined as favorable skill to simultaneously regulate different activities (Lee and Taatgen, 2002, Oberauer and Kliegl, 2004), in adults it could lead to poorer performance (Furnham, Bradley, 1997, Foerde et al., 2006). In adolescents it could be result of better digital skills or indicator of regulatory difficulties or Internet addiction.
**Objectives:** The aim was to study relationship between subjective multitasking in adolescents. digital competence and excessive Internet use.
**Methods:** 99 adolescents (53 males) 12-17 years old appraised (using Likert scale) how frequently they are doing several activities simultaneously and how successful they feel they are. Then they filled Excessive Internet Use Scale (Livingstone et al., 2012) and Index of Digital Competence (Soldatova, Rasskazova, 2014).
**Results:** 50.5% replied that they often or always are doing several activities and 32.3% felt that they are successful in any of the activities. There were no gender or age differences in frequency and satisfaction of multitask activities. Both excessive Internet use and digital competence (online skills) predicted frequency of multitask activities (β=.20 and β=.22, $R^2$=9.8%, p<.01) but not satisfaction with them.
**Conclusions:** Data suggests that frequency of multitasking online could be both related to better skills or excessive Internet use and should be further qualified by objective measures of effectivity. Study is supported by the Russian Foundation for Basic Research, project 19-29-14181mk.
**Disclosure:** Study is supported by the Russian Foundation for Basic Research, project 19-29-14181mk

### O0044

### Autism spectrum disorders in a population of very low birth weght: Causes and consequences

S. Uccella*, E. De Grandis, E. Dufour, D. Tortora, M. Severino, M. Malova, L. Nobili and L.A. Ramenghi

Italy

**Introduction:** Preterm birth has been recenlty associated to a wide range of neurodevelopmental sequelae, among them Autism Specturm Disorders (ASD) seem having a higher prevalence.
**Objectives:** To describe prevalence of ASD in a population of Very Low Birth Weight preterm babies (VLBW) and to investigate possible risk factors.
**Methods:** A retrospective studywas conducted on VLBW born between January'12 and April'16 at our Institutite. VLBW who underwent MRI at Term of Equivalent Age and a developmental evaluation at 3years were included. Patients carrying major brain lesion concerning prematurity and genetic/malformative disorder were excluded. Case group consisted of VLBWwho received diagnosis of ASD. VLBW scoring a Global Developmental Quotient (GDQ)>85 at Griffith's Mental Development Scale were considered as controls. For risk factor analysis obstetric and neonatal, MR and family history data were noted
**Results:** Among 289VLBW born in the selected period, 97were selected: 14VLBW received diagnosis ofASD and 83 were assigned to control group. Significant risk factors were male sex (78,6% vs37,3%; p=0,007), incomplete steroid prophylaxis (35,7% vs77,1%; p=0,003), caesarean section (35,7%vs85,5%; p<0,001) and positive family history of psychiatric disorders (57,1%

vs12,3%; p=0,001). Positive familiar history of immunological diseases (78,6%vs49,4%; p=0,08) and presence of a number>6 of PunctateWhiteMatterLesions(PMWL) (40%vs7,2%; p=0,06) were close to significance.

**Conclusions:** In this study, prevalence of ASD among VLBW was 4,9%.Data of significance of positive familiar history for psychiatric disease combined to close value to significance for positive familiar history for immunological diseases and presence of a number>6 ofPMWL could suggest an immunopathological genesis of ASD. Anyway, studies on larger populations are needed.

**Conflict of interest:** No

### ORAL COMMUNICATION SESSION 05: CULTURAL PSYCHIATRY / MENTAL HEALTH CARE / TRAINING IN PSYCHIATRY / OTHERS

#### O0047

#### Patient-reported satisfaction in psychiatry admission in a tertiary-care teaching hospital in nepal: A descriptive cross-sectional study

S. Joshi*, T. Thakur and R. Shakya

Nepal

**Introduction:** Satisfaction among the patients reflects the quality of care provided. Patient demographics, diagnosis, and chronicity of illness, institutional and/or medical factors have been found as contributing factors in different studies.

**Objectives:** To evaluate the satisfaction of psychiatric inpatients in a tertiary care center.

**Methods:** This was a descriptive cross-sectional study on the inpatients at the Department of Psychiatry of a tertiary care university teaching hospital in Nepal during a one-year study period from May 2015 to June 2016. We interviewed the inpatients at the time of discharge and collected data on the demographic and clinical characteristics, the satisfaction of treatment received and sought any suggestions to improve the services. We summarized the numerical variables with the median and inter-quartile range (IQR) and the categorical variables as proportion.

**Results:** Of the 286 patients admitted during the study period, 122 patients responded (42.6%). The median age was 32 years (IQR 22-43) and the majority were male (73, 59.8%), married (78, 63.9%), skilled labor or student (26, 21.3%) by occupation and had secondary level education (31, 25.4%). The majority of the patients reported satisfaction (115, 94.2%) with the care received. Among the different domains of satisfaction, most positive responses were seen in the treatment (116, 95.08%) and the least in the treatment cost (57, 46.7%). Suggestions on the improvement of infrastructure of the ward and subsidy in the treatment cost were received.

**Conclusions:** The majority of the inpatients reported satisfaction with the care they received. Further exploration into different domains of satisfaction is needed.

**Conflict of interest:** No

#### O0048

#### Outcomes from the IBBIS randomized controlled trial (n=611): Integrated vocational rehabilitation and mental health care for people with anxiety and depression

A. Hoff*, J. Fisker, M. Nordentoft, R. Poulsen, C. Hjorthøj and L. Eplov

Denmark

**Introduction:** Anxiety and depression are very common causes of long-term sick leave. Different interventions are needed, but lack of coordination of these, seem to entail confusion among sick employees, afflicting their recovery.

**Objectives:** The aim of this study is to examine the efficacy of the IBBIS intervention: integrated vocational rehabilitation (VR) and mental health care (MHC), compared to non-integrated interventions.

**Methods:** 3-armed RCT. 611 Participants on sick leave due to depression or anxiety were randomized to one of three groups: 1, treatment as usual, (standard MHC and standard VR; 2, IBBIS MHC, consisting of cognitive behavioral therapy, and standard VR; 3, the IBBIS intervention: integrated MHC and VR. Primary outcome is time to stable return to work (RTW), measured at 12-month follow-up. Secondary outcomes are RTW at 6- and 24-month follow-up, and at 6-month follow-up: levels of depression, anxiety, perceived stress, and functional measures.

**Results:** Two of the groups seem to have a faster return to work than the third group (both HR~1.4, both p<0.036), as well as lower symptom levels. All secondary outcomes will be analyzed soon after abstract deadline.

**Conclusions:** From ongoing analyses, the currently emerging pattern shows two of the groups yielding consistent better results than the third, on both duration of sick leave and symptom levels. Seemingly, different organization and/or content of services matter: directions and magnitudes of all outcomes will be presented

**Conflict of interest:** No

#### O0049

#### Relationship between maternal mindfulness training during pregnancy and executive abilities in children

S. Kiselev*

Russian Federation

**Introduction:** It is known that maternal anxiety during pregnancy can affect child outcomes. We have shown that maternal mindfulness training during pregnancy has positive effect on the development of executive, language and memory abilities in 5–7 years old children (Kiselev, Volik, 2017).

**Objectives:** The goal of this study is to evaluate the effect of the maternal mindfulness training during pregnancy on executive abilities in 7–8 years old children.

**Methods:** In current study we included 24 women who participated in the maternal mindfulness training during pregnancy. The control group included 24 women who did not participate in this training during pregnancy. When the offspring of the target pregnancies were between 7 and 8 years of age, their executive abilities was assessed by 4 subtests from NEPSY (Tower, Auditory Attention and Response Set, Visual Attention, Statue).
**Results:** One-way ANOVA was used to reveal group differences in performing executive tasks. We have revealed the significant differences ($p<.05$) between groups in 3 subtests from NEPSY (Tower, Auditory Attention and Response Set, Visual Attention). The children from the experimental group had better executive abilities.
**Conclusions:** These results suggest that maternal mindfulness training during pregnancy may have positive effect on the development of executive abilities in 7–8 years old children. The research was supported by Act 211 Government of the Russian Federation, agreement 02.A03.21.0006.

### O0051

### Substance abuse in acute psychiatric unit inpatients

L. Procenesi, R. Colasuonno*, P. Brogna, A. Talamo, F. Di Michele, C. Niolu and A. Siracusano
Italy

**Introduction:** Correlations between psychiatric diseases and consumption of substances of abuse are common. Nevertheless, there are few data regarding this correlation in patients admitted to an acute psychiatric unit.
**Objectives:** The purpose of this survey is to detect the prevalence of substance abuse in a sample of acute psychiatric unit inpatients and to evaluate statistically significant differences, among some domains (restraint, involuntary commitment, violent acts, LAI therapy prescription), between abusers and non-abusers patients.
**Methods:** We included 542-inpatients (January 2017 -March 2019); 39,5% were abusers, 60,5% were non-abusers. The sample was divided into three age groups: 18-35 years, 36-50 years and over 50 years (img1).
**Results:** The percentage of restraint, involuntary commitment, violent acts and LAI therapy prescription were higher in the

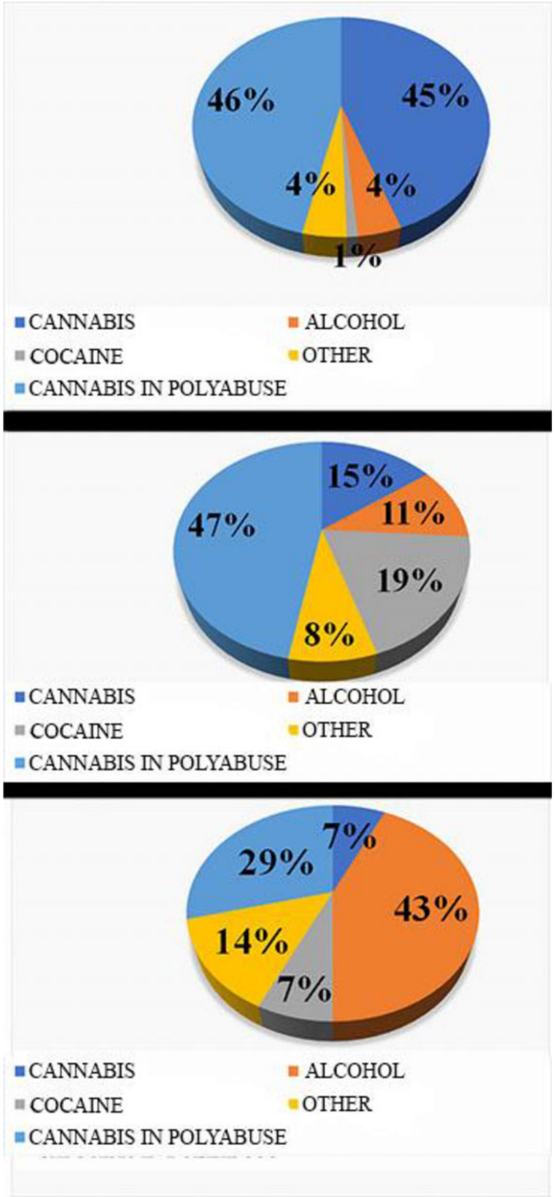

| | TOT | ABUSERS | %ABUSERS | NON-ABUSERS | %NON-ABUSERS |
|---|---|---|---|---|---|
| PATIENTS | 542 | 214 | 39% | 328 | 60% |
| F | 282 | 75 | 35% | 207 | 63% |
| M | 260 | 139 | 65% | 121 | 37% |
| MEAN AGE (yrs) | 41.9 | 36,5 | | 45,4 | |

| AGE RANGE | ABUSERS | %ABUSERS | NON-ABUSERS | %NON-ABUSERS | TOT |
|---|---|---|---|---|---|
| 18 - 35 years | 107 | 53% | 95 | 47% | 202 |
| 36 - 50 years | 79 | 43% | 106 | 57% | 185 |
| > 50 years | 28 | 18% | 127 | 82% | 155 |

abusers. The percentage of abusers was higher in the 18-35 years range (53%). In 18-35 years, abusers, the percentage of restraint, violent acts and LAI therapy prescription (48% are treated with LAI, 71% of which second generation LAI) were higher than in non-abusers. In 36-60 years, abusers, the percentage of restraint, violent acts, involuntary commitment and LAI therapy prescription (43%) were higher than non-abusers. There were not statistically significant differences between abusers and non-abusers in the over 50 years group. Concerning the type of substance abused, almost the totality of 18-35 years group of abusers used cannabis alone or in polyabuse (img2).
**Conclusions:** The Increasing use of substances is related to a worsening of the psychiatric disturbances. Our results suggest that considerable complexity in treatment management of these patients is required.

### O0052

#### Overview of the phenomenon of violence against psychiatric trainees in europe: The EFPT-VAPT study

V. Pereira-Sanchez*[,1], A. Gürcan[2], G. Erzin[2], Y. Rai[3], S. Gnanavel[3], A. Fontaine[4], J. Vieira[5], M. Asztalos[6] and A. Szczegielniak[7]
[1]United States of America,; [2]Turkey,; [3]United Kingdom,; [4]France,; [5]Portugal,; [6]Denmark, and [7]Poland

**Introduction:** Workplace violence suffered by psychiatric trainees from the patients they treat jeopardizes their safety and the quality of their care. This phenomenon has been often described in many countries but had not been so far systematically surveyed in Europe.
**Objectives:** The European Federation of Psychiatric Trainees (EFPT) -Research Working Group- Violence Against Psychiatric Trainees (VAPT) study aimed primarily at describing the extent and circumstances of the VAPT phenomenon in the European region (WHO).
**Methods:** An online questionnaire of 15 items was designed following pilot surveys in Spain, France, and Turkey, and distributed to psychiatric trainees in European countries via national coordinators between June-December 2018. The questions asked for anonymous demographic data as well as information about violent episodes experienced, consequences, and surrounding circumstances. Statistical descriptive analyses were conducted in SPSS. This study was approved by an Ethics Committee in Spain.
**Results:** 790 trainees from 38 European countries completed the survey (68.1% women and 80.1% trainees in adult psychiatry). 84.4% reported having ever been assaulted, most of them recently (77.5% verbally, 8.1% sexually and 47.7% physically). Most victims did not report the assault and experienced psychological consequences. Only a minority of respondents reported having proper preventive and management policies in their institutions.
**Conclusions:** VAPT phenomenon is commonly reported by trainees across Europe and frequently incur psychological damage. However, a majority of trainees reported poor institutional policies to prevent and manage these events. These results encourage local and international policymakers to effectively tackle this problem.

## ORAL COMMUNICATION SESSION 06: EMERGENCY PSYCHIATRY / PSYCHOPHARMACOLOGY AND PHARMACOECONOMICS / PSYCHOSURGERY & STIMULATION METHODS (ECT, TMS, VNS, DBS) / REHABILITATION AND PSYCHOEDUCATION

### O0057

#### Trends in prevalence and mean doses of commonly used antipsychotic drugs in scandinavia: A multi-national drug utilization study

M. Højlund[1*], A. Pottegård[1], E. Johnsen[2], R. Kroken[2], J. Reutfors[3], P. Munk-Jørgensen[1] and C. Correll[4]
[1]Denmark,; [2]Norway,; [3]Sweden, and [4]United States of America

**Introduction:** Use of antipsychotic drugs is common in non-psychotic disorders such as depression, anxiety, autism spectrum disorders, and insomnia. But exposure to antipsychotic drugs has been associated with a range of serious adverse events from arrythmias to diabetes. There is a need for detailed information on current practice and trends in the utilization of this drug class to guide future initiatives on the rational use of antipsychotic drugs.
**Objectives:** To investigate trends in dosing and prevalence of antipsychotic prescriptions in Scandinavia.
**Methods:** We retrieved data on antipsychotic use between 2006 and 2016 from Danish, Norwegian and Swedish national prescription registers. For each antipsychotic, we calculated prevalence of use and mean doses, overall and for specific age groups.
**Results:** Antipsychotic use in Scandinavia increased from 16.5 to 17.2 users/1,000 inhabitants between 2006 and 2016 (+2.4%, p=0.02). In 2006, chlorprothixene and levomepromazine were the most commonly used antipsychotics. By 2016, quetiapine was the most used antipsychotic in all three countries and across all age groups, with an overall one-year prevalence of 4.05 to 9.97 users/1,000 inhabitants. Mean doses of quetiapine decreased markedly during the 11-year study period (0.46 to 0.28 DDD/user/day), while mean doses for clozapine and olanzapine remained high (0.90-1.07 resp. 0.66-0.88DDD/user/day).
**Conclusions:** We found an increasing prevalence of antipsychotic prescriptions that coincides with low and/or decreasing mean doses of the majority of commonly used antipsychotics in Scandinavia. Of all antipsychotics, this development was most pronounced for quetiapine. Reasons for and consequences of increased antipsychotic use that lasts shorter periods of time requires further study.

### O0060

#### Metabolic and cardiovascular effects of antipsychotic drugs. A meta-analysis of randomized controlled trials

E. Cassioli*, E. Calderani, L. Lazzeretti, B. Ragghianti, V. Ricca, E. Mannucci and F. Rotella
Italy

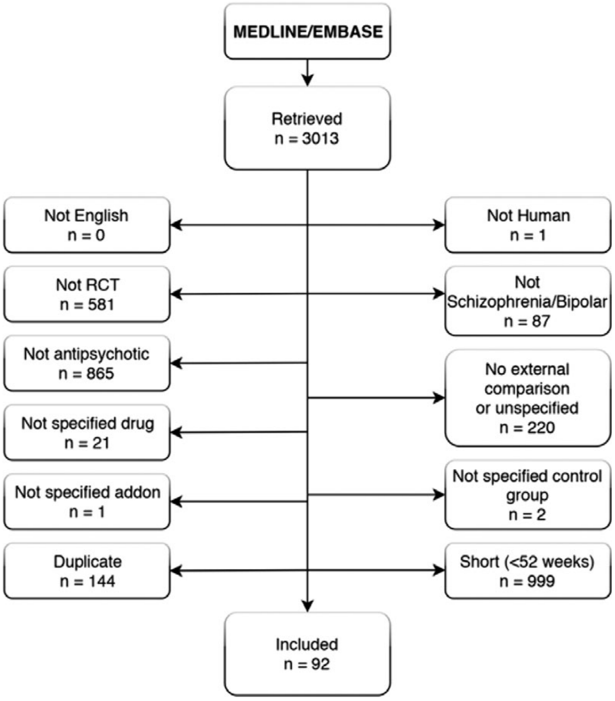

**Introduction:** The majority of randomized controlled trials (RCTs) on antipsychotics (APs) has efficacy as primary endpoint and provides a limited follow-up period. The lack of conclusive evidence on long term metabolic effects of antipsychotics has been reported as an issue demanding a more in-depth investigation.
**Objectives:** Aim of the present meta-analysis is to compare different APs for the long-term modification of risk of major adverse cardiovascular events and related mortality, in patients with schizophrenia and bipolar disorder.
**Methods:** All RCTs found on Medline/Embase of at least 52 weeks up to 19 December 2017, enrolling patients with bipolar disorder or schizophrenia and comparing an AP with another AP or placebo were included. The primary outcome of this analysis was the association of APs with the incidence of cardiovascular death (CVD), myocardial infarction (MI), and stroke. Mantel–Haenszel odds ratios with 95% confidence intervals (MH-OR) were calculated.
**Results:** Of the 3013 studies screened, 92 met the selection criteria (Fig. 1), with 11, 6 and 24 studies reporting data on MI, stroke and CVD, respectively. No significant difference was observed with respect to MI and stroke; a significantly higher cardiovascular mortality was observed for sertindole when compared to risperidone.
**Conclusions:** The paucity of available data does not allow to estimate the impact of APs on cardiovascular morbidity and mortality in the longer term. Long-term metabolic and cardiovascular effects of antipsychotics deserve to be studied more extensively: the request by regulatory authorities of cardiovascular safety data from specifically designed trials would be useful.

### O0061

### 1Hz rTMS to the SMA added to iTBS of the LDLPFC enhances response in highly ruminative refractory depression

J. Davila[1], F. Cruz[1], C. Mejia[1], G. Davila[*,1] and I. Davila[2]
[1]Colombia, and [2]United States of America

**Introduction:** Intermittent theta burst stimulation (iTBS) of the left dorsolateral prefrontal cortex (L-DLPFC) is an effective treatment for patients with treatment-resistant major depressive disorder (TRMDD). Nonetheless, a significant number of patients are nonresponsive to iTBS treatment.
**Objectives:** To compare the effectiveness of iTBS of the L-DLPFC vs. iTBS of the L-DLPFC in addition to low frequency rTMS of the supplementary motor area (SMA) in patients with highly ruminative TRMDD.
**Methods:** Patients, 22-65 years, with TRMDD (n=44) were allocated to Group 1 (n=18, daily sessions of iTBS of the L-DLPFC, 50 Hz, 80% of MT, 20 trains, 8s intertrain interval, 1200 to 1800 pps) or Group 2 (n=26, same iTBS protocol added to rTMS of the SMA, 1 Hz, 100% of MT, 2000 pps), during 4-6 weeks (20-30 sessions). Symptoms were evaluated with the HDRS-17 (score ≥ 18) and the Ruminative Response Scale (score ≥ 50). Clinical Global Impression (CGI) and Global Assessment of Functioning Scale (GAF) were also applied.
**Results:** Group 1: HDRS-17 improved from 24.5 (SD 4.6) to 14.6 (SD 5.7). Group 2: HDRS-17 improved from 25.1 (SD 4.2) to 11.6 (SD 4.4). (p<0.05). Group 1: RRS improved from 60 (SD 6.3) to 47.6 (SD 9.2). Group 2: RRS improved from 63.7 (SD 7.2) to 43.5 (SD 9.3) (p<0.01)
**Conclusions:** The combined protocol of iTBS of the L-DLPFC in addition to low frequency rTMS of the SMA decreased depressive symptoms and rumination in TRMDD patients more effectively than the iTBS of the L-DLPFC only protocol. This confirms highly ruminative TRMDD is a valid clinical subtype.

### O0062

## Early cognitive improvement as a predictor of rTMS efficacy in schizophrenic patients with depression

N. Maslenikov*, E. Tsukarzi and S. Mosolov

Russian Federation

**Introduction:** Depression is highly prevalent and clinically significant syndrome in schizophrenia which associates with poor outcome, decreased quality of life and increased mortality rates. It is known that depressive, negative and cognitive symptoms of schizophrenia are linked with prefrontal cortex dysfunction, thus rTMS of this target might be beneficial.

**Objectives:** to estimate the possible association between early cognitive improvement and response of rTMS in depression.

**Methods:** 63 schizophrenia (ICD-10) patients with prominent and persistent negative symptoms (mean PANSS composite index was -11,03±5,48) and depression (CDSS score ≥ 6) on stable medication

**Table 1. Demographic Profile and Clinical Characteristics**

| Demographics and clinical characteristics | *Total (n=160)* | *Psychotic depression (n=50)* | *Non Psychotic depression (n=110)* | *P value* |
|---|---|---|---|---|
| **Age, years (SD)** | 50.8 (15.7) | 54.4 (15.1) | 49.2 (15.7) | NS |
| **Gender** | | | | |
| % Female | 59.3% (95/160) | 52.0% (26/50) | 62.7% (69/110) | NS |
| **Admission status** | | | | **<0.001** |
| Involuntary | 56.9% (91/160) | 66% (33/50) | 32.7% (36/110) | |
| Voluntary | 43.1 % (69/160) | 34% (17/50) | 67.3% (74/110) | |
| | | | | |
| **Duration of current episode** | | | | NS |
| Acute <12 months | 151 | 47 | 104 | |
| Subacute 13-24 months | 5 | 1 | 4 | |
| Chronic > 24 months | 4 | 2 | 2 | |
| | | | | |
| **Number of previous episodes** | | | | <0.001 |
| 0 | 29 | 17 | 12 | |
| 1-3 | 63 | 21 | 42 | |
| >3 | 68 | 12 | 56 | |
| | | | | |
| **Number of failed antidepressants** | | | | NS |
| 2 or less medications | 66.9% (107/160) | 78% (39/50) | 61.8% (68/110) | |
| 3-4 medications | 26.9% (43/160) | 20% (10/50) | 30.0% (33/110) | |
| 5-6 medications | 5.0% 8/(8/160) | 2% (1/50) | 6.4% (7/110) | |
| 7-10 medications | 1.3% (2/160) | 0 | 1.8% (2/110) | |
| | | | | |
| **Augmentation used** | | | | |
| YES | 51.9% (83/160) | 46% (23/50) | 54.5% (60/110) | NS |
| | | | | |
| **Past ECT treatment** | | | | NS |
| NO | 98 | 35 | 63 | |
| YES with no/minimal response | 3 | 1 | 2 | |
| YES with partial/good response | 59 | 14 | 45 | |
| | | | | |
| **Main reason for ECT** | | | | NS |
| Failure of medication | 58.1% (93/160) | 62.0% (31/50) | 56.4% (62/110) | |
| High suicide risk | 4.4% (7/160) | 6.0% (3/50) | 3.6% (4/110) | |
| Inadequate oral intake | 8.8% (14/160) | 14.0% (7/50) | 6.4% (7/110) | |
| Patient preference | 8.1% (13/160) | 0 | 11.8% (13/110) | |
| Previous good ECT response | 20.6% (33/160) | 18.0% (9/50) | 21.8% (24/110) | |
| | | | | |
| **ECT type** | | | | NS |
| Bi-frontal 1.0ms pulsewidth | 6.9% (11/160) | 12.0% (6/50) | 4.5% (5/110) | |
| Bi-temporal 0.5ms pulsewidth | 11.3% (18/160) | 18.0% (9/50) | 8.2% (9/110 | |
| Right Unilateral 1.0ms pulsewidth | 0.6% (1/160) | 0 | 0.9% (1/110) | |
| Right Unilateral 0.3ms pulsewidth | 81.3% (130/160) | 70.0% (35/50) | 86.4% (95/110) | |
| | | | | |
| **Seizure threshold**[a] | 8.0% | 12.1% (8.5) | 5.9% (3.8) | NS |
| **1st ECT treatment dose**[b] | 31.0% (20.4) | 39.5% (29.4) | 27.2% (13.4) | NS |
| **Propofol dose in mg (SD)** | 58.0 | 48.7 (16.5) | 60.6 (16) | NS |
| **CGI-S scores** | 4.7 (0.7) | 5.1 (0.7) | 4.5 (0.6) | **<0.001** |
| **GAF scores** | 45.1 (7.9) | 40.3 (7.3) | 47.3 (7.1) | NS |
| **MADRS scores** | 32.1 (9.1) | 33.8 (9) | 31.5 (9) | NS |
| **MOCA Total score** | 22.4 (7.3) | 18.3 (8.1) | 23.9 (6.5) | **0.011** |

[a]Seizure threshold was determined at the first ECT session, starting at a dose level of 50mC (or 10% of machine energy) and titrated upwards till a seizure of at least 15 seconds was induced. % machine energy can be converted to mC by multiplying by a factor of 5.04.
[b]After seizure threshold was determined, the 1st ECT treatment was administered at 1.5 times seizure threshold.
NS, not significant

**Table 2. Response and remission rates**

| | Total | Total Missing data[a] | Psychotic depression | Non psychotic depression | p value |
|---|---|---|---|---|---|
| Response rate | 60.0% (52/86) | 46.3% (74/160) | 79.3% (23/29) | 50.9% (29/57) | **0.009** |
| Remission rate | 47.8% (43/90) | 43.8% (70/160) | 71.0% (22/31) | 35.6% (21/59) | **0.001** |

[a]Missing data was excluded from the results were included in the study. Patients received 15-Hz rTMS on the left DLPFC (100% intensity, 15 sessions of 1800 pulses). Patients were assessed weekly with CDSS, PANSS, and with the battery of cognitive tests (“10 words”, Benton’s test, “Coding”, “Switching count”, “Verbal fluency test”). The criterion of response was 50% CDSS score reduction after 3 weeks of treatment.

**Results:** The response rate was 63,5% (40 patients). Mean CDSS score reduction was 53,1% (p<0,000001), mean PANSS negative subscale score reduction was 18,3% (p<0,000001). There was improvement in cognitive tests for executive functions and attention switching without significant changes in verbal and visual memory. Rapid cognitive response (“Coding” score increase ≥10% after 1 week) matches with rTMS response (Chi-square 4,03, p=0,0446).

**Conclusions:** rTMS is effective in treatment of depression in schizophrenia. rTMS reduces secondary negative symptoms and improves some cognitive functions. Rapid cognitive response might be a favorable prognostic sign of rTMS treatment course. Further trials are needed.

**Conflict of interest: No**

**Table 3. Pre vs Post ECT results (CGI, QLES, GAF, EQ5D, VAS and MoCA)**

| | **Total** | | | **Psychotic depression** | | | **Non Psychotic depression** | | |
|---|---|---|---|---|---|---|---|---|---|
| | **Pre ECT (n=160)** | **Post ECT (n=90)** | **P value** | **Pre ECT (n=50)** | **Post ECT (n=31)** | **P value** | **Pre ECT (n=110)** | **Post ECT (n=59)** | **P value** |
| CGI[a] | 4.7 (0.7) | 2.9 (0.8) | **<0.001** | 5.1 (0.7) | 2.9 (0.8) | **<0.001** | 4.5 (0.6) | 3.0 (0.8) | **<0.001** |
| MADRS | 32.1 (9.1) | 14.1 (10.7) | **<0.001** | 33.8 (9.0) | 15.8 (10.9) | **0.034** | 31.5 (9.0) | 10.9 (9.6) | **0.003** |
| GAF[b] | 45.1 (7.9) | 61.0 (9.0) | **<0.001** | 40.3 (7.3) | 60.9 (10.0) | **0.049** | 47.3 (7.1) | 61.1 (8.5) | NS |
| QLES[c] | 32.9 (10.3) | 44.2 (10.1) | **<0.001** | 33.4 (7.6) | 49.1 (8.6) | **<0.001** | 34.3 (7.9) | 42.3 (10.2) | **<0.001** |
| QLES Item 15[d] | 2.8 (1.0) | 3.6 (0.9) | **<0.001** | 2.3 (1.0) | 3.8 (0.9) | **0.001** | 2.9 (1.0) | 3.4 (0.8) | **0.009** |
| QLES Item 16[e] | 2.3 (1.0) | 3.5 (1.0) | **<0.001** | 2.7 (1.0) | 3.7 (0.9) | **0.025** | 2.3 (1.0) | 3.4 (1.0) | **<0.001** |
| **MoCA[f]** | | | | | | | | | |
| Total score | 22.4 (7.3) | 23.1 (6.3) | **0.022** | 18.3 (8.1) | 20.4 (6.3) | **0.038** | 23.9 (6.5) | 24.3 (6.0) | NS |
| Visuospatial / Executive | 3.3 (1.9) | 3.4 (1.8) | NS | 2.3 (2.1) | 2.6 (1.9) | NS | 3.6 (1.8) | 3.7 (1.6) | NS |
| Naming | 2.7 (0.9) | 2.9 (0.6) | **0.040** | 2.5 (1.1) | 2.9 (0.6) | NS | 2.7 (0.8) | 2.8 (0.6) | NS |
| Attention | 4.4 (2.2) | 4.9 (1.7) | **0008** | 3.4 (2.5) | 4.7 (1.8) | **0.024** | 4.8 (1.9) | 5.0 (1.6) | NS |
| Language | 1.8 (1.2) | 2.1 (1.0) | **0.006** | 1.2 (1.3) | 1.9 (1.1) | **0.008** | 2.0 (1.1) | 2.2 (0.9) | NS |
| Abstraction | 1.1 (0.9) | 1.3 (0.9) | **0.049** | 0.9 (0.9) | 1.2 (0.9) | NS | 1.2 (0.9) | 1.3 (0.9) | NS |
| Delayed Recall | 2.8 (2.0) | 2.9 (1.9) | NS | 1.5 (1.8) | 2.0 (1.8) | NS | 3.3 (1.9) | 3.3 (1.9) | NS |
| Orientation | 4.8 (1.8) | 5.2 (1.5) | **0.023** | 4.0 (2.1) | 5.2 (1.3) | **0.021** | 5.1 (1.7) | 5.2 (1.6) | NS |

[a]Clinical Global Impressions Improvement Scale,
[b]Global Assessment of Functioning Scale
[c]Quality of Life Enjoyment and Satisfaction Questionnaire (Items 1 to 14)
[d]Quality of Life Enjoyment and Satisfaction Item 15, “How satisfied have you been with your medication?”
[e]Quality of Life Enjoyment and Satisfaction Item 16, “How would you rate your overall life satisfaction and contentment over the past week?”
[f]Montreal Cognitive Assessment (MoCA, Singapore validated version)]

### O0065

#### Psychological factors of effective rehabilitation after sport trauma: Validation of the illness and treatment self-regulation questionnaire

A. Yavorovskaia, E. Rasskazova* and S. Leonov
Russian Federation

**Introduction:** Psychological factors that are important for rehabilitation after sport trauma (Ivarsson et al. 2017, Weiss, 2003) demand for appropriate assessment. Illness and Treatment Self-Regulation Questionnaire (Kovyazina et al., 2019) was validated for after-stroke patients ongoing rehabilitation and includes 11 items for 4 scales measuring rehabilitation-related helplessness, health anxiety, self-efficacy and confidence.
**Objectives:** The aim was to validate Illness and Treatment Self-Regulation Questionnaire in sportsmen after sport trauma.
**Methods:** 35 sportsmen from different sports in the Rehabilitation center (17-29 years old, 24 males), 61 athletes (15-25 years old, 31 males) and 39 football and hockey players (16-17 years old, males) after sport trauma filled Illness and Treatment Self-Regulation Questionnaire (Kovyazina et al., 2019), Satisfaction with Life Scale (Diener et al., 1985), Scale of Positive and Negative Experience (Diener et al., 2009), COPE with specific instruction about trauma (Carver et al., 1989).
**Results:** Cronbach's alphas varied for different scales and samples .60-.85. In all three samples health anxiety and helplessness in rehabilitation are related to poorer well-being (|r|=.26-.68). Helplessness is related to poorer positive reinterpretation of trauma (r=-.55 - -.34), behavioral disengagement (r=.33-.50), health anxiety – to emotions venting (r=.35-.47), self-efficacy – to active coping with trauma (r=.22-.52), confidence in treatment – to better planning (r=.31-.35).
**Conclusions:** Illness and Treatment Self-Regulation Questionnaire could be used in psychological rehabilitation of sportsmen after trauma for screening assessment of helplessness, self-efficacy, health anxiety and confident in treatment. Research is supported by the Russian Science Foundation, project No. 19-78-10134.
**Disclosure:** Research is supported by the Russian Science Foundation, project No. 19-78-10134

## ORAL COMMUNICATION SESSION 07: WOMEN, GENDER AND MENTAL HEALTH / ETHICS AND PSYCHIATRY / INTELLECTUAL DISABILITY

### O0067

#### Mother infant feeding interaction (MI-FI): Improved eating habits at 1 year

Y. Latzer*, G. Rozen, I. Elad and I. Globus
Israel

**Introduction:** Childhood obesity, poor eating habits, and eating problems are increasing. Parents are often at a loss about how to tackle these problems.
**Objectives:** This study examined whether professional behavioral and nutritional training for first-time mothers can improve feeding interaction at age 12 months.
**Methods:** Participants were 128 mother-infant dyads: 86 in the intervention group and 42 controls. Mother's' age was M=30 years (+2.6), with M=16 (+2.2) years of education. Intervention group received Mother Infant Feeding Interaction (MI-FI) training: four weekly workshops for mothers when infants were 4-6 months, followed by continued internet-based support by pediatric dietitian and social worker until infants reached 12 months. Control group received municipal well-baby clinic's standard mother-infant support. We assessed mothers' tolerance to ambiguity and feeding-related reports. Blinded coders evaluated videotaped home mealtime interactions (age 12 months) using Chatoor Feeding Scale (CFS).
**Results:** Significant inter-group differences emerged in mealtime interactions for four of the five CFS dimensions: dyadic conflict (MI-FI = 4.69 vs. control = 8.38), talk and distraction (3.75 vs. 4.90), struggle for control (2.30 vs. 4.88), and maternal non-contingency (1.61 vs. 2.75). Findings indicated significantly more positive mother-infant mealtime interactions and maternal responses to infant cues in the MI-FI group than in controls.
**Conclusions:** Very early maternal training may support development of more positive mother-infant feeding interactions. This may contribute to preserved internal hunger and satiety, improved eating habits, and prevention of future eating disorders and obesity. Long-term follow-up may optimize training for specific target populations.

### O0071

#### Prescriptions of psychotropic medications among finnish women using hormonal contraception

E. Toffol*, T. Partonen, O. Heikinheimo, A. Latvala, A. But and J. Haukka
Finland

**Introduction:** Approximately 70% of married/cohabiting European women use contraception (www.ContraceptionInfo.eu), and 40% of childbearing aged Finnish women choose a hormonal method. Women with previous or ongoing mental problems use contraception inconsistently. On the other hand, mood symptoms are common and often a cause of discontinuation of hormonal contraception (HC). However, the associations of HC with mental health problems remain unclear.
**Objectives:** Starting from 2017, HC (except for intrauterine devices) users in Finland can be identified from register records. This nation-wide, register-based study aims at examining the associations between prescriptions of HC and of psychotropic medication in Finland in 2017.
**Methods:** Our cohort included all women living in Finland, with at least one redeemed HC prescription in 2017; the reference cohort of non-users was matched by age and municipality of residence. The proportions of HC users and non-users using psychotropic medications in 2017, as in the Prescription Center, were compared via chi-squared test.

**Results:** The cohort of HC users included 294,445 women aged 15–49 years, 25.8% of Finnish female population of that age range. While antipsychotics were more common among non-users (3.9% vs. 3.7%, p=0.001), HC users were more likely to be prescribed with antidepressants (13.2% vs. 12.1%), anxiolytics (5.7% vs. 5.2%), sedatives/hypnotics (6.0% vs. 5.2%) or a combination of them (0.4% vs. 0.3%) (p<0.001). No differences were found in prescription rates of psychostimulants.
**Conclusions:** Prescriptions of Psychotropic medications, except for antipsychotics and psychostimulants, are more common among HC users. The nature of these findings will be further examined in a long-term prospective register-based setting.

### O0072

### Pharmacokinetics of lithium in nursing infants

M.L. Imaz*, M. Torra, D. Soy, L. Garcia-Esteve and R. Martin-Santos
Spain

**Introduction:** Lithium is an effective first-line treatment for bipolar disorder in the perinatal period. Historically women have been instructed to avoid breastfeeding while taking lithium due to the high variability of the transfer into breastmilk and the possible risk of lithium toxicity in the nursing infants.
**Objectives:** The aim of the study was to evaluate the pharmacokinetics of lithium at delivery and during lactation
**Methods:** The Unit of Perinatal Mental Health Clinic-Barcelona, recruited and prospectively followed nine women with bipolar disorder treated with lithium monotherapy during late pregnancy who elected exclusive breastfeeding. Study variables: socio-demographic, psychopharmacologic treatment, neonatal and child outcomes. Lithium plasma concentrations were collected intrapartum [maternal blood (MB), umbilical cord (UC)] and simultaneously in mother-infant pairs during lactation. Lithium plasma concentrations were determined by means of an AVL 9180 electrolyte analyzer based on the ion-selective electrode (ISE) measurement principle. Detection limit was 0.10 mEq/L.
**Results:** Lithium equilibrates across the placenta [mean (SD) UC/MB ratio 1.12 (0.10)]. The infant plasma to-maternal plasma lithium concentration (I/P) ratio decreased by the time from 1.12 (delivery) to 0.28 (delivery + 45.90 days). At seven weeks after birth, the infant plasma lithium concentrations ranged from 0.10-0.20mEq/L. No signs of lithium toxicity or other clinical adverse events were observed in the breastfeed infants.
**Conclusions:** Llithium crosses the placenta completely and the concentrations in infant plasma decreased across time. We did not observe signs of lithium toxicity in nursing infants. Our suggestion is to monitor lithium concentration in mother-infant dyads at delivery, at 2, 10, 30 and 60 days postpartum.

### O0073

### Discriminant validity of the prenatal obsessive-compulsive scale – distinguishing between perinatal OC, depression and anxiety in pregnancy

A.T. Pereira, A. Araújo*, S. Xavier, J. Azevedo, M.J. Soares, M. Marques and A. Macedo
Portugal

**Introduction:** Prenatal obsessive-compulsive (prOC) phenomena lack reliable characterization, as most studies focus on the postpartum. The Perinatal Obsessive-Compulsive Scale (POCS; Lord, 2011) may contribute to improve our understanding and the approach to preOC, namely, in the discrimination between prenatal OC phenomena and anxiety/depression.
**Objectives:** To analyze if women with clinical Obsessive-Compulsive Disorder (scores above the POCS cutoff) differ from women with clinical depression (scores above the Perinatal Depression Screening Scale-24/PDSS cutoff) and clinical anxiety (scores above the Perinatal Anxiety Screening Scale/PASS cutoff), in pregnancy, in OC Severity and Interference as assessed by the POCS.
**Methods:** 345 women (mean age=32.20±5.241) in the second trimester of pregnancy (mean gestation weeks =17.86±4.744) filled in the Portuguese versions of the PDSS, the PASS and the POCS. Women with scores above the cutoff in more than one questionnaire were excluded. Non-parametric tests were applied (p<.008).
**Results:** OC_Severity and OC_Interference significantly differed in women with scores below PDSS/PASS/POCS cutoffs (67.5%; Mean±SD=7.12±5.26 and 3.49±4.05) vs. women with POCS>20 (7.5%; 13.23±4.18 and 7.11±5.14); women with POCS>20 also presented significantly higher scores (only in OC_Severity) than women with PDSS>44 (8.7%; 7.62±6.191) and with PASS>28 (2.0%; 8.40±5.13 and 7.60±7.23). Women with PDSS>44 vs. PASS>28 vs. with scores above PDSS/PASS/POCS cutoffs did not significantly differ in OC_Severity and OC_Interference.
**Conclusions:** These results prove the clinical discriminant validity of the POCS and show that it can be useful in the discrimination between obsessions/covert compulsions and other forms of repetitive negative thinking, also present in women with perinatal anxiety (eg. worry) and depression (eg. rumination).

### O0074

### Empathy and personality traits in general practitioners

D. Mnif*, R. Sellami and D. Jmal
Tunisia

**Introduction:** Different factors influence the empathy of the doctor, especially the personality.
**Objectives:** To study different factors that can influence the physician's empathy and the dimensions of the personality of the doctors to establish the link that can exist between these dimensions and the different dimensions of empathy.

**Methods:** A cross-sectional analytical survey carried out among 120 public health doctors. We collect different personal and professional variables using a pre-established questionnaire. We used the Jefferson Hojat Empathy Scale for the assessment of empathy. The study of the personality was carried out using the "Big Five" scale.
**Results:** The average of the empathy scores among our participants was 96.24. We found that the "PP" dimension score increased with age, was significantly correlated with having psychotherapy training ($p = 0.002$), having training about communication ($p = 0.004$) and was higher among physicians with A dimension of personality ($p = 0.03$). The score of the "CE" dimension was higher among doctors who spend time with their entourage ($p = 0.034$), who practice a leisure activity and who do not have children ($p = 0.002$). The "Spis" dimension was lower in physicians with a high number of hours ($p = 0.022$) and a high number of consultants and it was higher in physicians with an E dimension of personality ($p = 0.038$). For the JSPE total score, it was significantly higher among physicians with training in psychotherapy ($p = 0.014$).
**Conclusions:** We should now find a link between the best method to develop empathy based on personal representation.

### ORAL COMMUNICATION SESSION 08: PREVENTION OF MENTAL DISORDERS / SCHIZOPHRENIA AND OTHER PSYCHOTIC DISORDERS - PART II

#### O0077

#### Clinical and biological signs of the schizophrenia prodrome

M. Omelchenko*, Z. Sarmanova, S. Zozulya and V. Kaleda
Russian Federation

**Introduction:** Early detection of schizophrenia is one of most important questions in psychiatry. Apart from clinical signs, which are often represented by depressive symptoms, dysfunction of the immune system is also found at the early stages of psychosis.
**Objectives:** Detection of clinical and immune markers of schizophrenia prodrome in young adults with the first episode of depression.
**Methods:** 66 young in-patients (average age 19.7 years) with first depressive episode were divided into three groups: 1 - depression with attenuated psychotic symptoms (APS), 2 - depression with attenuated negative symptoms (ANS), 3 - pure youth depression without “schizophrenic signs”. The scales HDRS, SOPS and SANS were applied to assess the severity of depression, APS and ANS respectively. The activity of inflammatory markers - leukocyte elastase (LE) and α1-proteinase inhibitor (α1-PI) - was determined in serum.
**Results:** An increase in the activity of inflammatory LE compared with the control group was found in all patients. An increase in the activity of α1-PI in groups 1 and 3 ($p<0.0001$; $p=0.0044$) was detected. Clinical and biological correlations were found: between activity LE, α1-PI and the total score on the HDRS ($r=0.55$; $p=0.023$ and $r=-0,48$; $p=0,032$) in the 1st group; between the activity LE and the positive SOPS subscale ($r=0.52$; $p=0.027$) and the negative SOPS subscale ($r=-0,46$; $p=0,05$) in the 2nd group; between LE activity and the score on the HDRS ($r=0,47$; $p=0,038$) and SANS ($r = 0,63$; $p=0.049$) in the 3rd group.
**Conclusions:** The study discovered specific links between the structure and severity of youth depression and inflammatory markers.

#### O0078

#### Experience of cyberbullying predicts hostility and interpersonal anxiety in youth and adults

G. Soldatova, D. Vinitskiy*, E. Rasskazova and S. Chigarkova
Russian Federation

**Introduction:** Internet creates a new sphere of risks that is especially important for adolescents and youth (Livingstone et al., 2012). Although cyberbullying is typically a part of offline bullying, it is still unclear whether it is specifically related to anxiety, depression or aggression or is reducible to other variants of bullying.
**Objectives:** The aim was to reveal the relationship between experience of cyberbullying and psychopathological symptoms in two generations: youth comparing to adults.
**Methods:** 736 youth (18-30 years) and 1105 adults (30-65 years) from 8 Federal regions in Russia appraised their past experiences of bullying at school, University, work and the Internet, filled Anxiety and Interpersonal Sensitivity scales from SCL-90R (Derogatis, 1994), Aggression Questionnaire (Buss, Perry, 1992).
**Results:** After adjusting for experience of bullying at school, University and work, cyberbullying predicted hostility ($\beta=.12-.15$, $\Delta R^2=.9-1.5\%$, $p<.05$) and interpersonal sensitivity ($\beta=.09-.12$, $\Delta R^2=.6-1.0\%$, $p<.05$). In adults experience of cyberbullying also predicted anger ($\beta=.12$, $\Delta R^2=1.0\%$, $p<.05$) while in youth – anxiety ($\beta=.11$, $\Delta R^2=.8\%$, $p<.05$). There was no effect of cyberbullying on physical aggression.
**Conclusions:** Data suggests that cyberbullying is weak but significant predictor of interpersonal sensitivity and hostility and should be considered together with other types of offline bullying. Study is supported by the Russian Science Foundation, project 18-18-00365.
**Disclosure:** Study is supported by the Russian Science Foundation, project 18-18-00365

#### O0082

#### Mthfr polymorphisms as possible markers of treatment response in first-episode psychosis

D. Bosnjak Kuharic*, P. Makaric, T. Sabo, D. Ostojic, A. Silic, A. Savic, P. Brečić, N. Prpic and M. Rojnic Kuzman
Croatia

**Introduction:** Different studies have tried to identify possible genetic biomarkers of treatment outcomes in schizophrenia. Methylenetetrahydrofolate reductase (MTHFR) gene was previously associated with schizophrenia, metabolic risk and side-effects as well as with neurocognitive functions and treatment response in schizophrenia.

**Objectives:** The objective of this study was to investigate the association of MHTFR 1298 polymorphisms with treatment response measured by changes in facial emotional recognition (FER) and psychopathology in patients with first-episode psychosis (FEP).
**Methods:** We conducted a prospective study including 159 patients with FEP recruited from two Croatian psychiatric hospitals. We performed assessment two weeks upon admittance to hospital and after 18 months of follow-up. We assessed psychopathology with the Positive and Negative Syndrome Scale (PANSS) and FER with Penn Emotional Recognition Test. Genotyping of blood samples was preformed according to usual practice. Statistical analysis included ANOVA.
**Results:** Our results did not show statistically significant association of MHTFR genotypes and correct recognition of presented emotions. However, we found significant associations with initial general (F=3.36, p=0.04) and overall PANSS scores (F=3.14, p=0.05) as well as with changes of negative (F=-1.562, p=0.012), general (F=-2.686, p=0.005) and overall PANSS scores (F=-5.417, p=0.006). In all cases, the carriers of the CC alleles had better scores.
**Conclusions:** While we did not find association of specific MHTFR genotypes and FER, they showed strong association with both initial and changes of psychopathology scores after 18 months of follow-up which opens the possibility of their role as potential marker for treatment outcomes.

### O0083

#### Efficacy of cariprazine in the treatment of acute and primary negative symptoms of schizophrenia: Posthoc analyses versus risperidone

B. Sebe[1]*, Á. Barabássy[1], Á. Balogh[1], K. Acsai[1], I. Laszlovszky[1], B. Szatmári[1], J. Harsányi[1], M. Patel[2], W. Earley[2] and G. Németh[1]
[1]Hungary and [2]United States of America

**Introduction:** Although four atypical antipsychotics, including risperidone, showed better efficacy than typical antipsychotics in a large meta-analysis, specific studies failed to verify their effect on primary negative symptoms. Cariprazine, a D3/D2 partial agonist has proven to be effective in the treatment of acute and predominantly negative symptoms (PNS) of schizophrenia.
**Objectives:** The objective of this analysis is to evaluate the effects of cariprazine versus risperidone in the treatment of acute and PNS schizophrenia.
**Methods:** Analyses are based on one short-term, placebo and risperidone controlled study (NCT00694707) investigating the efficacy of cariprazine on acute symptoms (intention to treat population) and negative symptoms (subgroup with PNS); and on a long-term, risperidone controlled study (EudraCT, number 2012-005485-36) in PNS patients. Results are presented in equivalent doses of cariprazine (4.5mg/d) and risperidone (4mg/d). Patients prior stabilized on risperidone were excluded from risperidone groups.
**Results:** In acute patients significant differences were observed for both cariprazine (LS Mean CAR:-23.7, p<0.0001) and risperidone (LS Mean:-26.3; p<0.0001) versus placebo (LS Mean:-13.1) on the PANSS total score. Cariprazine and risperidone were equally effective (LSMD cariprazine vs risperidone:2.6, p=0.4071). In the PNS subgroup, PANSS-FSNS changes were significant for cariprazine (LS Mean:-8.3; p=0.0379), but not for risperidone (LS Mean:-7.1; p=0.3607) versus PBO (LS Mean:-5.6). In the long term PNS study cariprazine outperformed risperidone (LSMD:-1.46; p=0.0022) in controlling negative symptoms (PANSS-FSNS changes).
**Conclusions:** While cariprazine and risperidone equally controlled overall symptoms of acute patients, cariprazine resulted better in the treatment of predominant negative symptoms of schizophrenia.
**Disclosure:** I am employee of Richter Gedeon Plc.

### O0085

#### Verbal fluency of patients with paranoid schizophrenia, first grade schizophrenia relatives and healthy controls – comparative study

M. Stoimenova-Popova*, I. Veleva, P. Chumpalova, L. Tumbev, R. Yakimova, E. Dimitrova, E. Krasteva and M. Valkova
Bulgaria

**Introduction:** Schizophrenia is considered to be neurobiological disease with basic neurocognitive deficit. Cognitive impairments are a key symptom of the disease and are associated with the hypothesis of impaired neurodevelopment due to schizophrenia. The impairments in the verbal fluency (VF) are referred to the earliest indicators for cognitive deficit.
**Objectives:** The aim of our study was to compare VF performances between patients with paranoid schizophrenia (PS), first grade relatives (FGR) and healthy controls (HC).
**Methods:** The research was carried out on 108 patients with PS, 58 FGR and 60 HC via Isaack's Set Test (IST) for semantic and test for literal fluency (LF) type K-A-M-E.
**Results:** The average results in the patients for IST is 28.59 words. The FGR generate an average of 32.91 words, and the HC - 34.38 words (p>0.005). In relation to the LF the data show that PS have generated on average 18.05 words, the FGR generate on average 21.66 words, and the HC on average 23.25 words (p= 0.0021). Patients with PS have lower results on IST and LVF than FGR and HC, they show also qualitative changes, while the FGR and the HC distinguish only on LF. Ageing, formal education, duration and severity of the disease, negative symptoms and symptoms of disorganization are associated with tests performances.
**Conclusions:** Severity of PS and particularly positive and negative syndromes, is associated with VF deficit. FGR also show low LF. These deficits are probably due to primary executive dysfunctions associated with neurodevelopment changes.

### O0086

#### Cognitive function in patients with schizophrenia and affective disorder: Effects of combining pharmacotherapy with cognitive remediation

A. Erfurth*, E. Maihofer and G. Sachs
Austria

**Introduction:** Cognition is essential for self-reliant functioning and health-related quality of life. Psychiatric patients suffer from cognitive impairment as a core feature of their illness. Non-pharmacological interventions such as cognitive remediation have been proven effective in ameliorating cognitive performance.
**Objectives:** We aim to investigate whether psychiatric patients with different diagnoses and admitted to a psychiatric department in a defined epidemiological catchment area differ from each other due to their premorbid intelligence and cognitive functioning. Furthermore, the effects of a combination therapy including psychotropic drugs and cognitive remediation were assessed to detect possible improvement.
**Methods:** Patients received combination treatment with psychotropic drugs and cognitive remediation using COGPACK. Cognitive assessment was performed by means of the Screen for Cognitive Impairment in Psychiatry (SCIP). Premorbid intelligence was recorded using Mehrfachwahl-Wortschatz-Intelligenztest (MWT-B). Table 1: Characteristics of patients

| total no. of patients | n= 119 |
|---|---|
| age median (range) | 36 yrs (18-66) |
| female : male | 69 : 50 |
| years of education | 14.4 SD = 3.6 |
| premorbid intelligence | MWT-B mean = 27.36<br>SD = 5.42 |
| diagnoses: | |
| schizophrenia (SCH) | n = 47 |
| bipolar disorder (BD) | n = 31 |
| depression (MDD) | n = 41 |

**Results:** Schizophrenic patients showed the highest degree of cognitive dysfunction whereas affective disorders showed an intermediate degree of impairment. At discharge all patient groups showed significantly enhanced global cognition (p < .001). Patients with depression almost performed on a level of healthy subjects, followed by bipolar patients. In all five SCIP domains patients with schizophrenia showed significant cognitive impairment compared to norm population. We were able to show improvement in cognitive function after a combination treatment in patients with schizophrenia ($p < 0.001$), major depression ($p < 0.001$) and bipolar disorder ($p < 0.001$).
**Conclusions:** To our knowledge this is the first study to show cognitive improvement after combination treatment in non-selected, non-research facility patients.

### ORAL COMMUNICATION SESSION 10: DEPRESSIVE DISORDERS - PART I

#### O0088

#### Baseline EEG beta spectral power predicts the response to treatment in depressive patients

A. Iznak*, E. Iznak, L. Abramova and G. Kobel'Kov
Russian Federation

**Introduction:** One of the ways to depression treatment optimization is early individual prediction of therapeutic response based on pre-treatment EEG parameters.
**Objectives:** The study aimed to reveal pre-treatment EEG parameters informative for individual prediction of therapeutic response in depressive patients.
**Methods:** 70 depressive in-patients (27 males, 43 females, aged 20-50, mean age 36.5 ± 2.6) were enrolled in the study. Resting EEGs were registered before the treatment course, and absolute EEG spectral power values were measured. Quantitative clinical assessments (by HDRS-17, MADRS and CGI-S scales) were obtained after treatment course. Pre-treatment EEG data were matched with post-treatment clinical rating scales scores of the same patients using correlation and multiple regression analyses. Efficacy of mathematical models obtained was evaluated on test sample of 30 depressive patients.
**Results:** Multiple regression equations obtained contained not more than 3 to 4 EEG spectral power parameters (from initial 80). Despite the analysis of wide EEG frequency range (2-30 Hz) only values of high-frequency EEG sub-bands (beta1 — 13-20 Hz and beta2 — 20-30 Hz) reflected cortical hyperactivation were included. The models explained 75% of post-treatment variance of HDRS-17 depression cluster scores, 45% of MADRS scores, 35% of CGI-S scores, and 33% of total sum of HDRS-17 scores. Deviations of predicted vs. real post-treatment HDRS scores varied from 10% to 37% (mean 24%).
**Conclusions:** Results confirm high value of EEG in prediction of therapeutic response in depressive patients and important role of cortical-subcortical interactions in depression pathogenesis. The study funded by RFBR Grant No.18-01-00029a
**Disclosure:** The study funded by RFBR Grant No.18-01-00029a

#### O0089

#### SAGE-217 in major depressive disorder: A phase 3, multicenter, double-blind, randomized, placebo-controlled trial

A. Clayton*, R. Lasser, I. Nandy, A. Sankoh, D. Campbell, B. Werneburg, C. Silber, J. Jonas, S. Kanes and H. Gunduz-Bruce
United States of America

**Introduction:** An estimated 300 million people worldwide are affected with major depressive disorder (MDD). Dysregulation of γ-aminobutyric acid (GABA) signaling is associated with MDD. SAGE-217 is an investigational, oral, neuroactive steroid and $GABA_A$ receptor positive allosteric modulator that has demonstrated reductions in depressive symptoms in a Phase 2 double-blind, randomized, placebo-controlled study in MDD.
**Objectives:** The objective of this Phase 3, double-blind, randomized, placebo-controlled study was to evaluate the efficacy and safety of SAGE-217 in the treatment of adult subjects with MDD.
**Methods:** Subjects with MDD were randomized 1:1:1 to SAGE-217 30 mg, 20 mg, or placebo capsules. Subjects were treated for 14 evenings, then followed frequently for 4-weeks and intervals through 182-days. The primary endpoint was change from baseline in Hamilton Depression Rating Scale (HAM-D) total score at Day

15 compared with placebo. Safety and tolerability were assessed by standard clinical assessments.

**Results:** The initial 4-week follow-up of this Phase 3 study will be completed at the end of 2019. Data will be reported on HAM-D time points Days 3-42 (including Day 15), HAM-D response (≥50% reduction) and remission (≤7), Montgomery–Åsberg Depression Rating Scale (MADRS) scores, and Hamilton Anxiety Rating Scale (HAM-A) scores. Safety and tolerability data will also be reported.

**Conclusions:** This Phase 3 study aims to confirm and extend the previous results in a larger study population utilizing two dose groups versus placebo. These results have the potential to be an important milestone in the development of SAGE-217 in the treatment of individuals with MDD.

**Disclosure:** CMS is an employee of Sage Therapeutics, Inc. and have stock/stock options.

### O0091

### The safety, efficacy, and tolerability of a microbial therapeutic in people with major depression and/or generalized anxiety disorder: Preliminary findings

A. Chinna Meyyappan* and R. Milev

Canada

**Introduction:** The bidirectional biochemical signalling between the gut microbiota and the brain, known as the gut-brain axis, is being heavily explored in current neuropsychiatric research. Analyses of the human gut microbiota have shown considerable individual variability in bacterial content which is hypothesized to influence mood and anxiety symptoms through gut-brain axis communication. Research examining these effects suggests that microbiota transplantation may help improve depression and anxiety symptoms and severity by recolonizing the gastrointestinal tract with healthy bacteria.

**Objectives:** The primary objective is to assess subjective changes in mood and anxiety symptoms throughout the study. The secondary objectives are to assess changes in metabolic functioning and level of repopulation of healthy gut bacteria, safety and tolerability of therapeutic, and effects of early stress on biomarkers of depression/anxiety and response to treatment.

**Methods:** Seven adults diagnosed with MDD/GAD were recruited from Kingston, ON, Canada. Participants consumed daily an encapsulated therapeutic, containing 40 strains of bacteria purified and lab-grown from a single donor, for 8 weeks. Participants completed assessments measuring mood/anxiety/GI symptoms for a 10-week period. Blood/fecal samples were assessed for biomarker changes and level of engraftment from feces.

**Results:** Six of seven individuals responded to treatment (50% improvement in MADRS/GAD-7 scores since baseline). Over the 10-week course, MET-2 significantly decreased MADRS and -GAD-7 scores, $F(1,6) = 14.19$, $p = 0.009$ and $F(1,6) = 18.07$, $p = 0.005$, respectively. This improvement may be mediated by the recolonization of the gastrointestinal tract with healthy bacteria.

**Conclusions:** These preliminary findings may be the first to provide evidence for the role of microbial therapy in treating depression and anxiety.

**Conflict of interest:** No

### O0092

### Depression and non-adherence to medications targeting treatable cardiovascular risk factors in the constances cohort

N. Hamieh*, S. Kab, M. Zins, M. Goldberg, M. Melchior and C. Lemogne

France

**Introduction:** Depression is associated with increased risk of cardiovascular disease but the mechanisms remain mostly unknown.

**Objectives:** To study the association between depression and non-adherence to medications targeting type 2 diabetes, hypertension and dyslipidemia (i.e. treatable cardiovascular risk factors) in the Constances population-based French cohort.

**Methods:** We used Constances data linked to the French administrative health care database (SNDS) to study the longitudinal association between depression (assessed at inclusion with the Center for Epidemiological Studies Depression scale) and non-adherence to medications treating diabetes, hypertension and dyslipidemia over two subsequent periods of 18 months. Binary logistic regression models were adjusted for socio-demographics, body mass index, physical activity, prescribed and followed diet, and normal/abnormal levels of blood pressure, glycaemia, cholesterol and triglycerides at inclusion.

**Results:** Among 4,325 individuals with hypertension, 691 with diabetes and 3,329 with dyslipidemia, 535, 50 and 904 were non-adherent over the first 18 months, and 638, 65 and 1,207 between 19-36 months. Depression was neither associated with non-adherence to medications for hypertension and dyslipidemia over the first 18 months, nor afterwards. However, depression was associated with non-adherence to anti-diabetic medications (odds ratio [95% confidence interval]: 2.32 [1.19-4.52]) over the first 18 months only. Depression was only associated with uncontrolled dyslipidemia level (1.24 [1.02-1.52]), although a similar trend was observed for glycaemia level (1.45 [0.96-2.19]).

**Conclusions:** In a population-based cohort, depression was only associated with non-adherence to anti-diabetic medications in the short run, thus urging clinicians to search for and treat depression in individuals with diabetes.

**Conflict of interest:** No

### O0094

### Signs of early monocyte aging and mevalonate kinase abnormality underlying the full-blown overexpression of monocyte inflammatory genes in major depressive disorder patients after childhood adversity

M. Simon[1]*, C. Schiweck[2], A. Wijkhuijs[3], S. Claes[2], V. Arolt[1], N. Müller[1] and H. Drexhage[3]

[1]Germany; [2]Belgium and [3]Netherlands

**Introduction:** We extensively reported on a higher expression of inflammatory gene clusters in monocytes of Major Depressive Disorder (MDD) patients, particularly those with a history of childhood adversity (CA). In our studies there were also signs that not only inflammatory, but also apoptosis/growth and cholesterol metabolism genes were abnormally expressed.

**Objectives:** To study the expression of growth/apoptosis and cholesterol metabolism genes in circulating monocytes of MDD patients collected at three EU-MOODINFLAME sites.
**Methods:** Adult patients with DSM-IV diagnosed MDD with (N=77) or without (N=58) CA (measured by Childhood Trauma Questionnaire) and healthy controls (HC) with (N=37) and without CA (N=81; reference group) were recruited. Monocyte gene expressions were determined using qPCR.
**Results:** 1. MDD monocytes showed an abnormal expression of two inter-correlating clusters of apoptosis/growth and cholesterol metabolism genes (irrespective of CA). Key genes were BAX, BCL10, EGR1, EGR2, TNF, NR1H3, ABCA1, ABCG1. 2. Overexpression of the inflammatory genes was confirmed and particularly evident in CA patients. Key genes were IL1B, IL6, CCL2, MAPK6, MAFF. The overexpression was accompanied by reduced MVK expression, an entrance enzyme for cholesterol synthesis and defective in familial fever syndromes. 3. Monocytes of healthy individuals with CA also showed overexpression of apoptosis/inflammatory genes though much weaker than MDD cases.
**Conclusions:** Data indicate that enhanced mitochondrial apoptosis (early aging) with low-grade inflammation is a general hallmark of MDD monocytes. Reduction of MVK expression in MDD-CA monocytes might shift mitochondrial apoptosis to inflammatory pyroptosis. CA in healthy individuals does not produce the full-blown inflammatory signature seen in MDD monocytes.

### O0097

### Enhancing the detection of postpartum depression from electronic health records using machine learning algorithms

G. Amit[1]*, I. Girshovitz[1], P. Akiva[1], V. Bar[1], Y. Zhang[2], A. Hermann[2], R. Joly[2], M. Turchioe[2] and J. Pathak[2]
[1]Israel and [2]United States of America

**Introduction:** Postpartum depression (PPD) is a common condition, affecting 10-15% of mothers. PPD screening using the Edinburgh Postnatal Depression Scale (EPDS) has variable reported accuracy. Information from Electronic health records (EHR) may complement EPDS to provide higher detection rate.
**Objectives:** To develop and evaluate a machine learning model for identifying PPD using EHR data.
**Methods:** We analyzed primary care EHR records of 259,096 live births in the UK from 2000 to 2017. PPD was defined as having either a depression diagnosis, antidepressant prescriptions or psychotherapy referrals within 12 months after childbirth. The validation set included 5,823 women with recorded EPDS scores. The remaining population was split to training(70%) and testing(30%) sets. We built a prediction model using EHR information prior and during pregnancy, partially based on variables described in our complementary study (EPA2020 abstract by Zhang et.al.). We evaluated the performance of the EHR-model, compared to EPDS-alone and a combination of EHR+EPDS scores.
**Results:** Table 1 details the main characteristics of the cohort. The strongest contributing variables were antidepressants, diagnosis or symptoms of depression/anxiety, and age. The combined model outperformed EPDS alone (area under the curve(AUC) 0.87 vs. 0.80) and EHR alone (AUC=0.77) (Table 2,Fig.1).

**Table 1:** Cohort characteristics

| | Training-set | Testing-set | Validation-set |
|---|---|---|---|
| N | 177,291 | 75,982 | 5,823 |
| Age(yrs) | 29.92±5.8 | 29.92±5.8 | 30.13±5.7 |
| BMI | 25.44±5.0 | 25.44±5.0 | 25.42±4.9 |
| PPD(%) | 25,671(14.5) | 11,081(14.6) | 1249(21.4) |

**Table 2:** Performance of PPD detection

| Method | AUC[CI-95%] | Sensitivity @specificity0.8 | Specificity @sensitivity0.8 |
|---|---|---|---|
| EHR | 0.765[0.750-0.780] | 0.59[0.557-0.617] | 0.57[0.531-0.603] |
| EPDS | 0.801[0.786-0.818] | 0.72[0.690-0.744] | 0.65[0.553-0.661] |
| EHR+EPDS | 0.867[0.854-0.878] | 0.79[0.767-0.817] | 0.79[0.757-0.819] |

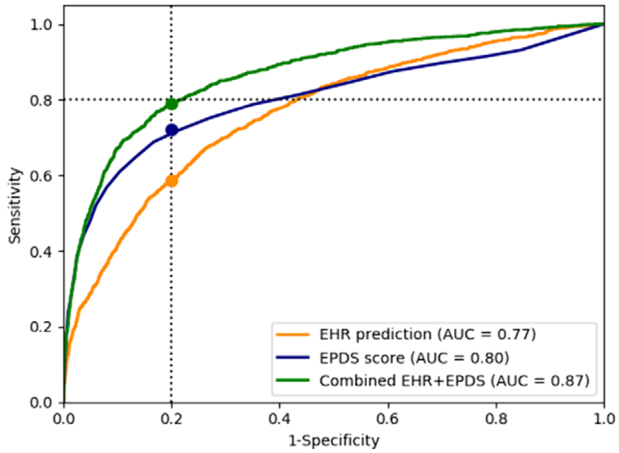

**Conclusions:** EHR-based predictive algorithms can potentially complement and enhance the accuracy of existing PPD screening tools.

## ORAL COMMUNICATION SESSION 11: COMORBIDITY / DUAL PATHOLOGIES / ADDICTIVE DISORDERS

### O0099

### Mental disorders with damage to the diencephalic region

Y. Sidneva*, L. Astaf'Eva, O. Zaitsev, P. Kalinin, M. Kutin, B. Kadashev, I. Voronina, A. Shkarubo, D. Fomichev, O. Sharipov and D. Andreev
Russian Federation

**Introduction:** Craniopharyngioma is a benign brain tumor (up to 3% in adults).
**Objectives:** to study the structure and dynamics of mental disorders in patients with diencephalic lesions on a model of benign tumor of craniopharyngiomas
**Methods:** 120 patients (18-65 years) with craniopharyngiomas: endosuprasellar (n=26), suprasellar (with initial growth in the pituitary stalk, with no penetration into the third ventricle) (n=45), extra-intraventricular (n=35) and intraventricular (n=14). Intraoperatively were used: transcranial approach-78%, transsphenoidal-17%, Ommaya-5%. Methods: psychopathological; data from neurological, endocrinological, neuroimaging.
**Results:** Mental disorders were revealed in 84% before operation in varying degrees of severity. Endo-suprasellar craniopharyngiomas were characterized by paroxysmal (48%), asthenic (48%) disturbances; suprasellar – behavioral, emotional and personality (72%, of them abulia/apathy 7%), memory impairment (56%, of them Korsakoff's syndrome 2%), paroxysmal (40%); extra-intraventricular - memory impairment (72%, of them Korsakoff's syndrome 36%), behavioral, emotional and personality (68%, of them abulia/apathy 21%), paroxysmal (50%); intraventricular – behavioral, emotional and personality (83%, of them abulia/apathy 8%), memory impairment (83%, of them Korsakoff's syndrome 25%), paroxysmal (50%). Frequency of mental disorders decreased by 1,5-2 times after removal of endo-suprasellar tumors; of abulia/apathy (20%) and Korsakoff's syndrome (15%) increased after removal of suprasellar; after removal of extra-intraventricular-Korsakoff's syndrome in 50%, abulia/apathy 30%. 78% of patients were discharged with mental disorders: 23% showed preoperative disorders, 12% less expressed, 43% aggravated (Korsakoff's syndrome 12%, abulia/apathy 8%, combination 3%).
**Conclusions:** Mental disorders were detected in 84% of patients - behavioral, emotional and personality disorders, memory impairment. The increase in psychopathology after removal of craniopharyngiomas is detected in 43% of patients.

### O0105

### Problematic gambling, gaming and internet behavior in heterosexual and sexual minority women

N. Broman[1]*, A. Håkansson[1], F. Prever[2], E. Di Giacomo[2], S. Jimenez-Murcia[3] and A. Szczegielniak[4]
[1]Sweden; [2]Italy; [3]Spain and [4]Poland

**Introduction:** Women with a bi- and homosexual orientation have been associated with having elevated risks for developing substanse use problems compared to heterosexual women and to a greater extent than among sexual minority men (Burgard et al, 2005, Parsons et al, 2006). It raises the question wether there is a greater risk for developing behavioural addictions of gambling and gaming activities among sexual minority women. Two studies have indicated a higher prevalence of gambling problems among sexual minorities (Grantz & Potenza, 2006, Richard et al, 2018). A few publications have shown a higher prevalence of gaming problems among sexual minority populations, including a pilot project to this study in a Swedish setting (Broman & Håkansson, 2018).
**Objectives:** To investigate whether the prevalence of problematic gambling, gaming, internet behaviour differ depending on sexual orientation in a female European study sample.
**Methods:** A web survey was performed in seven European countries; Sweden, Denmark, England, Spain, Italy, Poland, Switzerland. All data was self-reported. Chi-square tests and binary regression analysis were applied.
**Results:** 10 969 complete answers were collected, among whom 771 defined as homosexual, bisexual or other. In descpritive analyses, problematic gambling, gaming and internet behaviour was significantly overrespresented in sexual minority women. Applying regression analysis, sexual minority status remained significant for having a problematic gambling (p=0.036) and gaming problem (p=003).
**Conclusions:** The result in this study is suggesting that behavioural addiction is overrepresented among sexual minority women. In accordance with ealier research, it highlights that sexual minority women need a special focus within clinical addiction settings.
**Disclosure:** No financial support was received specifically for this study. AH holds at position as professor at Lund University, and AH and NB have overall research support from the collaboration between Lund University and the Swedish gambling operator monopoly, Sv

### O0107

### Betting on yourself is never a gamble: Cannabis users fail to take advantage of favourable opportunities in a novel gambling paradigm

S. Perkins*, T.V. Lim and K. Ersche
United Kingdom

**Introduction:** Cannabis remains the most widely used illicit drug in Europe. There is a lack of consensus on the impact of cannabis use on cognitive function; disadvantageous decision-making has frequently been reported in cannabis use disorder, but the early signs are unclear.
**Objectives:** We sought to investigate financial decision-making in cannabis users using a novel paradigm that is not dependent on learning or working memory. We hypothesised disadvantageous choices; either inappropriate risk-taking or selection of unfavourable options.
**Methods:** We recruited 45 healthy young-adults from the general community: Twenty-eight reported harmful cannabis use (CUDIT score >13), 17 did not consume cannabis at all. They performed a roulette task in which bets were placed on anticipated outcomes. Reward and loss trials were administered separately. Statistical tests were conducted using SPSS v25 and were reported as significant if $p<0.05$.
**Results:** The groups were well matched on age and socioeconomic status, but differed on gender and level of education which were controlled for in the analysis. During the task, cannabis users earned as much as controls ($F_{1,40}=0.5$, $p=0.479$) and did not demonstrate any risk-taking behaviour ($F_{1,40}=0.1$, $p=0.780$). However, they placed lower bets on favourable options when reward was at stake ($F_{4,160}=4.9$, $p=0.003$).
**Conclusions:** These preliminary results elucidate a deficit amongst cannabis users in making use of favourable opportunities. This early feature of cognitive dysfunction may likely contribute to the disadvantageous pattern of decision-making apparent in cannabis use disorder. Our findings might represent an opportunity for early intervention, however further research is required to explain the underlying process.

**Disclosure:** Acknowledgments: The authors thank all the participants who agreed to participate in our study, which was partly funded by the Cambridge Biomedical Research Centre and the University of Cambridge School of Clinical Medicine. The authors declare no conflic

## ORAL COMMUNICATION SESSION 09: CONSULTATION LIAISON PSYCHIATRY AND PSYCHOSOMATICS / EPIDEMIOLOGY AND SOCIAL PSYCHIATRY / CHILD AND ADOLESCENT PSYCHIATRY - PART II

### O0110

### Chronic physical illness, multimorbidity and their effects on psychiatric treatment outcomes

I. Filipčić* and I. Šimunović Filipčić

Croatia

**Introduction:** People with severe mental illnesses (SMI) and common mental disorders (CMD) have higher premature mortality than the general population (GEP) largely due to common, preventable chronic physical illness (CPI). In fact, the mortality gap between people with and without SMI or CMD is growing. However, the effect on psychiatric treatment outcomes are not clear

**Objectives:** To outline and discuss differences in the prevalence and patterns of CPI, multimorbidity (≥2 CPI) and their effects on psychiatric treatment outcomes.

**Methods:** Presentation is based on retrospective cohort study conducted on psychiatric patients (PP) (n=1060), and the cross-sectional European Health Interview Survey conducted on Croatian GEP (n=837).

**Results:** PP had 27% ($CI_{95\%}$ 24%-30%; p<0.001) higher age-standardized relative risk for CPI and 31% ($CI_{95\%}$ 28%-34%; p<0.001) higher risk for multimorbidity than the GEP. The younger the patient, the larger was the difference to the GEP counterpart. The most vulnerable group were young women with schizophrenia. The specific multimorbidities' profiles and interconnectedness of different CPIs was very similar between PP and the GEP. Multimorbidity was significantly associated with higher rehospitalization rate in patients with schizophrenia and major depression disorder, and PP lower quality of life.

**Conclusions:** The psychiatric population is disproportionately burdened with an increased risk of CPI and multimorbidity which affects the psychiatric treatment outcomes and patients´ quality of life, and lead to premature mortality. The medical and lifestyle interventions should optimally be integrated into the psychiatric setting and brought to the forefront of psychiatric research.

**Conflict of interest:** No

### O0116

### Behavioral and emotional problems in a large group of italian adolescents

G. Lisi*, R. Rossi, M. Ribolsi, G. Di Lorenzo, C. Parisi, M. Siracusano, L. Morciano, A. Alberto De Stefano, A. Pesaresi, C. Niolu, L. Palombi and A. Siracusano

Italy

**Introduction:** One in six adolescents aged suffers from mental health problems. Despite the presence of general information on Italian adolescents' mental health, provided by periodic epidemiological studies, researches conducted with standardized assessment tools are scarce in the scientific literature.

**Objectives:** To evaluate the prevalence of self-reported behavioral and emotional problems in a group of Italian adolescents and to examine their relation to important socio-demographical variables, in order to provide relevant information about of adolescence mental health in our country.

**Methods:** A population-based survey was conducted on high school students from urban areas of Rome and Latina. Socio-demographic variables, including age, gender, type of school, socio-economic status, urbanicity, as well as Youth Self-Report (YSR) evaluating 3 broadband scales (Internalizing Disorders; Externalizing Disorders; Total Problem scale) were collected. Univariable and Multivariable Logistic regression were used to assess the association between socio-demographic variables and YSR.

**Results:** Our final sample consisted of 1400 adolescents (38.61% male, mean age 16 yrs, SD 1.42). Prevalence of Internalizing Problems, Externalizing Problems and Total Problems was 29.55%, 18.34% and 24.13%. Internalizing Symptoms were not explained by sociodemographic variables, while Externalizing Symptoms were explained by Male Gender (OR=1.53, [1.14,2.06]), older age (OR=2.06, [1.52,2.79]) and attending a Professional Institute (OR=2.15, [1.53,3.02] Adjusted $R^2$=4.32%). Total Problems were explained by School Type (Professional Institutes and Art and Humanities vs. Grammar and Science School; OR 1.93 [1.40,2.67] and 1.64 [1.08,2.47], adjusted $R^2$=1.94).

**Conclusions:** The study estimates the prevalence of self-reported behavioral and emotional problems in a large sample of Italian adolescents, highlighting the role of socio-demographic risk factors for externalizing behaviors.

**Conflict of interest:** No

### O0119

### In-utero patterns of antidepressant use and cognitive development at 3 years of age: Results from the mothertobaby antidepressants study

A. Berard*, V. Tchuente, O. Sheehy and J. Zhao

Canada

**Introduction:** In-utero exposure to antidepressants (AD) have been associated with autism and ADHD. However, no one studied the effect of trajectories of AD use during pregnancy on the overall cognitive functions of children over time.

**Objectives:** To quantify the risk of delay in cognitive function associated with trajectory of AD use during pregnancy.

**Methods:** To be included in the MotherToBaby Antidepressant Study, pregnant women had to 1) call a North American participating

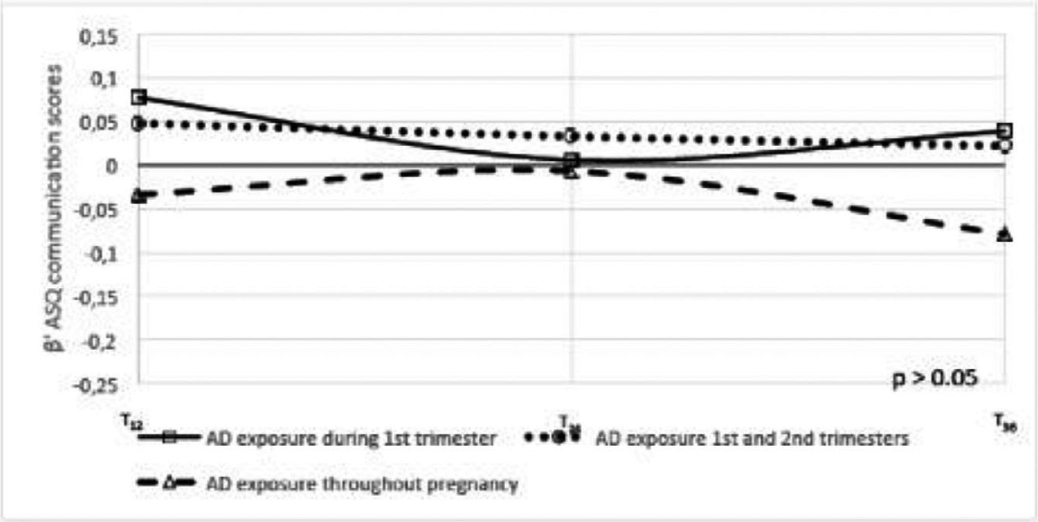

**Fig.3a** Standardized and adjusted association between in-utero antidepressant exposure patterns and **COMMUNICATION scores** assessed with the Ages and Stages Questionnaire (ASQ) compared to non-exposed group.

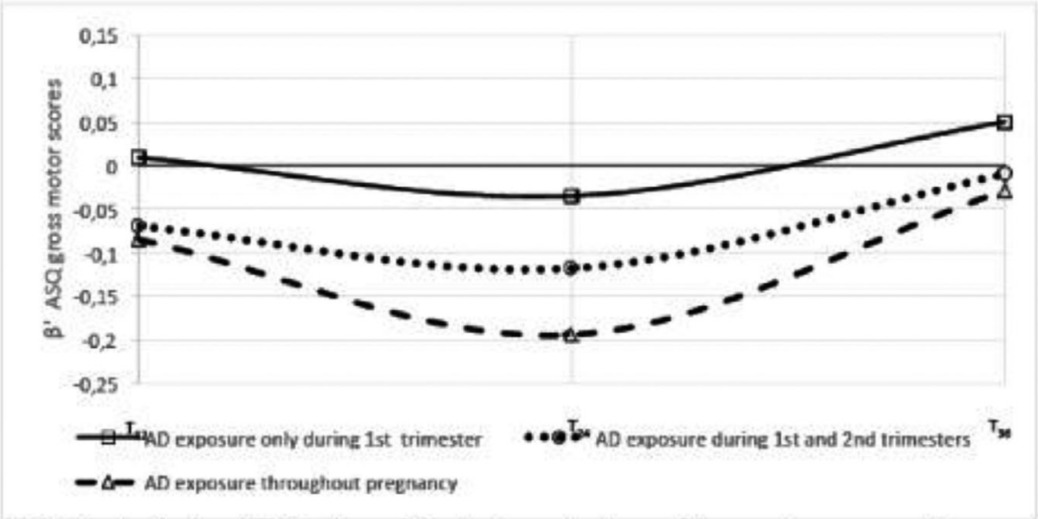

**Fig.3b** Standardized and adjusted association between in-utero antidepressant exposure patterns and **GROSS MOTOR scores** assessed with the Ages and Stages Questionnaire (ASQ) compared to non-exposed group.

*$p = 0.0036$; $p > 0.05$ for all other comparisons.

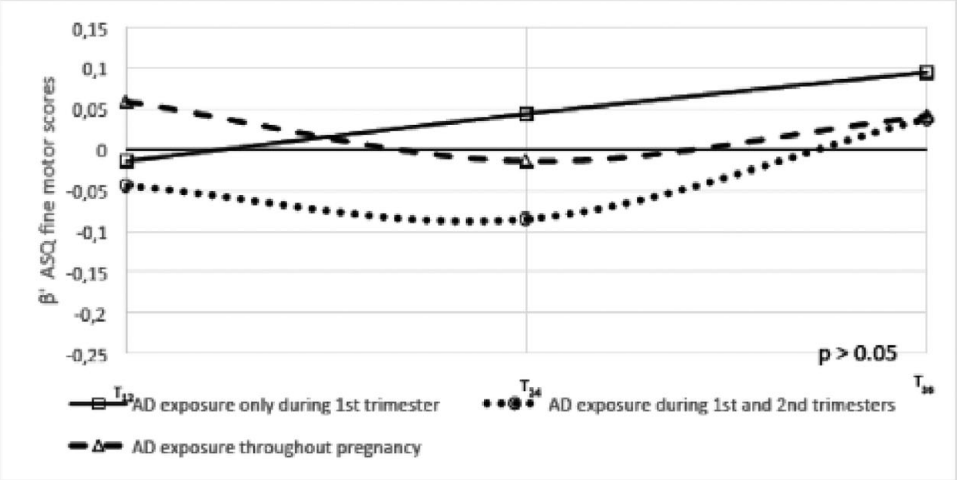

**Fig.3c** Standardized and adjusted association between in-utero antidepressant exposure patterns and **FINE MOTOR scores** assessed with the Ages and Stages Questionnaire (ASQ) compared to non-exposed group.

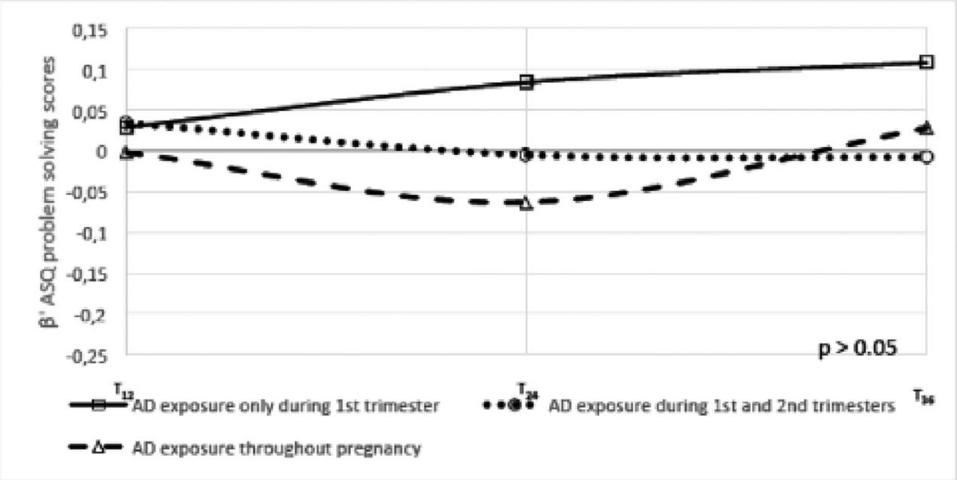

**Fig.3d** Standardized and adjusted association between in-utero antidepressant exposure patterns and **PROBLEM SOLVING** assessed with the Ages and Stages Questionnaire (ASQ) compared to non-exposed group.

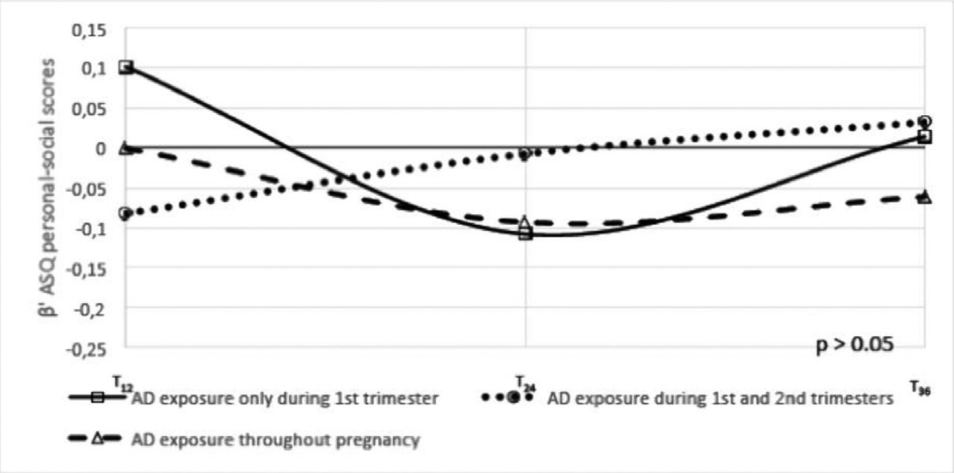

**Fig.3e** Standardized and adjusted association between in-utero antidepressant exposure patterns and **PERSONAL-SOCIAL scores** assessed with the Ages and Stages Questionnaire (ASQ) compared to non-exposed group.

MotherToBaby teratology information service ((a) US - Texas, Utah, New York, Arizona, California, Connecticut; Illinois; and (b) in Canada - Quebec, Ontario) during 2006-2010, be 2) ≥18 years old, 3) ≤14 weeks of pregnancy, 4) exposed to an AD (users) or any exposure considered non-teratogenic (non-users) at the time of the call. Women were excluded if they were exposed to a known teratogen. Socio-demographic and lifestyles, and medical history were collected during each trimester of pregnancy and annually until 3 years after birth. Mothers annually reported children's development by filling out Age-and-Stage questionnaire, and validated measures of maternal depression (EPDS) were collected. Exposure to AD was reported by mothers, and defined by trimester, type, dosage.
**Results:** Of 364 infants included, 10 (3%) were exposed to AD solely during the 1st trimester, 24 (7%) during the 1st/2nd trimesters, 181 (50%) throughout pregnancy, the remaining were unexposed. Adjusting for covariates, in-utero exposure to AD had no effect on cognitive function at 3 years old.
**Conclusions:** In-utero patterns/trajectories of AD use during pregnancy did not have an impact on children's cognitive function at 3 years old compared to non-use. Assessments at later life stages are needed.

### O0120

### Sex-specific volumetric variations associated with alcohol use in adolescence: A longitudinal neuroimaging study

X. Navarri*, I. Filippi and P. Conrod
Canada

**Introduction:** There is growing evidence that alcohol use (Thayer et al., 2017) during adolescence is associated with brain volumetric alterations. These variations can be interpreted as neurotoxic consequences of alcohol use or common vulnerabilities in cross-sectional studies. Moreover, little is known about the sex-specific volumetric abnormalities that could predate alcohol use and thus present markers of vulnerability.
**Objectives:** The current longitudinal study aims to determine whether sex-specific variations in adolescence predate and predict alcohol use.
**Methods:** Participants from the Neuroventure cohort were recruited at 12 years old (n = 155) to measure the correlates of adolescent drinking on the developing brain structure (Bourque et al., 2016). Data was collected at three time points when the participants were 12, 14 and 16 years old. Alcohol use was assessed using the DEP-ADO questionnaire. We used the Freesurfer longitudinal neuroimaging pipeline of the ENIGMA consortium to extract individual subcortical volumes and cortical thickness. We performed linear mixed-effect models for each regions of interest.
**Results:** Preliminary results indicates that alcohol use is associated with reduced volumes in a priori selected regions with the main sex effect considered in the model. Sex-by-time interaction are being explored.
**Conclusions:** Conclusion: The sex-specific volumetric variations observed in adolescents could predate and predict at-risk alcohol use. A better understanding of the sex-specific variations could lead to more targeted intervention programs.

## ORAL COMMUNICATION SESSION 12: GENETICS & MOLECULAR NEUROBIOLOGY - PART II / PSYCHONEUROIMMUNOLOGY / RESEARCH METHODOLOGY

### O0123

#### Exploring epigenetic mechanisms linking childhood adversity and psychosis in patients with first episode of psychosis – data from the EUGEI study

L. Alameda*, M. Aas, V. Rodríguez, R. Kandaswamy, D. Quattrone, C. Wong, C. Gayer-Anderson, C. Morgan, M. Di Forti and R. Murray

United Kingdom

**Introduction:** Epigenetics is emerging as an important player underlying the interactions between genetic and environmental risk factors in the aetiology of psychiatric disorders. We could hypothesize that epigenetic changes related to childhood adversity (CA) contribute to the underlying mechanism linking CA and psychosis.
**Objectives:** We aim to explore, whether CA leads to DNA-methylation changes at the level of EWAS in First Episode of Psychosis (FEP) patients. We will also examine whether these changes in DNA-methylation mediate the link between CA and psychosis.
**Methods:** We used EWAS profiling using the Illumina Infinium Methylation EPIC array in human peripheral blood tissue from 413 FEP and 521 healthy population controls part of the EUGEI study. Polyvictimisation scores were created with the Childhood Trauma Questionnaire (CTQ) ranging from 0-5. Linear regression models at an EWAS level and subsequent mediation analyses were perfomed, adjusting by a broad range of confounding factors.
**Results:** Preliminary results showed that polyvictimisation scores were significantly associated to the case control status; in cases, EWAS analyses showed that polyvictimization was significantly associated (p<5x10-5) with DNA methylation in 82 probes, located in 48 genes, some of these involved in pathways such as extracellular matrix, neural development or the kynurenine pathway, among other important processes. In controls, DNA methylation changes in such probes did not appear to be associated with polyvictimisation.
**Conclusions:** Our results show that in FEP, polyvictimisation leads to epigenetic modifications in the form of DNA-methylation in genes previously described in psychosis aetiopathogenesis. Mediation analyses will allow to determine whether changes in these probes mediate the link between adversity and psychosis.

### O0124

#### Enriched developmental biology molecular pathways impacts on antipsychotics induced weight gain

A. Drago* and H. Corfitsen

Denmark

**Introduction:** Psychotropic induced weight gain (PIWG) may lead to increased risk for cardiovascular diseases, metabolic disorders and, ultimately, treatment discontinuation. PIWG may be genetically driven. The unravelling of the genetics of PIWG may help identifying the subjects at risk for them.
**Objectives:** The hypothesis tested in the present contribution was that PIWG may have a signfiicant genetic compoent, especially when investigated through consistent molecular pathways. The analysis of complete molecular pathways granted a sufficient power to tackle the biologic variance and complexity of PIWG.
**Methods:** A genetic sample from the CATIE trial (n=765; M=556, mean age = 40.93±11.03) - a multi-phase randomized controlled trial of antipsychotic medications involving 1,460 persons with schizophrenia followed for up to 18 months - was investigated. A molecular pathway analysis was conducted. A genome-wide analysis with standard quality procecudres was input for the analysis through Bioconductor and ReactomePA. Correction for multiple testing (Bonferroni, FDR) and 10E5 permutations (through creation of random molecular pathways) was implemented.
**Results:** The developmental biology molecular pathway was significantly (p.adj= 0.018) enriched in genetic variations significantly (p<0.01) associated with PIWG. The pathway is involved in the regulation of beta-cell development, and the transcriptional regulation of white adipocyte differentiation.
**Conclusions:** Results correlate with previous evidence and is consistent with our earlier result on the STAR*D sample. The involvement of the beta-cell development and the trasnscriptional regulation of white adipocyte differentiation pathways stresses the relevance of the peripheral tissue rearrangement, rather than increased food intake, in the biologic modifications that follow psychotropic treatment and may lead to PIWG.

### O0126

#### Copy number variation of satellite III (1Q12) in patients with schizophrenia

M. Kaydan*, N. Zakharova, E. Ershova, G. Shmarina, O. Agafonova, L. Bravve, E. Jestkova, A. Martynov, R. Veiko, P. Umriukhin, G. Kostyuk, S. Kutsev, N. Veiko and S. Kostyuk

Russian Federation

**Introduction:** Human satellite DNA organized in long arrays in peri/centromeric heterochromatin. Copy number variants (CNVs) of human satellite III (1q12) fragment (f-SatIII) reflects the human cells response to oxidative stress (OS). With the action of OS, there is a significant disproportionation of people in the content of f-SatIII in leukocytes. OS has been proposed as one of the possible reasons of schizophrenia (SZ). Thus, it is possible to suggest a significant disproportionation of the SZ leukocytes in the content of f-SatIII.
**Objectives:** The definition of CNVs of the f-SatIII in the leukocytes of SZ patients and healthy control.
**Methods:** Biotinylated pUC1.77 probe was used for f-SatIII quantitation in leukocyte DNA by the non-radioactive quantitative hybridization for SZ patients (N = 840) and healthy control (HC, N=401).
**Results:** f-SatIII in human leukocyte varies between 5.7 to 44 pg/ng DNA. F-SatIII content in SZ group was significantly reduced compared HC-group ($p < 10^{-30}$). f-SatIII content in SZ patients negatively correlated with the index reflecting the seriousness of the disease (PANSS). The SZ (medicated) and SZ (drug-naïve) subgroups do not differ in the content of f-SatIII.

**Conclusions:** The content of f-SatIII in leukocyte of the SZ patients is significantly reduced compared to HC. It can be assumed that the low content of f-SatIII in the DNA of SZ patients is a genetic feature of this disease. The Russian Science Foundation (Grant No. 18-15-00437) supported this research.

### O0127

### Cytokine levels in schizophrenia patients with metabolic syndrome

A. Boiko*, I. Mednova, E. Kornetova and S. Ivanova
Russian Federation

**Introduction:** The incidence of metabolic syndrome (MetS) in schizophrenia is two times higher than in the general population. It has been suggested that the effects of inflammatory mediators causally contribute to the pathology of schizophrenia and metabolic disorders.
**Objectives:** The aim of the study is to measure cytokines in schizophrenia patients with and without MetS.
**Methods:** After obtaining informed consent, 136 patients with schizophrenia (ICD-10: F20) and 53 healthy persons were included. Patients were divided into two groups: 48 with MetS and 88 without it(IDF-criteria). Concentrations of cytokines (IL-1α, IL-1β, IL-2, IL-3, IL-4, IL-5, IL-6, IL-7, IL-8, IL-9, IL-10, IL-12(p40), IFNα2, IFNγ, TNFα, PDGF-AA, Flt3L, PDGF-AB/BB, IL-1RA, VEGF, RANTES, Fractalkine, FGF-2, Eotaxin, GRO) were measured on an analyzer MAGPIX (Luminex, USA).
**Results:** Analysis of the cytokines revealed a number of deviations between patients and healthy persons, as well as patients with and without MetS. Concentrations of IL-7, FGF-2, IFNα2, IL-2, IL-3, VEGF, RANTES were higher and concentration of IL-10 was lower in healthy persons compared with patients ($p_1$=0.01, $p_2$=0.043, $p_3$=0.042, $p_4$=0.019, $p_5$=0.037, $p_6$=0.03, $p_7$=0.043, $p_8$=0.032). Concentrations of Flt3L and IL-10 were higher and levels of FGF-2, IL-2 and PDGF-AB/BB were lower in patients with MetS compared with patients without MetS ($p_1$=0.007, $p_2$=0.036, $p_3$=0.023, $p_4$=0.004, $p_5$=0.027).
**Conclusions:** The results show a certain role of immunoinflammation in the pathogenesis of the MetS in schizophrenia. Further study of the mechanisms of metabolic disorders in schizophrenia is necessary to optimize the approach to diagnose and assess the risk of MetS. This study was supported by the RSF grant #18-15-00011.
**Disclosure:** This study was supported by the RSF grant #18-15-00011

### O0128

### Classification of first episode psychosis and chronic psychosis patients using the immunological transcriptome profile in a large cohort of patients: A machine learning analysis

P. Enrico*, G. Delvecchio, G. Colombo, N. Turtulici, F.M. Villa, A. Finardi, R. Furlan and P. Brambilla
Italy

**Introduction:** Current literature has described psychosis as a pathological condition affecting multiple system, including a relevant involvement of immune system, possibly varying in different stages of psychotic disorders.
**Objectives:** We carried out a Machine Learning (ML) analysis aiming at differentiating FEP patients, chronic psychosis patients and health controls, based on transcriptomic of genes linked to immune response and inflammation.
**Methods:** We extracted mRNA from peripheral mononucleate blood cells of a population of 348 subjects, including FEP patients (n=148), patients affected by chronic psychosis (CRO, n=65), and healthy controls (HC, N=129). Then, we performed a rtPCR to measure the mRNA levels of 56 genes involved in immune response, applying a ML classification algorithm basing on these.
**Results:** The results of ML analysis carried out in our sample predict the diagnosis of FEP, CRO and HC with a mean accuracy of 79.1%. Moreover, the total gain feature importance method reveals that the transcript levels of just 11 immunological genes are sufficient to predict the three groups reaching a total mean accuracy of 77.2%.
**Conclusions:** Our study is the first trying to classify different phases of psychotic disorders and healthy subjects with transcriptomic of immunological genes, by means of ML technology. The accuracy value resulting from our classification model strongly supports

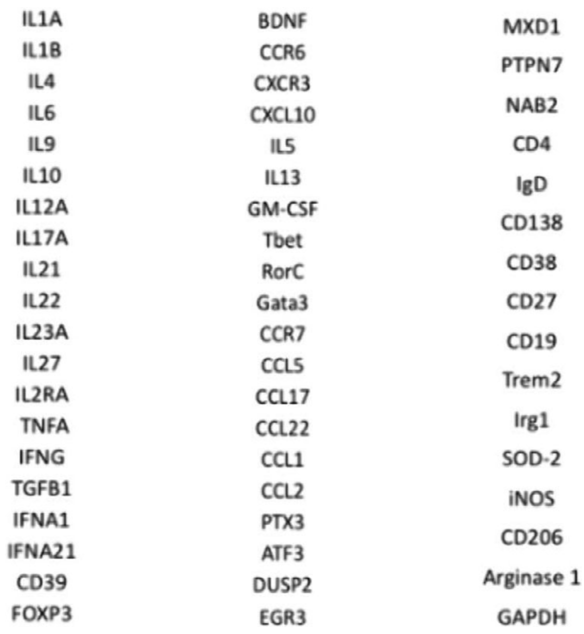

| | | |
|---|---|---|
| IL1A | BDNF | MXD1 |
| IL1B | CCR6 | PTPN7 |
| IL4 | CXCR3 | NAB2 |
| IL6 | CXCL10 | CD4 |
| IL9 | IL5 | IgD |
| IL10 | IL13 | CD138 |
| IL12A | GM-CSF | CD38 |
| IL17A | Tbet | CD27 |
| IL21 | RorC | CD19 |
| IL22 | Gata3 | Trem2 |
| IL23A | CCR7 | Irg1 |
| IL27 | CCL5 | SOD-2 |
| IL2RA | CCL17 | iNOS |
| TNFA | CCL22 | CD206 |
| IFNG | CCL1 | Arginase 1 |
| TGFB1 | CCL2 | GAPDH |
| IFNA1 | PTX3 | |
| IFNA21 | ATF3 | |
| CD39 | DUSP2 | |
| FOXP3 | EGR3 | |

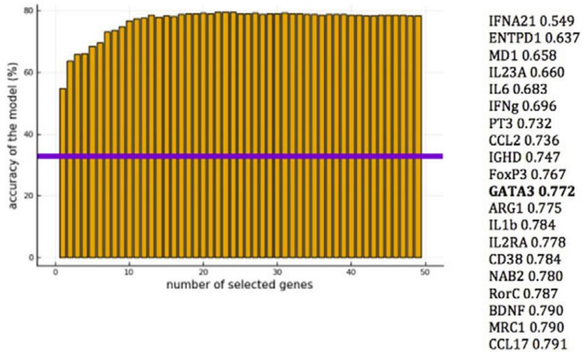

current evidence reporting the involvement of the immune system in the pathogenesis and course of psychotic disorders. Moreover, our results suggest the need to define an "immunological print" of subjects affected by psychosis, with the final aim of translating it in clinical practice.

### O0130

### Culturally equivalent translation into spanish of the 31-item adult ADHD self-report scale (ASRS)

E. Mertz-Echauri*, J. Bernacer, L. Gambra, S. Magallón, I. Garcia-Arbizu, M. Vallejo-Valdivielso and G. Arrondo
Spain

**Introduction:** Standardized screening rating scales, such as the Adult ADHD Self-Report Scale (ASRS), are recommended for the diagnostic process. The most recent and longest version has not been translated into Spanish.
**Objectives:** To translate the 31-item ASRS into Spanish.
**Methods:** Four independent translators conducted the initial forward translation, using previous WHO approved versions in Spanish as reference. The herein obtained version was pre-tested, debriefing participants to verify an equivalent understanding of the original meaning. An expert panel reviewed the observations afterwards.
**Results:** The independent translations were compared with a closer discussion of the last fourteen items. The resulting version was tested on 50 adults, aged 18-70. After being administered the scale, they were debriefed following a semi-structured interview. Difficulties understanding were mainly observed due to differing life situations of the sample, such as different employment status and age groups. The expert panel reviewed the results of the pretesting, and one item was changed to comply with the linguistic norms of Spain (Table 1). The total score range was 9-92 points; most individuals obtained total scores between 30 and 60 points (Figure 1).

Table 1. Selection of options for translations with reasons for change

| Expert panel's reasons | Items | Examples |
|---|---|---|
| Adequacy of expressions (mainly idioms) | 13, 14, 16, 17 | "relajarse" instead of "disfrutar de la tranquilidad" (cf. *Argentinian version*); "tiempo libre" instead of "tiempo para usted" (cf. *Argentinian version*) |
| Conceptual equivalence | 22 | "depende de los demás para mantener un orden en su vida" from "depend on others to keep your life in order" (cf. *English version*) |
| Semantic equivalence | 23-25, 28 | "completar tareas en el tiempo del que dispone" from "complete tasks in the allotted time" (cf. *English version*) |
| Most commonly used words | 19, 26 | "pierde el tiempo o hace un mal uso del mismo" from "waste or mismanage time" (cf. *English version*) |
| Concise reflection of content | 20, 21 | "en situaciones en las que es necesaria una planificación detallada" from "situation where planful behavior is needed" (cf. *English version*) |
| Consensus | 27, 29, 30, 31 | "molesto o abrumado que otra gente en su misma situación" from "hassled or overwhelmed than other people in your situation" (cf. *English version*) |

Figure 1. Histogram of total scores in the 31-item ASRS scale.

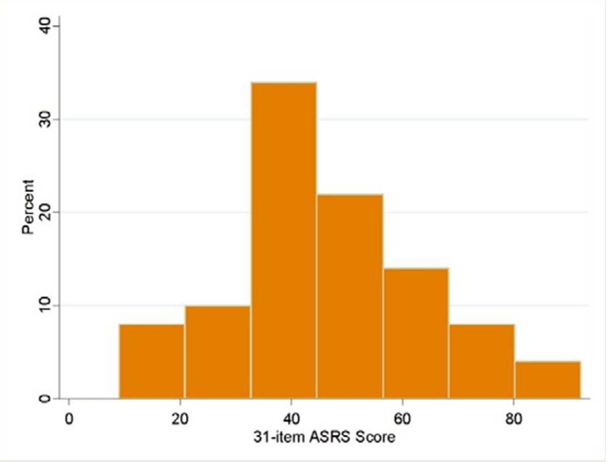

**Conclusions:** A culturally equivalent Spanish translation of the updated ASRS is presented.

### O0131

### Reactivity intensity polarity and stability scale (RIPOST): Statistical validation of a literary arabic version

A. Allaya*, A. Braham, H. Ben Rejeb, W. Chebbi, J. Nakhli and S. Ben Nasr
Tunisia

**Introduction:** Emotional dysregulation is a heterogeneous concept with a significant relevance in psychiatric research and clinical practice. E. Hantouche developed the Reactivity Intensity Polarity and Stability scale (RIPOST) to evaluate the different dimensions of the emotions. It was only recently that it was validated in its original language.
**Objectives:** The main purpose of this study was to evaluate and validate the psychometric properties of the Arabic version of the RIPoST scale.
**Methods:** The RIPoST scale is composed of 60 items, which are grouped in 4 dimensions. The questionnaire was translated using the forward /backward translation method. The validity of the construct was effectuated by principal component analysis (PCA) with 100 bipolar patients aged between 18 and 70 years old, in remission for 2 months. To investigate discriminative validity, we proceeded by an analysis of the variance and a Post-Hoc test between our patients, 50 healthy siblings and 50 healthy subjects recruited randomly among blood donors. Cronbach's alpha was used to estimate reliability.
**Results:** Factorial analysis using the PCA required 6 successive analyses after which 26 items were rejected and 34 items, with a correlation superior to 0, 5, were retained. The RIPoSt scale demonstrated good discriminative validity and an acceptable internal consistency for the RIPoSt total score with a Cronbach's alpha of 0,937 and for every single subscale (ranging from 0,686 to 0,913).

**Conclusions:** Our study joins others into the validation of the RIPoST scale. Our Arabic version seems to be a pertinent tool in the research field and in the clinical practice.

### ORAL COMMUNICATION SESSION 13: POSTTRAUMATIC STRESS DISORDER / SUICIDOLOGY AND SUICIDE PREVENTION / ONCOLOGY AND PSYCHIATRY

#### O0132

#### Oral communication session 13: Posttraumatic stress disorder / suicidology and suicide prevention / oncology and psychiatry

K. Grunberg*, J. Stoltenberg and C. Miller
United States of America

**Introduction:** Post-Traumatic Stress Disorder (PTSD) is a debilitating psychiatric disorder that develops following a traumatic stressor. Primary functional magnetic resonance imaging (fMRI) studies have partially illuminated the underlying neuropathology of PTSD; however, limited power and conflicting results from these studies indicate the need for a quantitative summary of this growing body of literature.
**Objectives:** This study seeks to identify the most reliable neural abnormalities of PTSD identified in the functional neuroimaging literature and build a whole-brain, neural representation of task-based hyperactivities and hypoactivities.
**Methods:** We conducted the largest to-date meta-analysis of existing fMRI studies (N = 44) that compare PTSD patients (N = 723) to age-matched healthy controls (N = 813). We also used multilevel kernel density analysis and a voxelwise, whole-brain approach in order to search for abnormalities comprehensively throughout the brain and across multiple significance levels.
**Results:** Patients with PTSD showed reliable ($\alpha = 0.05 - 0.001$) patterns of neural abnormalities compared to healthy controls. These include brain regions responsible for stimulus detection, affective arousal, memory consolidation, and emotion regulation.
**Conclusions:** These results may help better diagnose PTSD and further develop treatments such as transcranial magnetic stimulation that target specific brain regions.

#### O0135

#### Suicidal risk in mood disorders: Do different types of childhood adversity differentially predict suicidal ideation, planning, and attempts?

T. Cassis*, E. Young and N. Low
Canada

**Introduction:** Suicide risk is increased by 3-6-fold among people with mood disorders compared to the general population. Childhood adversity (CA) provides a risk of suicide attempt among people with mood disorders. However, little is known about how both the severity and perpetrator of the childhood adversity relates to suicidal risk in the context of mood disorders.
**Objectives:** To examine the association between types and severity of CA and lifetime suicidal thoughts (ST), plans (SP), and attempts (SA) among patients with mood disorders.
**Methods:** This cross-sectional, retrospective study of 241 adults from the McGill University Mood Disorders Program. Mood diagnoses (bipolar disorder type I or II, major depression) and information on ST, SP and SA were determined using the Structured Clinical Interview for DSM-IV Axis I Disorders. CA was assessed using the Childhood Experiences of Care and Abuse Questionnaire. Logistic regressions adjusted for age, sex, and mood disorder type were conducted.
**Results:** ST was associated with the severity of both paternal neglect and role reversal. SP was associated with the exposure and severity of maternal antipathy and maternal psychological abuse, and the exposure to role reversal. SA was associated with the exposure and severity of paternal psychological abuse and the exposure to role reversal.
**Conclusions:** Among people with mood disorders, risk factors for ST, SP and SA may diverge qualitatively not only in terms of type and perpetrator of CA but also in terms of dose response. Role reversal was associated with suicidality, and should be considered in psychiatric care and future research among people with mood disorders.

#### O0136

#### Suicidal ideation and suicide attempts in obsessive-compulsive related disorders (OCRDs): A systematic review and meta-analysis

L. Pellegrini[1]*, N. Fineberg[2], M. Menchetti[1], D. Berardi[1], P. Rucci[1], G. Maina[1], Z. Rihmer[3] and U. Albert[1]
[1]Italy; [2]United Kingdom and [3]Hungary

**Introduction:** Recent systematic reviews show that OCD is at risk of suicide, but less is known about suicidality and other DSM-5 Obsessive-Compulsive Related Disorders (OCRDs).
**Objectives:** The present meta-analysis has the following aims: 1. to calculate the pooled prevalence rates of suicide attempts and current/lifetime suicidal ideation in OCRDs, and 2. to identify predictors of suicide risk.
**Methods:** This systematic review and meta-analysis was performed by searching in Medline from the date of the first available article to December 31, 2018. Meta-analyses of proportions based on random-effects Der Simonian and Laird method was used to derive the pooled estimates. Analyses were executed using Stata version 15.1.
**Results:** Concerning suicide attempts in BDD, the pooled prevalence rate is 0.287 (IC95% 0.202-0.372) (figure-1); comorbid OCD, substance use, MDD, poor insight, severity, early onset and muscle dysmorphia raise the risk. Pooled prevalence rates of current/lifetime suicidal ideation are 0.311 (IC95% 0.212-0.410) (subgroup A figure-2) and 0.742 (IC95% 0.694-0.790) (subgroup B figure-2), respectively. Hoarding Disorder has a pooled prevalence rate of suicide attempts of 0.176 (IC 95% 0.117-0.235) (figure-3), while current suicidal ideation is 0.105 (IC95% 0.072-0.137). Suicide attempts in Grooming Disorders (Trichotillomania and Skin Picking Disorder)

| Studies | Estimate (95% C.I.) | Ev/Trt |
|---|---|---|
| De Brito et al. 2015 | 0.039 (0.008, 0.069) | 6/155 |
| Rief et al. 2006 | 0.071 (0.000, 0.149) | 3/42 |
| Phillips et al. (A) 2007 | 0.133 (0.034, 0.233) | 6/45 |
| Fontanelle et al. 2006 | 0.150 (0.000, 0.306) | 3/20 |
| Zimmerman & Mattia 1998 | 0.188 (0.000, 0.379) | 3/16 |
| Albertini & Phillips 1999 | 0.212 (0.073, 0.352) | 7/33 |
| Buhlmann et al. 2010 | 0.222 (0.101, 0.344) | 10/45 |
| Costa et al. 2012 | 0.239 (0.159, 0.319) | 26/109 |
| Veale et al. 1996 | 0.240 (0.122, 0.358) | 12/50 |
| Bjornsson et al. (B) 2013 | 0.246 (0.192, 0.300) | 60/244 |
| Bjornsson et al. (A) 2013 | 0.250 (0.187, 0.313) | 46/184 |
| Philipps et al. 2005 | 0.275 (0.213, 0.337) | 55/200 |
| Hart et al. (B) 2013 | 0.278 (0.196, 0.360) | 32/115 |
| Hart et al. (A) 2013 | 0.294 (0.223, 0.364) | 47/160 |
| Phillips et al. (B) 2007 | 0.400 (0.248, 0.552) | 16/40 |
| Kelly et al. 2015 | 0.583 (0.304, 0.862) | 7/12 |
| Grant et al. 2002 | 0.625 (0.388, 0.862) | 10/16 |
| Conroy et al. 2008 | 0.938 (0.819, 1.000) | 15/16 |
| **Overall (I^2=94.62 % , P< 0.001)** | **0.287 (0.202, 0.372)** | **364/1502** |

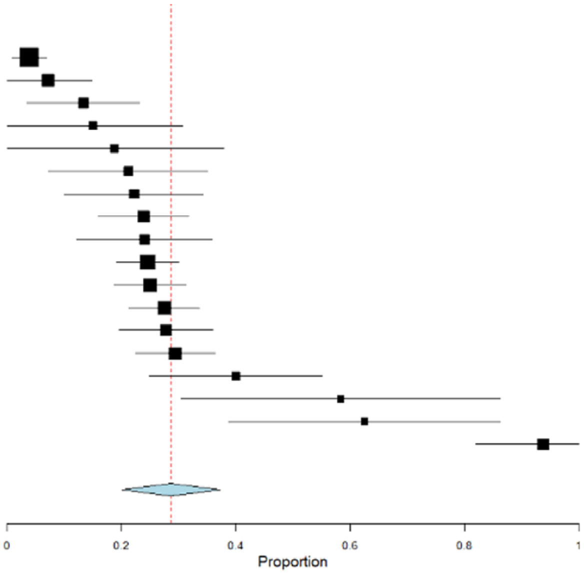

| Studies | Estimate (95% C.I.) | Ev/Trt |
|---|---|---|
| De Brito et al. | 0.090 (0.045, 0.135) | 14/155 |
| Rief et al. | 0.190 (0.072, 0.309) | 8/42 |
| Frare et al. (a) | 0.265 (0.116, 0.413) | 9/34 |
| Frare et al. (b) | 0.292 (0.110, 0.474) | 7/24 |
| Schieber et al. | 0.306 (0.192, 0.421) | 19/62 |
| Buhlman et al. | 0.311 (0.176, 0.446) | 14/45 |
| Fontanelle et al. | 0.350 (0.141, 0.559) | 7/20 |
| Moolman et al. | 0.364 (0.079, 0.648) | 4/11 |
| Phillips & Kelly | 0.388 (0.271, 0.505) | 26/67 |
| Perugi et al. | 0.448 (0.320, 0.576) | 26/58 |
| Altamura et al. | 0.500 (0.321, 0.679) | 15/30 |
| **Subgroup A (I^2=85.4 % , P=0.000)** | **0.311 (0.212, 0.410)** | **149/548** |
| Costa et al. | 0.532 (0.438, 0.626) | 58/109 |
| Albertini & Phillips | 0.667 (0.506, 0.828) | 22/33 |
| Kelly et al. | 0.667 (0.400, 0.933) | 8/12 |
| Hart et al. (b) | 0.739 (0.659, 0.819) | 85/115 |
| Phillips et al. (a) | 0.778 (0.656, 0.899) | 35/45 |
| Phillips et al. | 0.780 (0.723, 0.837) | 156/200 |
| Hart et al. (a) | 0.781 (0.717, 0.845) | 125/160 |
| Bjornsson et al. (a) | 0.772 (0.711, 0.832) | 142/184 |
| Bjornsson et al. (b) | 0.754 (0.700, 0.808) | 184/244 |
| Phillips et al. (b) | 0.850 (0.739, 0.961) | 34/40 |
| **Subgroup B (I^2=67.92 % , P=0.001)** | **0.742 (0.694, 0.790)** | **849/1142** |
| **Overall (I^2=97.45 % , P=0.000)** | **0.518 (0.390, 0.646)** | **998/1690** |

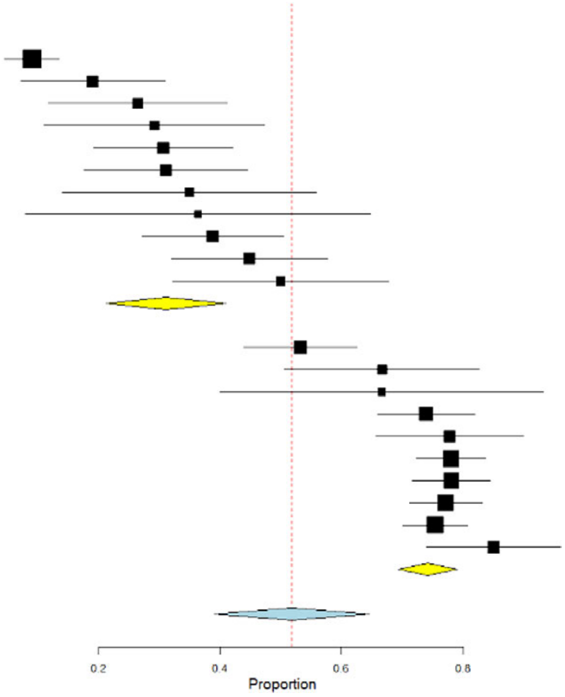

| Studies | Estimate (95% C.I.) | Ev/Trt |
|---|---|---|
| Alonso et al. 2010 | 0.065 (0.003, 0.126) | 4/62 |
| Torres et al. 2012 | 0.127 (0.099, 0.155) | 67/528 |
| Torres et al. 2011 | 0.131 (0.093, 0.170) | 39/297 |
| Archer et al. 2018 | 0.192 (0.148, 0.235) | 60/313 |
| Chakraborty et al. 2012 | 0.400 (0.185, 0.615) | 8/20 |
| Matsunaga et al. 2010 | 0.407 (0.276, 0.538) | 22/54 |
| **Overall (I^2=85.23 % , P< 0.001)** | **0.176 (0.117, 0.235)** | **200/1274** |

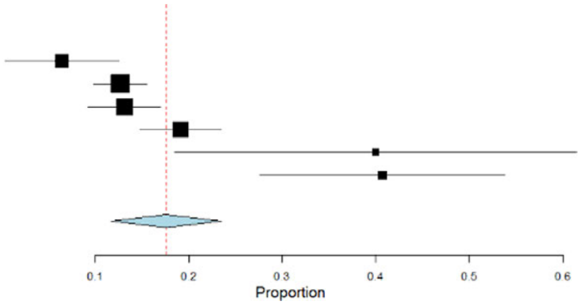

have a pooled prevalence rate of 0.115 (IC95% 0.054-0.176), current ideation in these disorders is 0.408 (IC95% 0.360-0.455).

**Conclusions:** Our present meta-analysis shows that, like pure OCD, OCRDs are at suicide risk independently from comorbid disorders (specifically independently from comorbid OCD); BDD is the disorder with the highest risk (even higher than OCD), followed by HD and then Grooming Disorders.

**Conflict of interest:** No

### O0137

### Suicidal ideation and suicide attempts in patients with obsessive-compulsive disorder: A systematic review and meta-analysis

L. Pellegrini[1]*, N. Fineberg[2], G. Maina[1], P. Rucci[1] and U. Albert[1]
[1]Italy and [2]United Kingdom

**Introduction:** Despite recent systematic reviews showed that OCD is at risk of suicide, a huge variability among results was found.

**Objectives:** The present meta-analysis has the following aims: 1. to calculate the pooled prevalence rates of suicide attempts and current/lifetime suicidal ideation in OCD, and 2. to identify socio-demographic and clinical factors associated with greater risk.

**Methods:** This systematic review and meta-analysis was performed by searching in four databases (Medline, PsycINFO, WoS, CINHAL) from the date of the first available article to June 20, 2019. Meta-analyses of proportions based on random-effects model was used to derive the pooled estimates. Subgroup analyses, univariate and multivariable metaregressions and leave-one-out analyses were performed. All statistical analyses were executed using Stata version 15.1.

**Results:** 61 independent studies were identified. The pooled prevalence rate of suicide attempts was 0.13 (95% CI 0.12-0.15) (figure-1). Univariate meta-regression analyses showed that increased rates were associated with substance use and depression severity (MADRS). In multivariable meta-regression, YBOCS

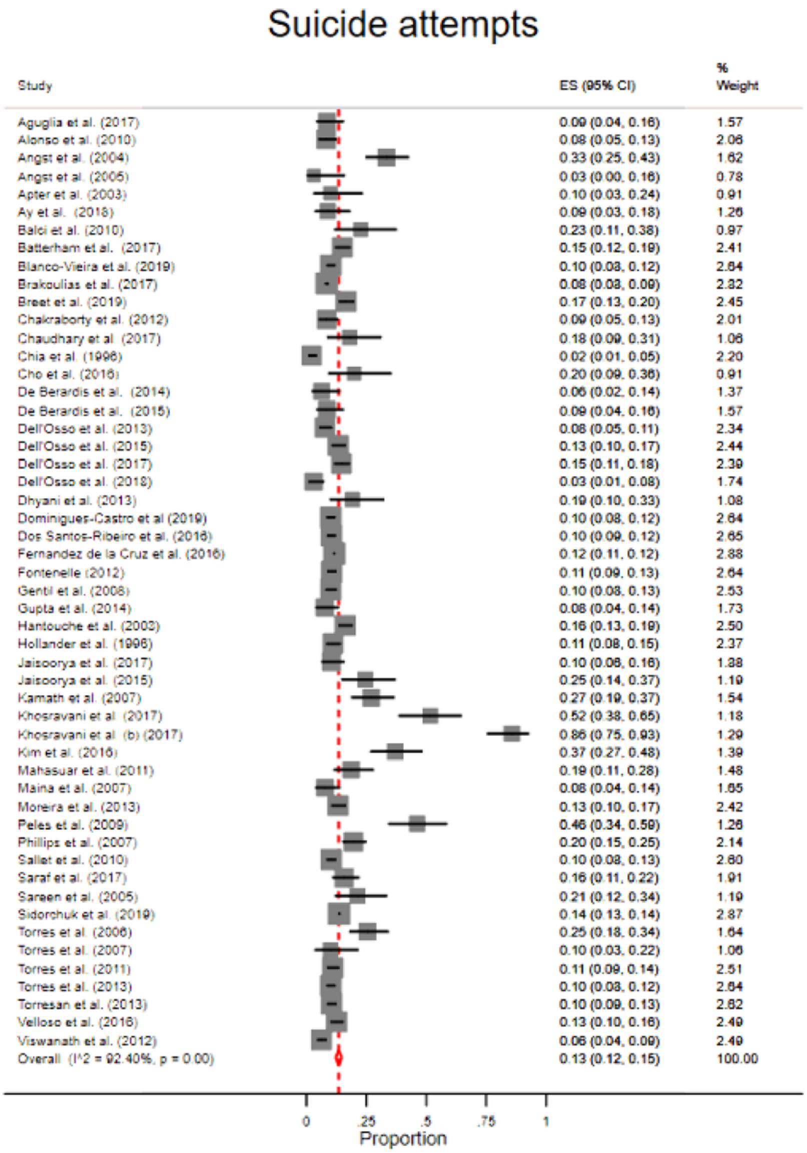

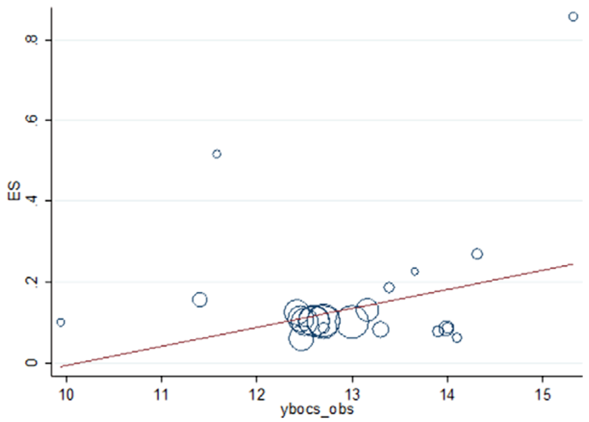

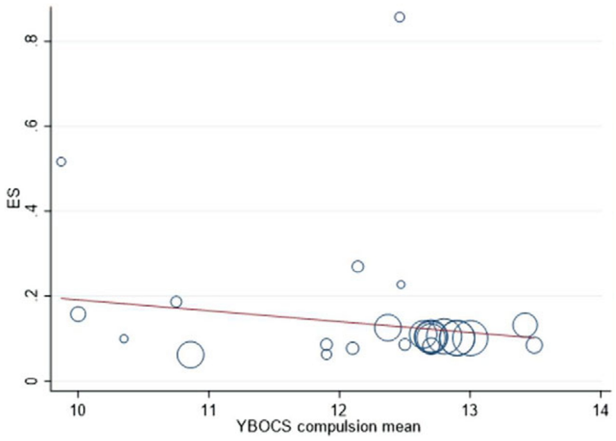

obsessions had a positive association with suicide attempts (figure-2), while YBOCS compulsions a negative one (figure-3). The pooled prevalence rates of current/lifetime suicidal ideation were 0.27 (95% CI 0.21-0.34) and 0.47 (95% CI 0.40-0.55), respectively. Variables such as lower education and YBOCS obsessions were positively associated with suicidal ideation, while YBOCS compulsions was negatively.
**Conclusions:** For the first time accurate estimates of prevalence of suicidality in OCD were provided; at least 1/10 patients attempted suicide, while nearly half had suicidal ideation. History and factors associated with greater risk should be closely investigated by clinicians in these patients.

### O0141

#### Longitudinal relations between insomnia and suicide attempts in adolescence and young adulthood

K. Alvarez*, M. Cruz Gonzalez, P. Bancalari, M. Alegria and R. Roberts
United States of America

**Introduction:** Insomnia has been identified as a risk factor for adolescent suicidal behavior, often via clinical and cross-sectional studies. Longitudinal associations between insomnia and suicidal behavior have not been well-established in adolescent community samples, and many studies fail to control for the effect of depressive symptoms and their overlap with both insomnia and suicidal behavior.
**Objectives:** We modeled longitudinal associations between insomnia and suicide attempts in a diverse community-based cohort of US adolescents interviewed at Wave 1 (n=4175; 11-17yo), W2 (+1yr; n=3134), and W3 (+5yrs; n=2503).
**Methods:** We estimated the association of W1 insomnia with W2 and W3 12-month suicide attempts, controlling for W1 attempts. We then estimated the inverse association between W1 attempts and W2 and W3 insomnia, controlling for W1 insomnia. Analyses used weighted survey logistic regression models, controlling for W1 depressive symptoms (excluding insomnia/suicidality) and socio-demographics.
**Results:** Weighted prevalence of any insomnia symptom ranged from 22.2%-26.8% across waves; insomnia with impairment ranged from 6.1%-8.1%. Any insomnia symptom at W1 was not associated with W2 attempts but significantly increased the odds of W3 attempts (OR=3.82). In contrast, insomnia with impairment at W1 was associated with W2 attempts (OR=3.14) but not W3. The inverse analysis showed no significant associations between W1 attempts and W2/W3 insomnia.
**Conclusions:** Presence of any insomnia symptom in adolescence may represent a distal risk factor for later suicide attempts, while insomnia with impairment may be a short-term risk factor. Preventive interventions to improve adolescent sleep, combined with clinical interventions for sleep disorders, may be an important multilevel suicide prevention strategy.

## ORAL COMMUNICATION SESSION 15: EATING DISORDERS / E-MENTAL HEALTH

### O0143

#### Evaluation of efficacy of social cognitive training in improvement of socioemotional processes throughout therapeutic process in an inpatients

E. Kot*, B. Kostecka, M. Wayda-Zalewska and K. Kucharska
Poland

**Introduction:** Social cognitive deficits (e.g. emotion perception, empathy, and theory of mind) seem to play crucial role in the symptoms persistence of anorexia nervosa (AN).
**Objectives:** Aim of study was to verify the therapeutic efficacy of cultural adaptation of Social Cognitive Training (SCT) in socio-emotional improvement of patients with AN.
**Methods:** Participants. The participants were 60 healthy controls (HCs) and 60 AN patients, examined at the beginning and at the end of 12-week-long hospitalization. Half of them was offered treatment as usual (TAU) while the other half –TAU + SCT. Measures. The clinical variables were measured using: The Rosenberg Self-Esteem Scale, The Eating Attitude Test, The Beck Depression Inventory, The State-Trait Anxiety Inventory, and The Toronto Alexithymia Scale (TAS-20). Social cognitive deficits were assessed using: Penn Emotion Recognition/Discrimination, and Acuity Tasks, Penn Facial Memory, and Reading the Mind in the Eyes Test (RME).

**Results:** At the beginning both groups of patients presented with high level of clinical psychopathology and social cognitive deficits which decreased significantly throughout therapy however most of the measured variables did not reach the level presented by HCs, apart from: sad recognition, reaction time for happy recognition, emotion discrimination time for happy and sad in TAU + SCT group. Overall, TAU + SCT group showed significantly higher improvement compared to the TAU group in: sad recognition, fear and anger recognition, sad discrimination, RT happy recognition, and ToM after treatment.

**Conclusions:** SCT appeared promising therapeutic tool in remediation of social cognitive functioning in AN. Project funded by National Science Centre NCN 2014/15/B/HS6/01847

**Conflict of interest:** No

**Keywords:** social cognition anorexia nervosa

### O0144

### The role of disgust sensibility and propensity in eating disorders patients with traumatic history

M. Innocenti*, V. Gironi, N. Giaquinta, L. Lucherini Bargellini Angeletti, F. Galassi, G. Castellini and V. Ricca

Italy

**Introduction:** Disgust has been identified to play a role in the development of several psychological disorders (1). Recent studies have tried to explore the relationship between disgust and eating disorders, with heterogeneous findings (2). Elevated levels of disgust are associated to traumatic events involving sexual victimization. One of the suggested mechanism implicated in this association is a feeling of mental contamination (3).

**Objectives:** The aim of this study is to investigate the relationship between disgust and history of maltreatment in patients with Eating Disorder. We also predict that levels of propensity and sensitivity to disgust in patients diagnosed with Eating Disorders and Anxiety Disorders are higher than healthy controls.

**Methods:** We enrolled 114 patients admitted in Psychiatric Unit of Careggi: 41 with diagnosis of Eating Disorder, 33 with Anxiety Disorders and 40 healthy controls. We administered to all groups: Childhood Trauma Questionnaire (CTQ), Eating Disorder Examination-2 (EDI-2), Disgust Propensity and Sensibility Scale-revised (DPSS-r).

**Results:** We noted higher scores at DPSS-r in the two psychopathological groups compared to the healthy group, in accordance with previous literature (barchart 1, table 1). We found a significant linear correlation between the DPSS-R scores and the CTQ subscales Emotional Abuse and Physical Neglect (table 2). Disgust Propensity was finally identified as a possible mediator of the relationship between Emotional Abuse and altered Interoceptive Awareness (Indirect Effect: 0,59; I.C. 95%: 0,30-0,95)

**Conclusions:** These preliminary data could suggest a possible role of childhood traumatic events in the development of Sensitivity and Propensity to Disgust in Eating Disorders patients.

**Conflict of interest:** No

Table 1

| | Control group | Anxiety Disorders | Eating Disorders | f | Sig. |
|---|---|---|---|---|---|
| **Disgust Sensitivity** | 14,47 ± 4,69 | 21,33 ± 5,21 | 19,70 ± 6,03 | 17,14 | 0.000 |
| **Disgust Propensity** | 19,90 ± 0,89 | 23,81 ± 4,96 | 25,53 ± 5,45 | 12.60 | 0.000 |

Table 2

| | Disgust Propensity (r) | Disgust Sensibility (r) |
|---|---|---|
| Emotional Abuse (CTQ) | 0,465 *p: 0,000* | 0,533 *p: 0,002* |
| Physical Neglect (CTQ) | 0,566 *p: 0,000* | 0,588 *p: 0,000* |

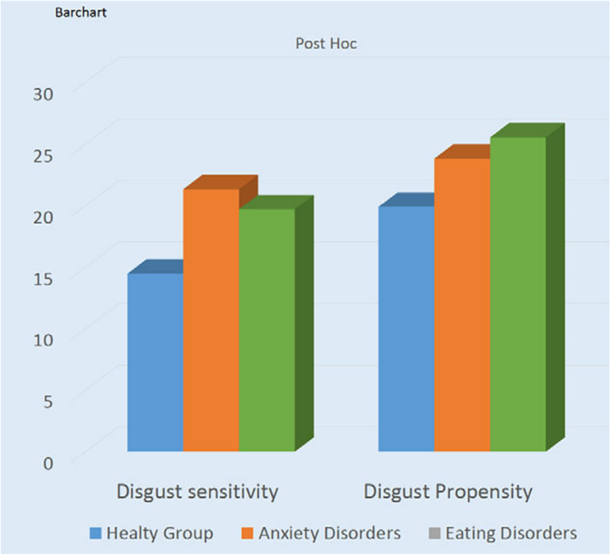

### O0145

### Impact of cognitive behavioral therapy on cortisol levels in patients with eating disorders and childhood abuse: A follow-up study

E. Cassioli*, E. Rossi, L. Lelli, G. Castellini, G. D'Anna, A. M. Monteleone and V. Ricca

Italy

**Introduction:** Childhood abuse is a significant risk factor for the development of Eating Disorders (EDs), and can induce changes in the hypothalamic-pituitary-adrenal (HPA) axis. The impact of cognitive behavioral therapy (CBT) on cortisol levels remains to be explored.

**Objectives:** To evaluate the effects of CBT on ED specific psychopathology, general psychopathology and blood cortisol levels in patients with EDs with and without a history of abuse.

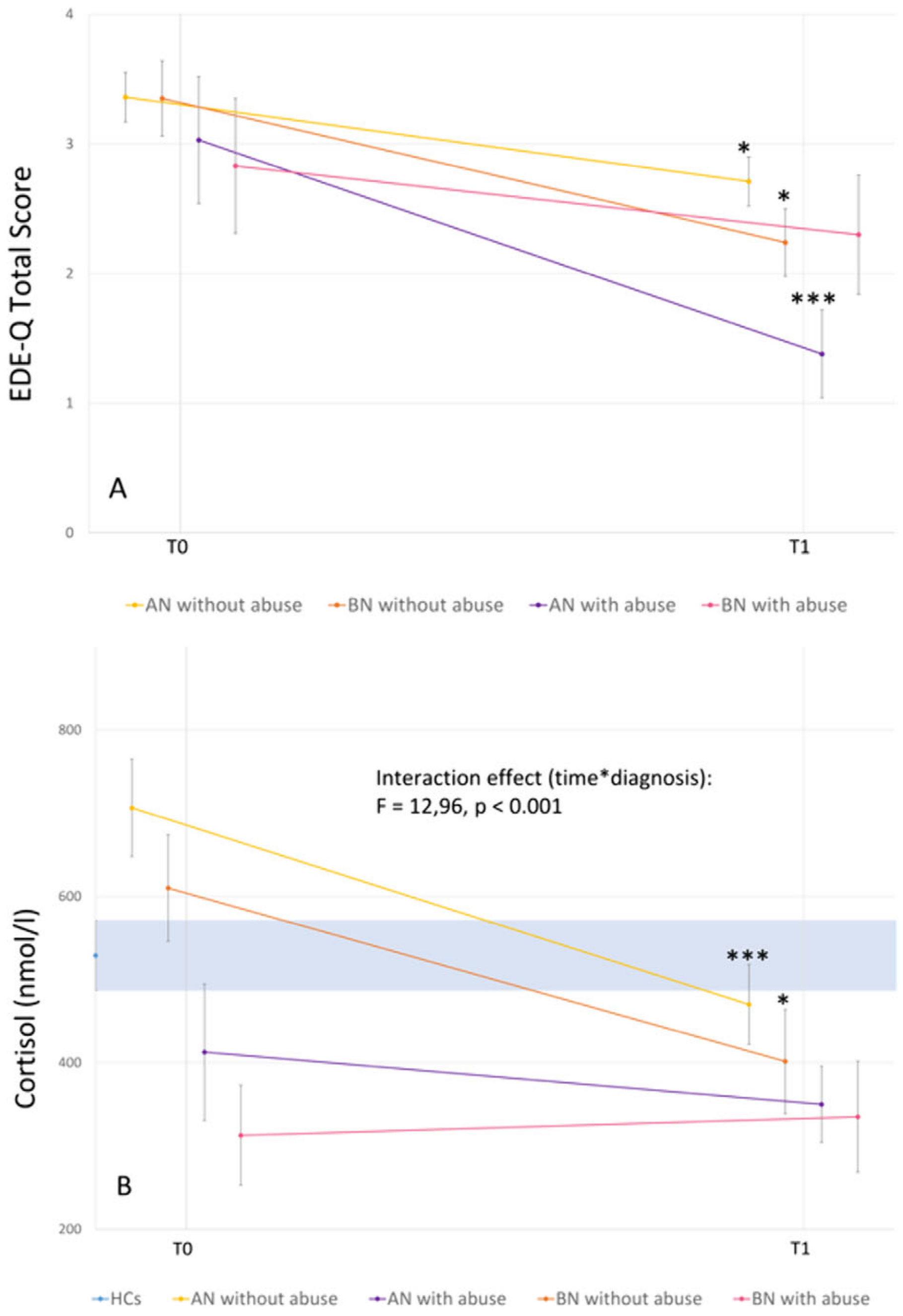

**Methods:** 74 female patients with anorexia nervosa (AN) and bulimia nervosa (BN) were treated with an individual CBT. At the first evaluation (T0) and at the end of the CBT (T1) psychometric tests were administered for the evaluation of ED specific (EDE-Q) and general psychopathology (SCL-90-R), and blood cortisol levels were measured. 76 healthy controls (HCs) were also recruited.
**Results:** Patients with a history of abuse showed lower cortisol levels than HCs, whereas non-abused subjects had higher levels. After CBT, EDE-Q scores were reduced in all patients except in the BN group with a history of abuse, while only patients without a history of abuse showed a reduction in SCL-90-R and cortisolemia.
**Conclusions:** Our study confirms the role of childhood abuse as a moderator of treatment response, in terms of lower effects on psychopathology and greater persistence of HPA axis alterations.

### O0146

### Genetic, neural and phenotypic predictors of eating disorder symptoms and comorbid mental health in adolescence and early adulthood

S. Desrivieres*, L. Robinson, Z. Zhang and U. Schmidt
United Kingdom

**Introduction:** Eating Disorders (EDs) are devastating mental disorders, with increasing prevalence. Without early identification and treatment EDs may run a chronic course.

**Objectives:** To characterise the development of disordered eating behaviours (DEBs) and related mental illnesses and identify early genetic, neural and psychopathological predictors of such trajectories.
**Methods:** Data were from the IMAGEN study, a large, prospective and longitudinal cohort of European adolescents assessed at ages 14, 16 and 19 years. For each self-reported DEB, we compared a developer group –consisting of participants who reported the DEB at 16 or 19 years, not at 14 years– to a control group in which participants did not report the DEB at any age. Grey matter volumes (GMVs), self-reports of emotional and behavioural problems, mental health symptoms, addiction-related behaviours and personality traits were investigated as predictors. Polygenic risk scores (PRS) for full neuroticism and its item clusters 'depressed affect' and 'worry', BMI and Attention Deficit Hyperactivity Disorder (ADHD) symptoms were calculated to investigate genetic contributions to DEBs.
**Results:** Differences in GMVs in frontal and subcortical brain regions, body mass index (BMI), neuroticism, impulse control behaviours and addiction at age 14 differentially predicted future eating behaviours and subsequent symptoms of depression and generalised anxiety. Genetic analyses revealed etiological overlaps between BMI, neuroticism-related depressed affect and attention-deficit hyperactivity disorder (ADHD) and dieting, binge-eating and purging, respectively.
**Conclusions:** Genetic predispositions and biopsychopathological processes related to obesity, impulse control and neuroticism may be useful early and differential biomarkers of the vulnerability for eating disorders in adolescence.

### O0147

### The validity of the 5th BMI percentile as weight cut-off for anorexia nervosa in children and adolescents: No evidence from a psychopathology investigation

G. Cascino*, A. Mereu, V. Zanna, P. Monteleone, S. Vicari and A. M. Monteleone
Italy

**Introduction:** Although the $5^{th}$ Body Mass Index (BMI) percentile has been indicated by the DSM-5 in order to identify underweight in children and adolescents and to diagnose anorexia nervosa (AN), the validity of this cut-off has not been proved.
**Objectives:** Aim of this study was to compare general and specific psychopathology between adolescents affected by AN with a BMI above or below the $5^{th}$ percentile in order to test the validity of this weight cut-off.
**Methods:** Four hundred-three adolescents with AN were recruited. They completed the Eating Disorder Inventory-3 (EDI-3) and the Youth Self Report (YSR). The sample was divided in two groups: below the $5^{th}$ (n = 172) and above the $5^{th}$ (n = 231). Quantitative differences between groups were investigated through t-test for independent samples. A network analysis was conducted in each group.
**Results:** The group with BMI above the $5^{th}$ percentile showed significantly higher scores in drive to thinness, body dissatisfaction, bulimia, personal and interpersonal alienation, social insecurity and asceticism. No significant differences emerged in general psychopathology measured through the YSR and in the occurrence of comorbidity between the groups. The group with BMI under the $5^{th}$ percentile had a longer illness duration. No difference emerged between the network structure of the two groups.
**Conclusions:** These findings suggest that the clinical utility of the $5^{th}$ BMI percentile as weight cut-off for diagnosis of AN in children and adolescents is questionable. Indeed, it may prevent early illness detection and intervention, which are essential to improve treatment outcome.

### O0148

### Maudsley learning podcast: The innovation, implementation and appraisal of a technological advance in psychiatric education - an accessible tool for both clinical and personal reflection

A. Curmi*
United Kingdom

**Introduction:** Digitalisation of global education is a reality which has profoundly shaped perceptions of mental health. Podcasts are set to change the way information is delivered, as studies have established they are a far-reaching and accessible vehicle for medical education.
**Objectives:** In collaboration with Maudsley Learning organisation, a series of podcasts were developed with a scope to: 1. Explore interdisciplinary working in mental health including psychiatry, psychology and philosophy. 2. Reach clinicians and non-clinicians. 3. Evaluate their effectiveness as an educational tool.
**Methods:** Two psychiatrists and one psychologist were interviewed regarding their areas of expertise. Podcasts were distributed online and promoted using social media. Listener frequency was monitored using an online hosting service. A survey was distributed to podcast listeners, including: 15 questions spanning demographics and impact on knowledge of specific topics (e.g.: prescribing, psychotherapy). Quantitative data was collected using the Likert scale and analysed using Microsoft Excel. Qualitative data was collected using open ended questions and analysed using NVivo statistical software.
**Results:** Total number of listens was n=320 with the most popular episode garnering 96 listens. Selected graphs represent current analyses of demographics (Image 1) and impact on knowledge (Image 2):
**Conclusions:** Podcasts are a versatile and cost-effective means of promoting education in psychiatry. Alongside structured teaching, they provide an opportunity to produce more nuanced and in-depth content in the form of expert interviews.They represent an exciting tool to impart knowledge to both a clinical and non-clinical audience.

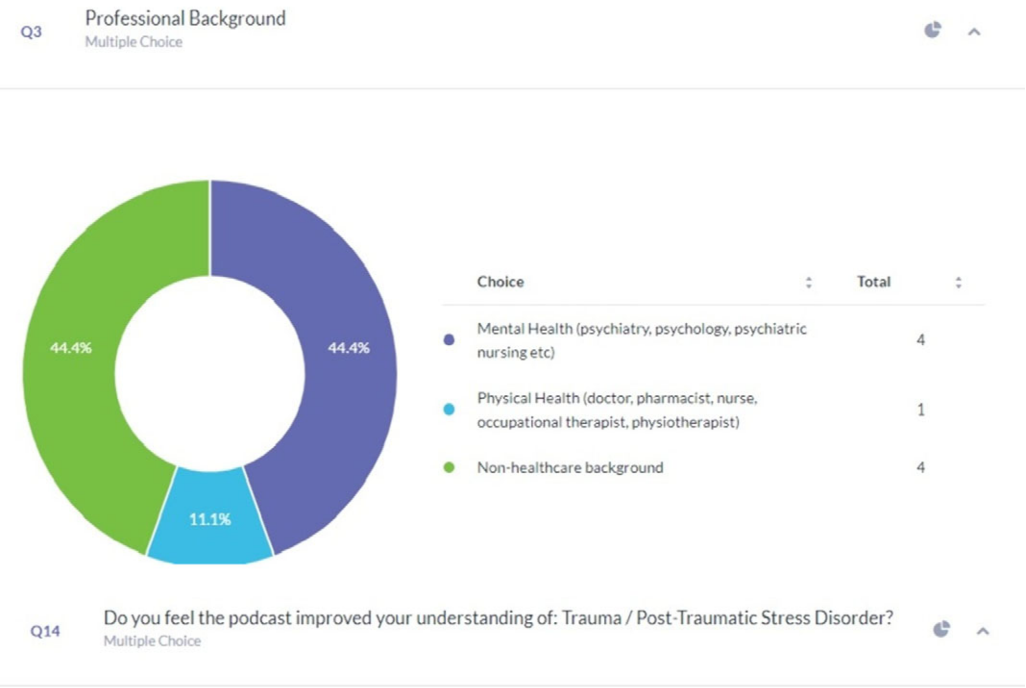

**Image 1** Professional background of respondents

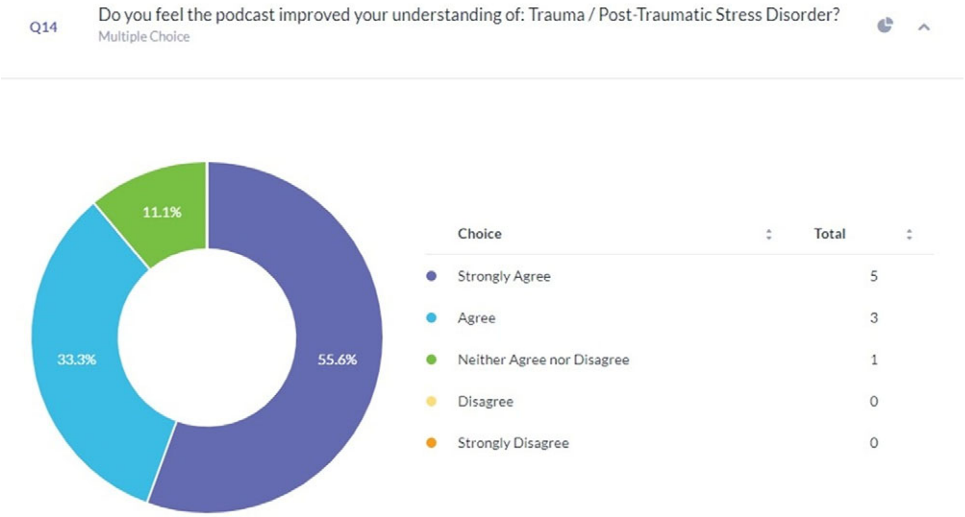

**Image 2** Impact of the podcast on PTSD knowledge for the respondents

### ORAL COMMUNICATION SESSION 14: OBSESSIVE-COMPULSIVE DISORDER / SLEEP DISORDERS & STRESS / DEPRESSIVE DISORDERS - PART II

#### O0150

#### Application of a staging model to a clinical sample of italian obsessive-compulsive patients

B. Benatti*, N. Girone, F. Fesce, G. Lucca, M. Vismara, V. De Carlo, B. Grancini, C. Donà, L. Casati, S. Ferrari, C. Virzì, A. Colombo, M. Bosi, C. Viganò and B. Dell'Osso

Italy

**Introduction:** Clinical staging is based on the concept that psychiatric illnesses progress over time through successive stages marked by symptoms of increased clarity and intensity: from increased risk, asymptomatic to severe illness. A clinical staging model integrating the severity of symptoms and the phase of illness for personalized case management for Obsessive Compulsive Disorder (OCD) patients has been proposed. Aim of the study is the application of this model to a sample of OCD Italian patients.

**Objectives:** Aim of the study is the application of this model to a sample of OCD Italian patients.

**Methods:** 188 patients with OCD were recruited and 32 patients completed a follow-up assessment. Patients were divided in four main stages (Stage 0: YBOCS 0 and family history for OCD; Stage 1: YBOCS 1-13; Stage 2: YBOCS 14-34; Stage 3: YBOCS 35-40) and then on 3 groups: no stage change, improvedstage or worsened stage between T0 and follow up.

**Results:** 53.1% of the followed-up patients showed no changes on their stage, 37.5% improvedtheir stage and 9.4% showed a progression tothe worse stage.All patients with a worsened stage showed at least one psychiatric comorbidity and a significantly higherprevalence of somatic obsessions (p<.05).The age at onset of patients witha worsened stage was earlier compared to the other groups and theduration of untreated illness andduration of illness resulted almost two times higher compared to the other groups
**Conclusions:** Preliminary results confirm the relevance of illness onset and early intervention in OCD. There is an emerging need of more stringent criteria for staging to better characterize OCD patients.

### O0151

#### Assessment of over valuated thoughts, metacognition, magical beliefs and quality of life in patients with obsessive compulsive disorder and subtypes

M.G. Teksin Bakir* and S. Aslan
Turkey

**Introduction:** Meta-cognitive thoughts are important predictors of severity, life quality and treatment response for obsessive compulsive disorder (OCD). Magical thoughts may affect the quality or quantity of obsessive beliefs in OCD patients. Occasionally obsessions are in form of Over Valuated Thought (OVT); and patients may be far from insight though they are not delusional, and aren't interpreting their obsessions always as egodistonic. Having obsessions characterized as OVT may lead to various clinical presentations and consequences.
**Objectives:** Our aim in is to evaluate the association of OVT's with metacognition and magical beliefs in OCD patients and subtypes, to compare with control and to evaluate the impact of these clinical features on severity of disorder and quality of life.
**Methods:** 54 OCD patients and 55 controls were included. According to the clinical interview, YBOCS and OVTS (Over Valuated Thought Scale), patients were divided into two groups as autogenous (n:23) and reactive (n:31). Magical Ideation Scale (MIS), Meta-cognition Scale (MCS), WHOOL-BREEF, Maudsley Obsessive Compulsive Questionnaire (MOCQL) and Beck Anxiety Inventory (BAI) scales were filled by all participants.
**Results:** For OCD patients MCS total scores, WHOQOL-BREF-TR and BAI total scores were significantly higher. WHOOL-BREEF score in OCD patients were found to be inversely related to metacognition scores and the severity of magical thoughts. Our hypothesis is confirmed given the significant correlation between severity of OCD, YBOCS, MCS and BDI total scores.
**Conclusions:** In OCD patients, higher level of metacognitive scores and OVT, related to severe magical beliefs, and poor quality of life.

### O0153

#### Transforming growth factor beta and childhood abuse in adults with major depressive disorder

A. Munjiza*, M. Kostic, Z. Kosutic, M. Mitkovic Voncina, D. Pesic, D. Todorovic, I. Markovic and D. Lecic Tosevski
Serbia

**Introduction:** Although the inflammatory hypothesis in depression is more than two decades old, only some aspects are well researched. Transforming growth factor beta (TGF-β), which has a role as an anti-inflammatory and regulatory cytokine, is very poorly investigated in patients with major depressive disorder (MDD) who experienced childhood trauma.
**Objectives:** Aim of our study was to analyze differences in serum concentrations of TGF-β between patients with MDD (with and without previous history of childhood abuse) and healthy controls, as well as to investigate possible predictors of TGF-β levels.
**Methods:** Blood samples were obtained from 55 patients who fulfilled DSM-IV-R criteria for a current MDD episode without psychotic symptoms and 45 healthy controls, matched for age and gender. Participants were administered the Beck Depression Inventory (BDI), Hamilton Depression Rating Scale (HDRS) and the Childhood Trauma Questionnaire (CTQ). Serum TGF-β concentration was determined by enzyme-linked immunosorbent assay.
**Results:** Concentration of TGF-β was significantly higher in patients compared to healthy controls and in those who reported experience of childhood abuse or neglect. Multiple logistic regressions showed a strong effect of CTQ total scores and duration of symptoms as predictors of TGF-β levels in patients.
**Conclusions:** Conclusions: Childhood abuse as a predictor of TGF-β concentration in MDD patients could be an important missing puzzle in understanding immunological pathways and the role of cytokines in depression.

### O0155

#### Group rumination-focused cognitive-behavioural therapy (CBT) v. group CBT for depression: Phase II trial

M. Hvenegaard*
Denmark

**Introduction:** Although cognitive-behavioural therapy (CBT) is an effective treatment for depression, less than half of patients achieve satisfactory symptom reduction during treatment. Targeting known psychopathological processes such as rumination may increase treatment efficacy.
**Objectives:** The aim of this study was to test whether adding group rumination-focused CBT (RFCBT) that explicitly targets rumination to routine medical management is superior to add- ing group CBT to routine medical management in treating major depression.
**Methods:** A total of 131 outpatients with major depression were randomly allocated to 12 ses- sions group RFCBT v. group CBT, each in addition to routine medical management. The pri- mary outcome was observer-rated symptoms of depression at the end of treatment measured on the Hamilton Rating Scale for Depression. Secondary outcomes were rumination at post- treatment and depressive symptoms at 6 months follow-up (Trial registered: NCT02278224).

**Results:** RFCBT significantly improved observer-rated depressive symptoms (Cohen's d 0.38; 95% CI 0.03–0.73) relative to group CBT at post-treatment on the primary outcome. No post- treatment differences were found in rumination or in depressive symptoms at 6 months fol- low-up, although these secondary analyses may have been underpowered.

**Conclusions:** This is the first randomized controlled trial providing evidence of benefits of RFCBT in major depression compared with CBT. Group RFCBT may be a beneficial alterna- tive to group CBT for major depression.

**Conflict of interest:** No

### O0156

### Kynurenine pathway abnormalities in MDD and their relation to the characteristic inflammatory state of patients

G. Arteaga-Henriquez[1*], B. Burger[1,2], E. Weidinger[2], L. Grosse[2], N. Moll[2], G. Schuetze[2], M. Schwarz[2], A. Wijkhuijs[3], M. Versnel[3], V. Arolt[2], N. Müller[2] and H. Drexhage[3]

[1]Spain; [2]Germany and [3]Netherlands

**Introduction:** An activated inflammatory state in major depressive disorder (MDD) has been hypothesized to induce indoleamine 2, 3-dioxygenase 1 (IDO-1) expression, resulting in a preferential breakdown of tryptophan (TRP) to kynurenine (KYN) along the kynurenine pathway. This would explain the decreased TRP and serotonin levels that have been repeatedly found in MDD.

**Objectives:** The aim of the present study was to investigate the concept of a pro-inflammatory state in MDD as an inducer of a preferential breakdown of TRP along the kynurenine pathway.

**Methods:** In N = 281 MDD patients and N = 206 healthy controls, we determined the serum levels of TRP, 5-hydroxytryptophan (5-HTrp) and KYN using high performance liquid chromatography and/or liquid chromatography with mass spectrometry. Additionally, we analyzed potential relations between these metabolites and the expression levels of key inflammatory and kynurenine pathway enzyme genes using standard Q-PCR.

**Results:** TRP serum levels were decreased ,KYN serum levels were not raised in patients with MDD. Patient's monocytes showed a significant higher inflammatory gene expression, while IDO-1 gene expression was not upregulated in the circulating monocytes of patients. Interestingly, the expression of key inflammatory genes negatively correlated to IDO-1 gene expression in the circulating monocytes of patients.

**Conclusions:** Our observations reject the hypothesis of a preferential breakdown of systemic TRP along the kynurenine pathway in patients with MDD linked to their characteristic systemic pro-inflammatory monocyte state.

**Conflict of interest:** No

### O0159

### Does dynamic thiol/disulfide balance predict mixed symptoms of depression?

Ç. Açar Yıldızoğlu*, H.İ. Taş and E. Yıldızoğlu

Turkey

**Introduction:** Plasma thiol/disulfide levels are thought to be valuable and reliable markers of oxidative stress and antioxidant defense mechanisms. Achieving this balance plays a critical role in the pathogenesis of many diseases such as depression.

**Objectives:** The aim of this study was to evaluate the scores of oxidative stress in patients with unipolar depression (UD) and in patients with mixed type of unipolar depression (mxUD) and to compare these scores with healthy subjects in terms of dynamic thiol/disulfide balance parameters.

**Methods:** 97 patients with the diagnosis of UD and 97 healthy individuals were included in the study. Hamilton Depression Rating Scale (HAM-D) and Modified Hypomania Checklist (M-HCL) were used respectively to determine depressive symptoms, manic symptoms and the severity of the disease. Oxidative stress parameters were measured by evaluating fasting blood samples taken from all participants.

**Results:** In the UD group, the levels of disulfide, total thiol, oxidation-reduction ratio and oxidized thiol were found statistically higher in the depression group than the control group in favor of oxidation ($p<0.05$). Reduced thiol levels which are favorable for the antioxidant system, were statistically lower in the depression group than the control group ($p<0.05$). There was no significant difference between the mxUD and non-mixed featured UD in thiol-disulfide balance.

**Conclusions:** This findings showed that, the balance deteriorated in favor of oxidation or antioxidation in depression may not be a significant biomarker for mixed symptoms of depression.

**Conflict of interest:** No


Article ID: O0001
Subjects: Abstract, Oral Communication Session 01: Anxiety Disorders and Somatoform Disorders / Bipolar Disorders / Classification of Mental Disorders

Further validation of the portuguese version of the modified dental anxiety scale

Pereira A.T.
Araújo A.
Cabaços C. *
Soares M.J.
Xavier S.
Marques C.
Lopes P.
Macedo A.
Portugal
* Corresponding author.
Keywords: Dental Anxiety

Conflict of interest:

No

Article ID: O0002

Impact of childhood trauma and attachment styles on resilience in euthymic patients with bipolar disorder

Citak C. *
Erten E.
Turkey
* Corresponding author.
Keywords: Bipolar disorder, childhood maltreatment, Resilience, attachment

Conflict of interest:

No

Article ID: O0007

Evaluating maintenance electroconvulsive therapy for relapse prevention in bipolar disorder

Madero S. *
Anmella G.
Pons M.T.
Sagué M.
Palomo A. Giménez
Murru A.
Arbelo N.
Llach C.
Ilzarbe L.
Bioque M.
Benabarre A.
Spain
* Corresponding author.
Keywords: Relapse prevention, Bipolar Disorders, Maintenance electroconvulsive therapy

Conflict of interest:

No

Article ID: O0008

Sexual dysfunction in bipolar disorder: A systematic review

Pérez Y. Cañada 1 *
Rodríguez P. Navalón 1
Zazula R. 2
Berk M. 3
Dodd S. 3
García-Blanco A. 1
Miguel P. Sierra San 1
1 Spain ,
2 Brazil ,
3 Australia
* Corresponding author.
Keywords: euthymia, Bipolar disorder, sexual dysfunction

Article ID: O0009

Depressive polarity at illness onset is associated with lifetime suicide attempts in euthymic bipolar outpatients

Serafini G. 1 *
Aguglia A. 1
Santi F. 1
Canepa G. 1
Gonda X. 2
Rihmer Z. 2
Pompili M. 1
Amore M. 1
1 Italy ,
2 Hungary
* Corresponding author.
Keywords: Bipolar disorder, Age at illness onset, first depressive episode, Melancholic features

Conflict of interest:

No

Article ID: O0010

Predicting patient outcomes in psychiatric hospitals with routine data: A machine learning approach

Wolff J. *
Gary A.
Jung D.
Normann C.
Kaier K.
Binder H.
Domschke K.
Klimke A.
Franz M.
Germany
* Corresponding author.
Keywords: Hospitals, Decision Support Techniques, Machine Learning, Predictions

Article ID: O0014
Subjects: Abstract, Oral Communication Session 02: Psychopathology / Schizophrenia and Other Psychotic Disorders - Part I

Validity of the spanish adult ADHD self-report scale (ASRS) expanded scale and its relationship to whole-life self-reported psychopathology

Artuch-Garde R.
Aznárez-Sanado M.
Bernacer J. *
Gambra L.
Magallón S.
Garcia-Arbizu I.
Vallejo-Valdivielso M.
Carrica-Ochoa S.
Arrondo G.
Spain
* Corresponding author.
Keywords: psychometry, validation, ASRS, ADHD

Conflict of interest:

No

Article ID: O0015

Neurocognition and social cognition in delusional disorder: A systematic review

González-Rodríguez A. *
Pedrero A. Álvarez
Delgado A. Guàrdia
Sanz N.
Acebillo S.
Monreal J.A.
Vidal D. Palao
Arias J. Labad
Spain
* Corresponding author.
Keywords: Delusional disorder, neurocognition, psychosis, Social Cognition

Article ID: O0019

A network model of the relationship between loneliness, paranoia, and emotional disturbances

Chau A.K.C. *
So S.H.-W.
Hong Kong PRC
* Corresponding author.
Keywords: perceived social isolation, Persecutory delusions, schizotypy, network analysis

Conflict of interest:

No

Article ID: O0020

Self- and caregiver-reported disability after one year of LAI antipsychotic treatment in schizophrenia: Preliminary results

D’Anna G. *
Tatini L.
Ricca V.
Ballerini A.
Italy
* Corresponding author.
Keywords: schizophrenia, antipsychotics, disability, WHODAS 2.0

Conflict of interest:

No

Article ID: O0022

Variation of negative symptoms in people with schizophrenia after adding aripiprazole to the treatment with risperidone or paliperidone monotherapy

Arnaiz A.
Olivas O.
Erkoreka L. *
Arrue A.
Barreiro A.
Basterreche N.
Zamalloa M.I.
Zumarraga M.
Spain
* Corresponding author.
Keywords: risperidone/paliperidone, aripiprazole, negative symptoms, schizophrenia

Conflict of interest:

No

Article ID: O0023
Subjects: Abstract, Oral Communication Session 03: Genetics & Molecular Neurobiology - Part I / Neuroimaging / Neuroscience in Psychiatry

Pharmacodynamic analysis of polymorphisms of DRD2 and HTR1A genes in antischizophrenic drugs

Zhang Y. *
Cui X.
China
* Corresponding author.
Keywords: Antischizophrenic Drugs, PANSS, DRD2, HTR1A

Conflict of interest:

No

Article ID: O0024

An in-depth analysis of DNA methylation in three genetic LOCI shared between schizophrenia and intelligence

Alfimova M.
Kondratyev N.
Golov A.
Lezheiko T. *
Golimbet V.
Russian Federation
* Corresponding author.
Keywords: schizophrenia, cognitive deficit, epigenetics, methylation

Conflict of interest:

No

Article ID: O0025

The study of effects of oxytocin pathway genes and early-life stressors on social functioning in schizophrenia

Lezheiko T. *
Smirnova S.
Mikhailova V.
Krikova E.
Kolesina N.
Asanov A.
Golimbet V.
Russian Federation
* Corresponding author.
Keywords: schizophrenia, gene-environment interactions, Oxytocin, social functioning

Conflict of interest:

No

Article ID: O0031

Intracranial volume and subcortical structures in adolescents with early-onset psychosis: A mega-analysis from the enigma early-onset psychosis working group

Agartz I. 1 *
Gurholt T. 1
Nerland S. 1
Thompson P. 2
Andreassen O. 1
1 Norway
2 United States of America
* Corresponding author.
Keywords: Early-onset psychosis disorder, neuroimaging, adolescence, antipsychotics

Conflict of interest:

No

Article ID: O0032

Resting-state FMRI correlates of clinical response to stimulant treatments in children and adolescents with ADHD

Pereira-Sanchez V. 1 *
Franco A. 1
De Castro-Manglano P. 2
Vallejo-Valdivielso M. 2
Diez-Suarez A. 1 2
Fernández-Seara M. 2
Fernandez-Martinez M. 2
Milham M. 1
Castellanos F. 1
1 United States of America
2 Spain
* Corresponding author.
Keywords: ADHD, fMRI, stimulants, resting-state

Conflict of interest:

No

Article ID: O0033

Dysfunctional brain activity and the inability to delay gratification in obesity with or without binge eating disorder

Miranda-Olivos R. 1 *
Steward T. 2
Martínez-Zalacaín I. 1
Testa G. 1
Jimenez-Murcia S. 1
Soriano-Mas C. 1
Fernandez-Aranda F. 1
1 Spain
2 Australia
* Corresponding author.
Keywords: obesity, binge eating disorder, Delay discounting, Functional neuroimaging

Conflict of interest:

No

Article ID: O0035
Subjects: Abstract, Oral Communication Session 04: Child and Adolescent Psychiatry - Part I

Tunisian validation of a cyberbullying assessment instrument: The « second revision of the revised cyberbullying inventory »

Sahli L.
Bourgou S. *
Amor S. Haj
Fakhfakh R.
Belhadj A.
Tunisia
* Corresponding author.
Keywords: validation, questionnaire

Conflict of interest:

No

Article ID: O0036

A controlled pharmaco-imaging trial of memantine for the treatment of social deficits in adolescents with high-functioning autism spectrum disorder

Joshi G. *
United States of America
* Corresponding author.
Keywords: Memantine, Autism, Pharmaco-Imaging

Article ID: O0037

Externalising psychopathology and suicidal behavior during adolescence: A 17 years population based study

Forte A. 1 *
Orri M. 2
Pompili M. 1
Galera C. 3
Trambley R. 2
Côté S. 2
1 Italy
2 Canada
3 France
* Corresponding author.
Keywords: Hyperactivity, Inattention, Suicidal behavior, Developmental trajectories

Conflict of interest:

No

Article ID: O0040

Influence of 3-omega fatty acids (FA) on clinical status and inflammatory markers in depressive disorder in children

Trebaticka J. 1 *
Surovcova A. 1
Matzova Z. 1
Hradečna Z. 1
Vavakova M. 1
Garaiova I. 2
Šuba J. 1
Ďuračkova Z. 1
1 Slovak Republic
2 United Kingdom
* Corresponding author.
Keywords: omega fatty acids, Children, Dépression

Conflict of interest:

No

Article ID: O0041

Radical thinking predicts socially desirable appraisals of self-regulation in adolescents with delinquent behavior

Tkhostov A. *
Rasskazova E.
Falkovskaia L.
Kiseleva A.
Emelin V.
Russian Federation
* Corresponding author.
Keywords: self-regulation, delinquent behavior, adolescents, “black-and-white” thinking

Article ID: O0043

Online multitasking in adolescents: Important skill or sign of internet addiction?

Soldatova G.
Rasskazova E.
Vinitskiy D. *
Chigarkova S.
Russian Federation
* Corresponding author.
Keywords: excessive Internet use, digital competence, multitasking

Article ID: O0044

Autism spectrum disorders in a population of very low birth weght: Causes and consequences

Uccella S. *
De Grandis E.
Dufour E.
Tortora D.
Severino M.
Malova M.
Nobili L.
Ramenghi L.A.
Italy
* Corresponding author.
Keywords: prematurity, autism spectrum disorders, neurodevelopment, Inflammation

Conflict of interest:

No

Article ID: O0047
Subjects: Abstract, Oral Communication Session 05: Cultural Psychiatry / Mental Health Care / Training in Psychiatry / Others

Patient-reported satisfaction in psychiatry admission in a tertiary-care teaching hospital in nepal: A descriptive cross-sectional study

Joshi S. *
Thakur T.
Shakya R.
Nepal
* Corresponding author.
Keywords: Health Care Costs, Inpatients, mental illness, Personal satisfaction

Conflict of interest:

No

Article ID: O0048

Outcomes from the IBBIS randomized controlled trial (n=611): Integrated vocational rehabilitation and mental health care for people with anxiety and depression

Hoff A. *
Fisker J.
Nordentoft M.
Poulsen R.
Hjorthøj C.
Eplov L.
Denmark
* Corresponding author.
Keywords: Dépression, Anxiety, Vocational rehabilitation, Integrated Care

Conflict of interest:

No

Article ID: O0049

Relationship between maternal mindfulness training during pregnancy and executive abilities in children

Kiselev S. *
Russian Federation
* Corresponding author.
Keywords: mindfulness training, executive abilities, NEPSY, Anxiety

Conflict of interest:

No

Article ID: O0051

Substance abuse in acute psychiatric unit inpatients

Procenesi L.
Colasuonno R. *
Brogna P.
Talamo A.
Di Michele F.
Niolu C.
Siracusano A.
Italy
* Corresponding author.
Keywords: Acute Psychiatric Unit, SUD, psychiatric comorbilities

Conflict of interest:

No

Article ID: O0052

Overview of the phenomenon of violence against psychiatric trainees in europe: The EFPT-VAPT study

Pereira-Sanchez V. * 1
Gürcan A. 2
Erzin G. 2
Rai Y. 3
Gnanavel S. 3
Fontaine A. 4
Vieira J. 5
Asztalos M. 6
Szczegielniak A. 7
1 United States of America ,
2 Turkey ,
3 United Kingdom ,
4 France ,
5 Portugal ,
6 Denmark ,
7 Poland
* Corresponding author.
Keywords: international survey, European Federation of Psychiatric Trainees, workplace violence, training in psychiatry

Conflict of interest:

No

Article ID: O0057
Subjects: Abstract; Oral Communication Session 06: Emergency Psychiatry / Psychopharmacology and Pharmacoeconomics / Psychosurgery & Stimulation Methods (Ect, Tms, Vns, Dbs) / Rehabilitation and Psychoeducation

Trends in prevalence and mean doses of commonly used antipsychotic drugs in scandinavia: A multi-national drug utilization study

Højlund M. 1 *
Pottegård A. 1
Johnsen E. 2
Kroken R. 2
Reutfors J. 3
Munk-Jørgensen P. 1
Correll C. 4
1 Denmark ,
2 Norway ,
3 Sweden ,
4 United States of America
* Corresponding author.
Keywords: pharmacoepidemiology, drug utilization, psychopharmacology, antipsychotics

Conflict of interest:

No

Article ID: O0060

Metabolic and cardiovascular effects of antipsychotic drugs. A meta-analysis of randomized controlled trials

Cassioli E. *
Calderani E.
Lazzeretti L.
Ragghianti B.
Ricca V.
Mannucci E.
Rotella F.
Italy
* Corresponding author.
Keywords: meta-analysis, antipsychotics, Side Effects, cardiovascular diseases

Conflict of interest:

No

Article ID: O0061

1Hz rTMS to the SMA added to iTBS of the LDLPFC enhances response in highly ruminative refractory depression

Davila J. 1
Cruz F. 1
Mejia C. 1
Davila G. * 1
Davila I. 2
1 Colombia ,
2 United States of America
* Corresponding author.
Keywords: Refractory Depression, iTBS, ruminations, rTMS

Conflict of interest:

No

Article ID: O0062

Early cognitive improvement as a predictor of rTMS efficacy in schizophrenic patients with depression

Maslenikov N. *
Tsukarzi E.
Mosolov S.
Russian Federation
* Corresponding author.
Keywords: rTMS, schizophrenia, Dépression, cognitive functions

Conflict of interest:

No

Article ID: O0065

Psychological factors of effective rehabilitation after sport trauma: Validation of the illness and treatment self-regulation questionnaire

Yavorovskaia A.
Rasskazova E. *
Leonov S.
Russian Federation
* Corresponding author.
Keywords: rehabilitation, sport trauma, Illness and Treatment Self-regulation Questionnaire

Article ID: O0067
Subjects: Abstract; Oral Communication Session 07: Women, Gender and Mental Health / Ethics and Psychiatry / Intellectual Disability

Mother infant feeding interaction (MI-FI): Improved eating habits at 1 year

Latzer Y. *
Rozen G.
Elad I.
Globus I.
Israel
* Corresponding author.
Keywords: hunger satiety cues, Early parent-child relationship, feeding interaction, infant eating habits

Conflict of interest:

No

Article ID: O0071

Prescriptions of psychotropic medications among finnish women using hormonal contraception

Toffol E. *
Partonen T.
Heikinheimo O.
Latvala A.
But A.
Haukka J.
Finland
* Corresponding author.
Keywords: hormonal contraception, psychotropic medication, prescription

Conflict of interest:

No

Article ID: O0072

Pharmacokinetics of lithium in nursing infants

Imaz M.L. *
Torra M.
Soy D.
Garcia-Esteve L.
Martin-Santos R.
Spain
* Corresponding author.
Keywords: Lithium, Plarmacokinetics, Lactation, Nursing Infants

Conflict of interest:

No

Article ID: O0073

Discriminant validity of the prenatal obsessive-compulsive scale – distinguishing between perinatal OC, depression and anxiety in pregnancy

Pereira A.T.
Araújo A. *
Xavier S.
Azevedo J.
Soares M.J.
Marques M.
Macedo A.
Portugal
* Corresponding author.
Keywords: Prenatal obsessive-compulsive symptoms, Anxiety, Perinatal Obsessive-Compulsive Scale, Dépression

Conflict of interest:

No

Article ID: O0074

Empathy and personality traits in general practitioners

Mnif D. *
Sellami R.
Jmal D.
Tunisia
* Corresponding author.
Keywords: general practitioner, Empathy, personnality, therapeutic alliance

Conflict of interest:

No

Article ID: O0077
Subjects: Abstract, Oral Communication Session 08: Prevention of Mental Disorders / Schizophrenia and Other Psychotic Disorders - Part II

Clinical and biological signs of the schizophrenia prodrome

Omelchenko M. *
Sarmanova Z.
Zozulya S.
Kaleda V.
Russian Federation
* Corresponding author.
Keywords: biological markers, depression in youth, Schizophrenia prodrome

Conflict of interest:

No

Article ID: O0078

Experience of cyberbullying predicts hostility and interpersonal anxiety in youth and adults

Soldatova G.
Vinitskiy D. *
Rasskazova E.
Chigarkova S.
Russian Federation
* Corresponding author.
Keywords: hostility, interpersonal anxiety, cyberbullying

Article ID: O0082

Mthfr polymorphisms as possible markers of treatment response in first-episode psychosis

Kuharic D. Bosnjak *
Makaric P.
Sabo T.
Ostojic D.
Silic A.
Savic A.
Brečić P.
Prpic N.
Kuzman M. Rojnic
Croatia
* Corresponding author.
Keywords: PSYCHOPATOLOGY, MHTFR, first-episode psychosis, follow-up

Conflict of interest:

No

Article ID: O0083

Efficacy of cariprazine in the treatment of acute and primary negative symptoms of schizophrenia: Posthoc analyses versus risperidone

Sebe B. 1 *
Barabássy Á. 1
Balogh Á. 1
Acsai K. 1
Laszlovszky I. 1
Szatmári B. 1
Harsányi J. 1
Patel M. 2
Earley W. 2
Németh G. 1
1 Hungary
2 United States of America
* Corresponding author.
Keywords: Cariprazine, Acute symptoms, schizophrenia, negative symptoms

Article ID: O0085

Verbal fluency of patients with paranoid schizophrenia, first grade schizophrenia relatives and healthy controls – comparative study

Stoimenova-Popova M. *
Veleva I.
Chumpalova P.
Tumbev L.
Yakimova R.
Dimitrova E.
Krasteva E.
Valkova M.
Bulgaria
* Corresponding author.
Keywords: healthy controls, schizophrenia, Verbal fluency, first grade schizophrenia relatives

Conflict of interest:

No

Article ID: O0086

Cognitive function in patients with schizophrenia and affective disorder: Effects of combining pharmacotherapy with cognitive remediation

Erfurth A. *
Maihofer E.
Sachs G.
Austria
* Corresponding author.
Keywords: schizophrenia, affective disorder, cognitive remediation, Cognition

Conflict of interest:

No

Article ID: O0088
Subjects: Abstract, Oral Communication Session 10: Depressive Disorders - Part I

Baseline EEG beta spectral power predicts the response to treatment in depressive patients

Iznak A. *
Iznak E.
Abramova L.
Kobel'Kov G.
Russian Federation
* Corresponding author.
Keywords: Dépression, pre-treatment EEG, prediction of therapeutic response, depression, pre-treatment EEG, prediction of therapeutic response

Article ID: O0089

SAGE-217 in major depressive disorder: A phase 3, multicenter, double-blind, randomized, placebo-controlled trial

Clayton A. *
Lasser R.
Nandy I.
Sankoh A.
Campbell D.
Werneburg B.
Silber C.
Jonas J.
Kanes S.
Gunduz-Bruce H.
United States of America
* Corresponding author.
Keywords: major depressive disorder, GABA, positive allosteric modulator, SAGE-217

Article ID: O0091

The safety, efficacy, and tolerability of a microbial therapeutic in people with major depression and/or generalized anxiety disorder: Preliminary findings

Meyyappan A. Chinna *
Milev R.
Canada
* Corresponding author.
Keywords: Dépression, Microbiome, MET-2, gut-brain axis

Conflict of interest:

No

Article ID: O0092

Depression and non-adherence to medications targeting treatable cardiovascular risk factors in the constances cohort

Hamieh N. *
Kab S.
Zins M.
Goldberg M.
Melchior M.
Lemogne C.
France
* Corresponding author.
Keywords: Dépression, Adherence, cardiovascular risk factors, Medications

Conflict of interest:

No

Article ID: O0094

Signs of early monocyte aging and mevalonate kinase abnormality underlying the full-blown overexpression of monocyte inflammatory genes in major depressive disorder patients after childhood adversity

Simon M. 1 *
Schiweck C. 2
Wijkhuijs A. 3
Claes S. 2
Arolt V. 1
Müller N. 1
Drexhage H. 3
1 Germany
2 Belgium
3 Netherlands
* Corresponding author.
Keywords: major depressive disorder, monocyte gene expression, Inflammation, apoptosis

Conflict of interest:

No

Article ID: O0097

Enhancing the detection of postpartum depression from electronic health records using machine learning algorithms

Amit G. 1 *
Girshovitz I. 1
Akiva P. 1
Bar V. 1
Zhang Y. 2
Hermann A. 2
Joly R. 2
Turchioe M. 2
Pathak J. 2
1 Israel
2 United States of America
* Corresponding author.
Keywords: postpartum depression, Machine Learning, electronic health records

Conflict of interest:

No

Article ID: O0099
Subjects: Abstract, Oral Communication Session 11: Comorbidity / Dual Pathologies / Addictive Disorders

Mental disorders with damage to the diencephalic region

Sidneva Y. *
Astaf'Eva L.
Zaitsev O.
Kalinin P.
Kutin M.
Kadashev B.
Voronina I.
Shkarubo A.
Fomichev D.
Sharipov O.
Andreev D.
Russian Federation
* Corresponding author.
Keywords: craniopharyngioma, mental disorders, Korsakoff’s syndrome, behavioral, emotional and personality disorders

Conflict of interest:

No

Article ID: O0105

Problematic gambling, gaming and internet behavior in heterosexual and sexual minority women

Broman N. 1 *
Håkansson A. 1
Prever F. 2
Di Giacomo E. 2
Jimenez-Murcia S. 3
Szczegielniak A. 4
1 Sweden
2 Italy
3 Spain
4 Poland
* Corresponding author.
Keywords: Behavioral addiction, sexual minority women, Gambling, Gaming

Article ID: O0107

Betting on yourself is never a gamble: Cannabis users fail to take advantage of favourable opportunities in a novel gambling paradigm

Perkins S. *
Lim T.V.
Ersche K.
United Kingdom
* Corresponding author.
Keywords: THC, Early intervention, Decision making, Community population

Article ID: O0110
Subjects: Abstract, Oral Communication Session 09: Consultation Liaison Psychiatry and Psychosomatics / Epidemiology and Social Psychiatry / Child and Adolescent Psychiatry - Part II

Chronic physical illness, multimorbidity and their effects on psychiatric treatment outcomes

Filipčić I. *
Filipčić I. Šimunović
Croatia
* Corresponding author.
Keywords: CHRONIC PHYSICAL ILLNESS, MULTIMORBIDITY, treatment outcome, schizophrenia

Conflict of interest:

No

Article ID: O0116

Behavioral and emotional problems in a large group of italian adolescents

Lisi G. *
Rossi R.
Ribolsi M.
Di Lorenzo G.
Parisi C.
Siracusano M.
Morciano L.
De Stefano A. Alberto
Pesaresi A.
Niolu C.
Palombi L.
Siracusano A.
Italy
* Corresponding author.
Keywords: adolescence, mental health, externalising symptoms

Conflict of interest:

No

Article ID: O0119

In-utero patterns of antidepressant use and cognitive development at 3 years of age: Results from the mothertobaby antidepressants study

Berard A. *
Tchuente V.
Sheehy O.
Zhao J.
Canada
* Corresponding author.
Keywords: In-Utero Exposure, Children's cognitive development at 3 years, MotherToBaby Antidepressants Study, Antidepressants

Conflict of interest:

No

Article ID: O0120

Sex-specific volumetric variations associated with alcohol use in adolescence: A longitudinal neuroimaging study

Navarri X. *
Filippi I.
Conrod P.
Canada
* Corresponding author.
Keywords: Neuroimaging; Alcohol; adolescence, longitudinal

Conflict of interest:

No

Article ID: O0123
Subjects: Abstract, Oral Communication Session 12: Genetics & Molecular Neurobiology - Part II / Psychoneuroimmunology / Research Methodology

Exploring epigenetic mechanisms linking childhood adversity and psychosis in patients with first episode of psychosis – data from the EUGEI study

Alameda L. *
Aas M.
Rodríguez V.
Kandaswamy R.
Quattrone D.
Wong C.
Gayer-Anderson C.
Morgan C.
Di Forti M.
Murray R.
United Kingdom
* Corresponding author.
Keywords: Psychosis, childhood trauma, epigenetics, DNA-Methylation

Conflict of interest:

No

Article ID: O0124

Enriched developmental biology molecular pathways impacts on antipsychotics induced weight gain

Drago A. *
Corfitsen H.
Denmark
* Corresponding author.
Keywords: Pharmacogenetics, Side Effects, weight gain, molecular pathway analysis

Conflict of interest:

No

Article ID: O0126

Copy number variation of satellite III (1Q12) in patients with schizophrenia

Kaydan M. *
Zakharova N.
Ershova E.
Shmarina G.
Agafonova O.
Bravve L.
Jestkova E.
Martynov A.
Veiko R.
Umriukhin P.
Kostyuk G.
Kutsev S.
Veiko N.
Kostyuk S.
Russian Federation
* Corresponding author.
Keywords: Schizophrenia, Genetics, satellite

Conflict of interest:

No

Article ID: O0127

Cytokine levels in schizophrenia patients with metabolic syndrome

Boiko A. *
Mednova I.
Kornetova E.
Ivanova S.
Russian Federation
* Corresponding author.
Keywords: metabolic syndrome, Inflammation, cytokines profile, schizophrenia

Article ID: O0128

Classification of first episode psychosis and chronic psychosis patients using the immunological transcriptome profile in a large cohort of patients: A machine learning analysis

Enrico P. *
Delvecchio G.
Colombo G.
Turtulici N.
Villa F.M.
Finardi A.
Furlan R.
Brambilla P.
Italy
* Corresponding author.
Keywords: Neuropsychoimmunology, Machine Learning, Inflammation, psychosis

Conflict of interest:

No

Article ID: O0130

Culturally equivalent translation into spanish of the 31-item adult ADHD self-report scale (ASRS)

Mertz-Echauri E. *
Bernacer J.
Gambra L.
Magallón S.
Garcia-Arbizu I.
Vallejo-Valdivielso M.
Arrondo G.
Spain
* Corresponding author.
Keywords: ADHD, Validation, Translation, ASRS

Conflict of interest:

No

Article ID: O0131

Reactivity intensity polarity and stability scale (RIPOST): Statistical validation of a literary arabic version

Allaya A. *
Braham A.
Rejeb H. Ben
Chebbi W.
Nakhli J.
Nasr S. Ben
Tunisia
* Corresponding author.
Keywords: literary Arabic, RIPoST, statistical validation

Conflict of interest:

No

Article ID: O0132
Subjects: Abstract, Oral Communication Session 13: Posttraumatic Stress Disorder / Suicidology and Suicide Prevention / Oncology and Psychiatry

Oral communication session 13: Posttraumatic stress disorder / suicidology and suicide prevention / oncology and psychiatry

Grunberg K. *
Stoltenberg J.
Miller C.
United States of America
* Corresponding author.
Keywords: Neuroimaging, meta-analysis, fMRI, PTSD

Conflict of interest:

No

Article ID: O0135

Suicidal risk in mood disorders: Do different types of childhood adversity differentially predict suicidal ideation, planning, and attempts?

Cassis T. *
Young E.
Low N.
Canada
* Corresponding author.
Keywords: childhood adversity, Mood disorders, Bipolar, Suicidality

Conflict of interest:

No

Article ID: O0136

Suicidal ideation and suicide attempts in obsessive-compulsive related disorders (OCRDs): A systematic review and meta-analysis

Pellegrini L. 1 *
Fineberg N. 2
Menchetti M. 1
Berardi D. 1
Rucci P. 1
Maina G. 1
Rihmer Z. 3
Albert U. 1
1 Italy
2 United Kingdom
3 Hungary
* Corresponding author.
Keywords: Obsessive-Compulsive Related Disorders, suicide risk, Suicidal ideation, SUICIDE ATTEMPTS

Conflict of interest:

No

Article ID: O0137

Suicidal ideation and suicide attempts in patients with obsessive-compulsive disorder: A systematic review and meta-analysis

Pellegrini L. 1 *
Fineberg N. 2
Maina G. 1
Rucci P. 1
Albert U. 1
1 Italy
2 United Kingdom
* Corresponding author.
Keywords: Suicide prevention, SUICIDE ATTEMPTS, obsessive-compulsive disorder, suicide risk

Conflict of interest:

No

Article ID: O0141

Longitudinal relations between insomnia and suicide attempts in adolescence and young adulthood

Alvarez K. *
Gonzalez M. Cruz
Bancalari P.
Alegria M.
Roberts R.
United States of America
* Corresponding author.
Keywords: Suicide, İnsomnia, suicidal behavior, adolescence

Conflict of interest:

No

Article ID: O0143
Subjects: Abstract, Oral Communication Session 15: Eating Disorders / E-Mental Health

Evaluation of efficacy of social cognitive training in improvement of socioemotional processes throughout therapeutic process in an inpatients

Kot E. *
Kostecka B.
Wayda-Zalewska M.
Kucharska K.
Poland
* Corresponding author.
Keywords: social cognition anorexia nervosa

Conflict of interest:

No

Article ID: O0144

The role of disgust sensibility and propensity in eating disorders patients with traumatic history

Innocenti M. *
Gironi V.
Giaquinta N.
Angeletti L. Lucherini Bargellini
Galassi F.
Castellini G.
Ricca V.
Italy
* Corresponding author.
Keywords: disgust sensitivity and propensity, anxiety disorders, eating disorders, disgust

Conflict of interest:

No

Article ID: O0145

Impact of cognitive behavioral therapy on cortisol levels in patients with eating disorders and childhood abuse: A follow-up study

Cassioli E. *
Rossi E.
Lelli L.
Castellini G.
D'Anna G.
Monteleone A.M.
Ricca V.
Italy
* Corresponding author.
Keywords: eating disorders, childhood abuse, cortisol, cognitive behavioral therapy

Conflict of interest:

No

Article ID: O0146

Genetic, neural and phenotypic predictors of eating disorder symptoms and comorbid mental health in adolescence and early adulthood

Desrivieres S. *
Robinson L.
Zhang Z.
Schmidt U.
United Kingdom
* Corresponding author.
Keywords: Eating disorders 'add' genetic 'add' brain 'add' biomarker

Conflict of interest:

No

Article ID: O0147

The validity of the 5th BMI percentile as weight cut-off for anorexia nervosa in children and adolescents: No evidence from a psychopathology investigation

Cascino G. *
Mereu A.
Zanna V.
Monteleone P.
Vicari S.
Monteleone A.M.
Italy
* Corresponding author.
Keywords: Anorexia nervosa, network analysis, Body mass index, adolescence

Conflict of interest:

No

Article ID: O0148

Maudsley learning podcast: The innovation, implementation and appraisal of a technological advance in psychiatric education - an accessible tool for both clinical and personal reflection

Curmi A. *
United Kingdom
* Corresponding author.
Keywords: Inter-disciplinary, Philosophy, Education, Technology

Conflict of interest:

No

Article ID: O0150
Subjects: Abstract, Oral Communication Session 14: Obsessive-Compulsive Disorder / Sleep Disorders & Stress / Depressive Disorders - Part II

Application of a staging model to a clinical sample of italian obsessive-compulsive patients

Benatti B. *
Girone N.
Fesce F.
Lucca G.
Vismara M.
De Carlo V.
Grancini B.
Donà C.
Casati L.
Ferrari S.
Virzì C.
Colombo A.
Bosi M.
Viganò C.
Dell'Osso B.
Italy
* Corresponding author.
Keywords: obsessive compulsive disorder, staging, duration of untreated illness, severity

Conflict of interest:

No

Article ID: O0151

Assessment of over valuated thoughts, metacognition, magical beliefs and quality of life in patients with obsessive compulsive disorder and subtypes

Bakir M.G. Teksin *
Aslan S.
Turkey
* Corresponding author.
Keywords: magical beliefs, over valuated thoughts, metacognition, obsessive compulsive disorder

Conflict of interest:

No

Article ID: O0153

Transforming growth factor beta and childhood abuse in adults with major depressive disorder

Munjiza A. *
Kostic M.
Kosutic Z.
Voncina M. Mitkovic
Pesic D.
Todorovic D.
Markovic I.
Tosevski D. Lecic
Serbia
* Corresponding author.
Keywords: Transforming growth factor beta, major depressive disorder, childhood abuse

Conflict of interest:

No

Article ID: O0155

Group rumination-focused cognitive-behavioural therapy (CBT) v. group CBT for depression: Phase II trial

Hvenegaard M. *
Denmark
* Corresponding author.
Keywords: Psychiatry; Dépression; Rumination,; CBT

Conflict of interest:

No

Article ID: O0156

Kynurenine pathway abnormalities in MDD and their relation to the characteristic inflammatory state of patients

Arteaga-Henriquez G. 1 *
Burger B. 1 2
Weidinger E. 2
Grosse L. 2
Moll N. 2
Schuetze G. 2
Schwarz M. 2
Wijkhuijs A. 3
Versnel M. 3
Arolt V. 2
Müller N. 2
Drexhage H. 3
1 Spain
2 Germany
3 Netherlands
* Corresponding author.
Keywords: Tryptophan, major depressive disorder, Inflammation, kynurenine pathway

Conflict of interest:

No

Article ID: O0159

Does dynamic thiol/disulfide balance predict mixed symptoms of depression?

Yıldızoğlu Ç. Açar *
Taş H.İ.
Yıldızoğlu E.
Turkey
* Corresponding author.
Keywords: Dépression, thiol disulfide balance, mixed features

Conflict of interest:

No

